# Highly Aromatic Flavan-3-ol Derivatives from Palaeotropical *Artocarpus lacucha* Buch.-Ham Possess Radical Scavenging and Antiproliferative Properties

**DOI:** 10.3390/molecules26041078

**Published:** 2021-02-18

**Authors:** Weerasak Songoen, Witthawat Phanchai, Lothar Brecker, Dominik Wenisch, Michael A. Jakupec, Wanchai Pluempanupat, Johann Schinnerl

**Affiliations:** 1 Special Research Unit for Advanced Magnetic Resonance, Department of Chemistry and Center of Excellence for Innovation in Chemistry, Faculty of Science, Kasetsart University, Bangkok 10900, Thailand; weerasak238@gmail.com; 2 Department of Organic Chemistry, Faculty of Chemistry, University of Vienna, Währinger Strasse 38, A-1090 Vienna, Austria; lothar.brecker@univie.ac.at; 3 Department of Physics, Faculty of Science, Khon Kaen University, Khon Kaen 40002, Thailand; witthawat_p@kkumail.com; 4 Institute of Inorganic Chemistry, Faculty of Chemistry, University of Vienna, Währinger Strasse 42, A-1090 Vienna, Austria; dominik.wenisch@univie.ac.at (D.W.); michael.jakupec@univie.ac.at (M.A.J.); 5 Department of Botany and Biodiversity Research, Faculty of Life Science, University of Vienna, Rennweg 14, A-1030 Vienna, Austria

**Keywords:** *Artocarpus lacucha*, Moraceae, flavan-benzofuran, artocarpinol, radical scavenging activities, cytotoxicity

## Abstract

Phytochemical investigation of leaves and stembark of *Artocarpus lacucha* collected in Thailand resulted in three yet undescribed isomeric flavan-3-ol derivatives (**1**–**3**), the four known compounds gambircatechol (**4**), (+)-catechin (**5**), (+)-afzelechin (**6**) and the stilbene oxyresveratrol (**7**). Compounds **1** to **3** feature 6/6/5/6/5/6 core structures. All structures were deduced by NMR and MS, while density functional theory (DFT) calculations on B3LYP theory level were performed of compounds **1** to **3** to support the stereochemistry in positions 2 and 3 in the C-ring. Possible biosynthetic pathways leading to **4** are discussed. The DPPH assay revealed high radical scavenging activities for **1** (EC_50_ = 9.4 ± 1.0 µmol mL^−1^), **2** (12.2 ± 1.1), **3** (10.0 ± 1.5) and **4** (19.0 ± 2.6), remarkably lower than ascorbic acid (EC_50_ = 34.9) and α-tocopherol (EC_50_ = 48.6). A cytotoxicity assay revealed moderate but consistent antiproliferative properties of **1** in CH1/PA-1 (ovarian teratocarcinoma) and SW480 (colon carcinoma) cells, with IC_50_ values of 25 ± 6 and 34 ± 4 µM, respectively, whereas effects in A549 (non-small cell lung cancer) cells were rather negligible. The performed DCFH-DA assay of **1** in the former cell lines confirmed potent antioxidative effects even in the cellular environment.

## 1. Introduction

*Artocarpus lacucha* Buch.-Ham. (syn *A. lakoocha* Wall. ex Roxb.; Mulberry family; Moraceae) is a widespread tree species in South-East Asia [1,2,3]. This species is also known under the vernacular names Mahat or Ma-Haad in Thailand. Other well-known and important species of this genus are *A. altilis* (Parkinson) Fosberg and *A. heterophyllus* Lam., both are important fruit trees in tropical areas.

Despite its wide distribution in natural forests and common uses for ethnomedicinal purposes, e.g., against tapeworm infections and also as component in whitening solutions [4], *A. lacucha* has not been extensively studied in a phytochemical view. A couple of studies deal with bioactivities of crude plant extracts [5,6], whereas in comparison, only some investigations of purified compounds are reported. Recently, deoxybenzoin and flavan derivatives were published to be present in this species [7,8]. Earlier, Puntumchai et al. [9] reported prenylated stilbenoid derivatives and Sritularak et al. [10] published arylbenzofurans from the root bark of this species. Among stilbenoids, oxyresveratrol possesses a wide range of bioactivities [4,11,12,13] and is accumulated in higher amounts in the heartwood of *A. lacucha* [14]. However, apart from that, isolectins were also reported from seeds of this species [15]. Within the genus *Artocarpus*, accumulation of metabolites derived from the shikimate pathway seems to be predominant. In particular, compounds possessing prenyl- and/or geranyl side chains are a quite common feature of this plant group. For example, prenylated flavonoids were reported and later summarized [16].

Herein we report the results of a phytochemical investigation on *A. lacucha*, which led to the identification of three hitherto not yet described flavan-3-ol derivatives named as artocarpinol A, 3-*epi*-artocarpinol A and artocarpinol B (**1**–**3**), together with gambircatechol (**4**), (+)-catechin (**5**) and (+)-afzelechin (**6**) from the methanolic leaf extract. The stilbene oxyresveratrol (**7**) was further purified from the stem bark extract. From compounds **1**–**4** the antioxidative properties were determined and compound **1** was investigated with respect to the cytotoxic activities against three human cancer cell lines. Additionally, the intracellular effects of the latter compound on radical oxygen species (ROS) levels were assessed by means of the fluorimetric DCFH-DA assay.

## 2. Results and Discussion

**Figure 1:**
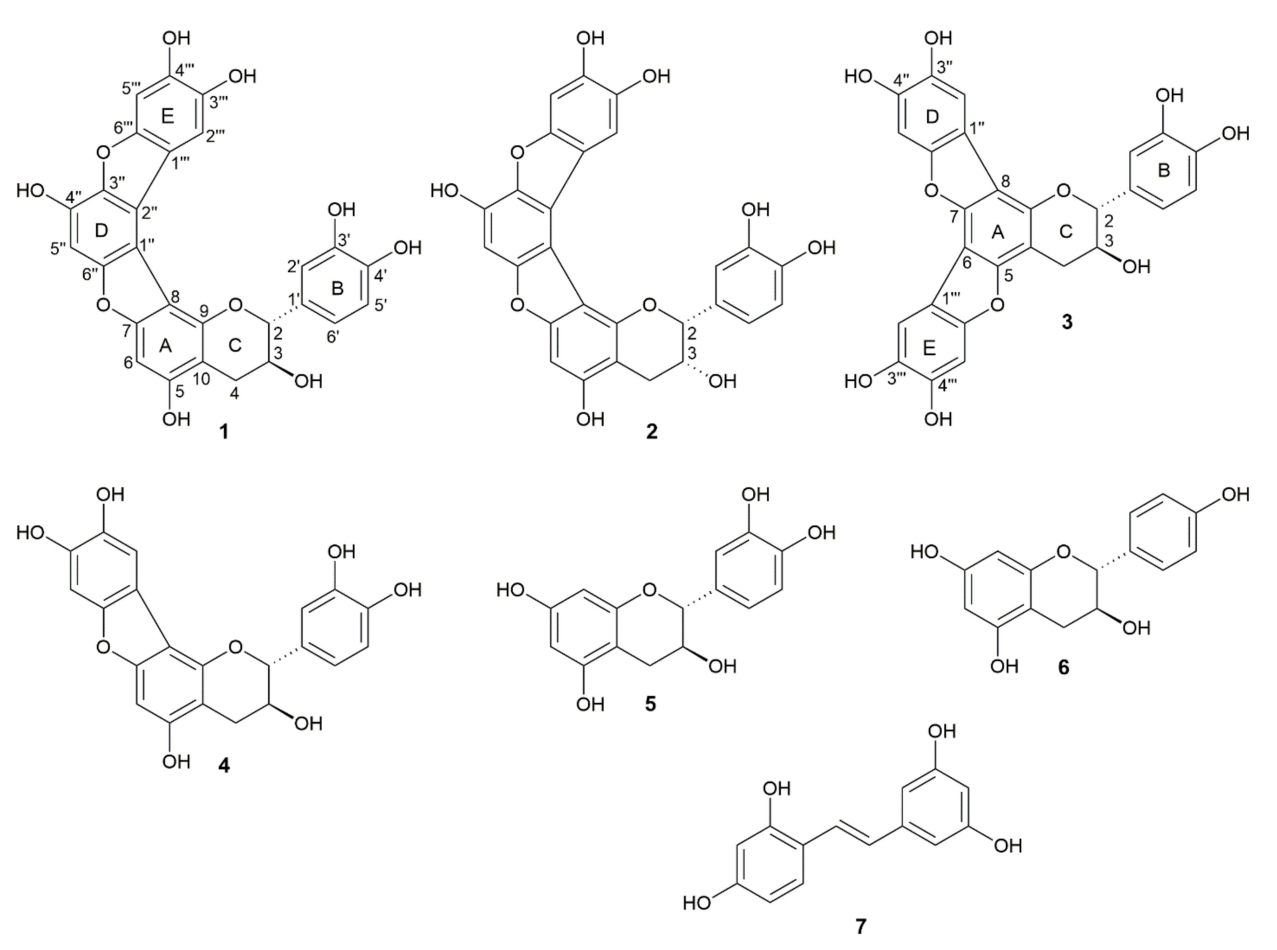
Structures of artocarpinol A (**1**), 3-*epi*-artocarpinol A (**2**), artocarpinol B (**3**), gambircatechol (**4**), (+)-catechin (**5**), (+)-afzelechin (**6**) and the oxyresveratrol (**7**) isolated from *A. lacucha*.

Chromatographic separation of the crude methanolic extract from leaves and stem extracts of *A. lacucha* yielded seven compounds (Figure 1). Compounds **1**–**3** have not yet been reported and now found in natural sources for the first time. All compounds were isolated and their chemical structures established, in particular using 1D and 2D NMR and MS spectra. Additionally, theoretical calculations using GAUSSIAN09 software were performed to reveal the most stable isomeric forms of compounds **1**–**3** (see Section 2.2). Except compound **7**, all described compounds possess a flavan-3-ol core structure, whereas compounds **1**–**4** feature further extensions with one or two benzofuran moieties attached to the A ring of the flavan core. Due to these structural features, we suggest to assign these compounds to flavan-benzofuran, which would be a novel subclass of flavanols. Radical scavenging activities were assessed from compounds **1**–**4** the (see Section 2.5 and Section 2.6), and from compound **1** the cytotoxic properties were determined (Section 2.7).

Compound **4** was previously described from the leaves of *Uncaria gambier* (W. Hunter) Roxb. (Rubiaceae) and was named gambircatechol [17]. Its presence in the non-related species *A. lacucha* suggests a diversification in biosynthetic pathways starting from catechin (Section 2.3). The additional isolated compounds **5**–**7** are known from many plant taxa and their potential radical scavenger activities were assessed several times [18,19]. These compounds may contribute together with **1**–**4** to the plant internal protection against oxidative stress caused by radical oxygen species (ROS). 

### 2.1. Structure Elucidation

Compound **1** was isolated from the leaves of *A. lacucha* and indicated a molecular ion [M − H]^−^ of *m*/*z* 501.0856 detected with HR-TOF-ESI-MS. This correlates to the molecular formula C_27_H_18_O_10_ with the calculated *m*/*z* 501.0822 for [M − H]^−^. From the NMR measurements, one methylene group, nine methine groups and 17 quaternary carbon atoms were found. Further 1D and 2D NMR measurements indicated a catechin core structure. Another moiety, which consists of two additional aromatic rings, is bound at position 8 in the A-ring. These aromatic rings are fused by furan rings between the aromatic rings A and D and between D and E, as shown in Figure 1.

**Figure 2:**
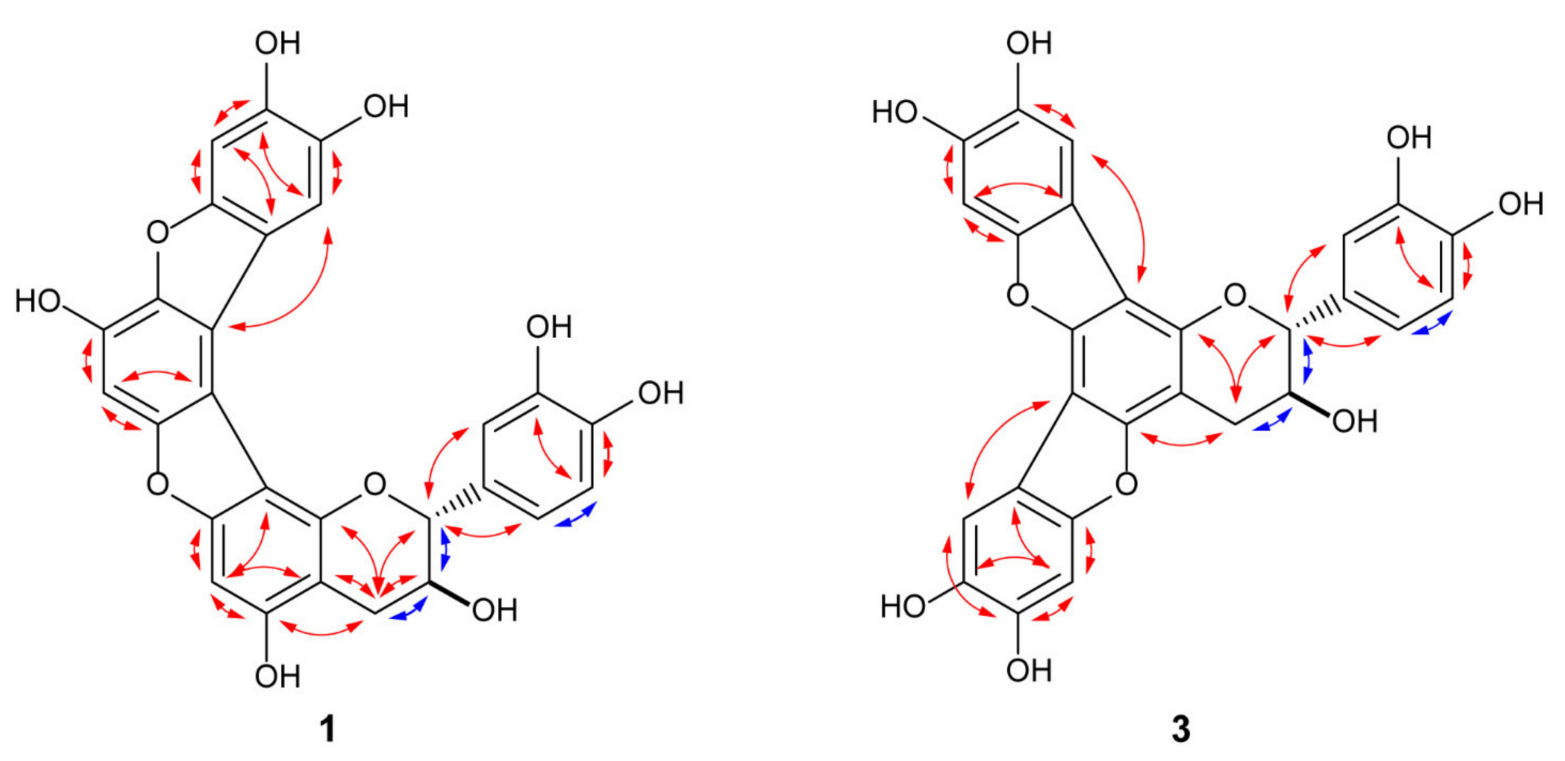
Important COSY (^1^H—^1^H) (blue) and HMBC (^1^H—^13^C) (red) correlations of artocarpinol A (**1**) and artocarpinol B (**3**).

The NMR chemical shifts, couplings and multiplicities of several nuclei in **1** are comparable to those of the structural closely related structure gambircatechol (**4**), which has also been isolated from *U. gambier* (compound **4**, see below) [17]. The core structure of catechin is assigned by ^1^H NMR data, which showed the chemical shifts of the A-ring at δH 6.63 ppm (H-6), B-ring at 6.82 ppm (H-2′), 6.73 ppm (H-6′) and 6.68 ppm (H-5′) and C-ring at 5.70 ppm (H-2), 4.51 ppm (H-3), 2.89 ppm (H-4a) and 2.68 ppm (H-4b). The chemical shifts of the carbon atoms in the catechin moiety (Table 1) also very well match those of the gambircatechol (**4**). An additionally singlet at δH 7.00 ppm in the ^1^H NMR points to a penta-substituted benzene ring (D), while the ^13^C NMR signals at δC 108.7, 119.7, 141.7, 143.6 and 153.8 ppm demonstrated two *sp*^2^ quaternary carbon and three *sp*^2^ oxygenated quaternary carbon, respectively, in this ring. The ^3^*J*_H–C_ coupling in HMBC between H-6 and C-8 together with H-5″ and C-1″ indicated the substituted C-C bond linkage between A-ring (C-8) and D-ring (C-1″), comparable to gambircatechol (**4**) [17]. In analogy to this, the ether bond linkage between C-7 in the A-ring and C-6″ in the D-ring is assigned by ^2^*J*_H–C_ coupling in HMBC between H-6 and C-7 and H-5″ and C-6″. Important COSY and HMBC correlations of **1** and also of **3** are given in Figure 2.

The additional NMR spectroscopic data indicated the presence of a fifth benzene ring (E) in the molecule, which is tetra-substituted. This benzene ring (E) is indicated by the chemical shifts and multiplicities of nuclei in positions 1‴ to 6‴. Two singlet signals at δH 8.45 ppm (H-2‴) and 7.00 ppm (H-5‴) and quaternary carbons at δC 152.8 ppm (C-6‴), 147.1 ppm (C-4‴), 142.5 ppm (C-3‴) and 117.7 ppm (C-1‴) indicate a tri-oxygenated benzene ring and one C-C bond connection. The ^2,3^*J*_H–C_ couplings between H-2‴ (8.45 ppm) and C-2″ (119.7 ppm), C-3‴ (142.5 ppm), C-4‴ (147.1 ppm) and C-6‴ (152.8 ppm) together with those between H-5‴ (7.00 ppm) and C-1‴ (117.7 ppm), C-4‴ (147.1 ppm) and C-6‴ (152.8 ppm) in HMBC indicate the *para*-oriented proton position of H-2‴ and H-5‴. They further display the C-C bond connection between C-2″ in D-ring and C-1‴ in E-ring, and ether bond connection between C-3″ and C-6‴. 

**Figure 3:**
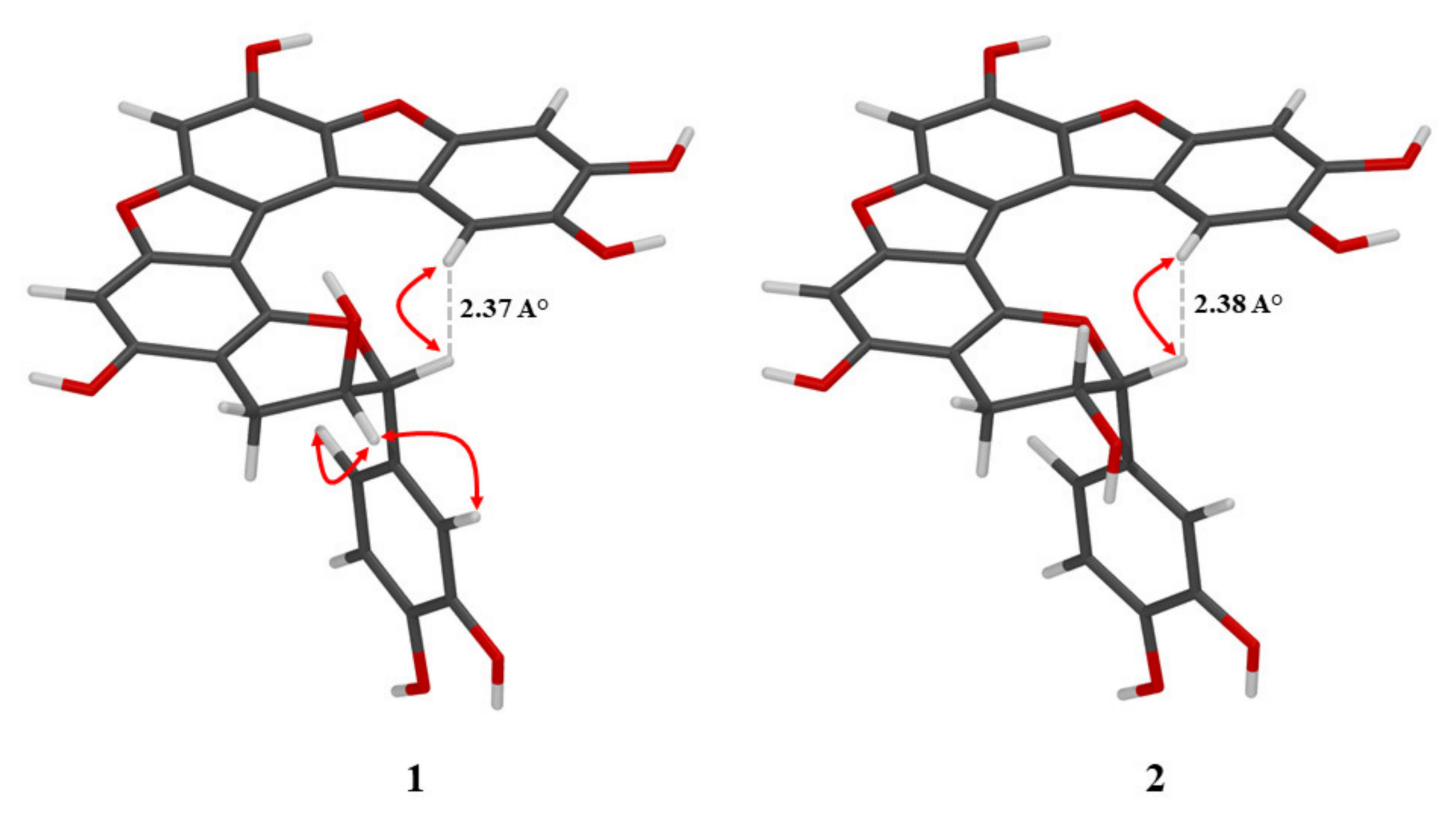
Three-dimensional structure simulation and key NOESY correlations (red arrows) of artocarpinol A (**1**) and 3-*epi*-artocarpinol A (**2**).

The relative configurations of positions 2 and 3 were deduced accordingly to the isolated (+)-catechin (**5**), which is a well-known compound from *A. lacucha*, and the structure calculation from Gaussian09 software (see Section 2.2). The detection of a cross peak between H-2 and H-2‴ in NOESY spectrum indicated a spatial closeness. In particular a weak nuclear Overhauser effect between H-2 and H-2‴ demonstrated that these two protons were in close proximity (2.37 Å) (Figure 3). Furthermore, the chiral center at position 3 is also deduced by a particular weak nuclear Overhauser effect between H-3 and H-6′. Based on these data, the *R*-configuration of position 2 and *S*-configuration of position 3 were assigned.

Three-dimensional structure simulation and important NOESY correlation of compound **1** are illustrated in Figure 3. All the NMR-spectroscopic data are depicted in Table 1 and the NMR spectra are shown in the Supplementary Materials (Figures S1–S7). According to the plant source, this compound was named artocarpinol A (**1**).

Compound **2** showed a molecular ion in HR-TOF-ESI-MS at *m*/*z* = 501.0845 [M − H]^−^, which corresponds to the molecular formula C_27_H_17_O_10_ (calcd *m*/*z* = 501.0822 [M − H]^−^). By using NMR, one methylene group, nine methine groups and seventeen quaternary carbon atoms were found. The results from 1D and 2D NMR measurement strongly corresponded to artocarpinol A (**1**). Therefore, the structure of compound **2** also consists of a catechin core structure and two more aromatic moieties fused by a furan ring. The overall ^1^H NMR data of compound **2** are similar with those of compound **1**, nevertheless the low-field shifted peak of H-2′ (δH 7.01 ppm), H-6′, (δH 6.87 ppm) and H-4a (δH 3.11 ppm) in a catechin core structure were found. This result indicated a different configuration at position 3 and suggests *epi*-catechin as its core structure moiety. Moreover, a lack of detectable nuclear Overhauser effect between H-3 and H-2′ and H-6′ in the NOESY indicates that these protons are not in close proximity, which can be assigned that proton H-3 and ring A are not both is in quasi equatorial position. On the other hand, the relative configuration of position 2 is indicated by a significant NOESY cross peak between H-2 and H-2‴ (Figure 3).

The ether bond (C-7 and C-6″) and C-C bond (C-8 and C-1″) connection between A-ring and D-ring are confirmed by ^2,3^*J*_H–C_ coupling between H-6 (δH 6.65 ppm), C-7 (δC 158.2 ppm) and C-8 (δC 17.4 ppm) together with H-5″ (δH 7.00 ppm), C-6″ (δC 153.8 ppm) and C-1″ (δC 18.6 ppm) in HMBC. In addition, ^2,3^*J*_H–C_ coupling in HMBC between H-2‴ (δH 8.34 ppm) and C-2″ (δC 119.7 ppm) and H-5‴ (δH 6.97 ppm), C-1‴ (δC 117.5 ppm) and C-6‴ (δC 152.5 ppm) indicated the C-C bond connection between C-1‴ in E-ring and C-2″ in D-ring and ether bond connection between C-6‴ in E-ring and C-3″ in D-ring. The structure of compound **2** is displayed in Figure 1 and it is named 3-*epi*-artocarpinol A (**2**) (C_27_H_18_O_10_). Additionally, Figure 3 illustrates the three-dimensional structure simulation and key NOESY correlation of compound **2**. The NMR-spectroscopic data is provided in Table 1 and also, 1D and 2D NMR spectra are presented in the Supplementary Materials (Figures S9–S15).

Compound **3** also showed a molecular ion in HR-TOF-ESI-MS at *m*/*z* = 501.0835 [M − H]^−^, which corresponds to a molecular formula of C_27_H_17_O_10_ (calcd *m*/*z* = 501.0822 [M − H]^−^). The chemical shifts and multiplicities of nuclei again indicated the signals of one methylene group, nine methine groups and seventeen quaternary carbon atoms. Further interpretation of 1D and 2D NMR spectroscopy suggests the presence of a catechin core structure together with two additional aromatic moieties fused by furan ring. According to the ^1^H NMR and ^13^C NMR data of the structural similar compound gambircatechol (**4**), the occurrence of A-ring in catechin core structure was confirmed by two dimensional heteronuclear NMR experiments. The existence of C-5 (δC 155.3 ppm), C-9 (δC 148.4 ppm) and C-10 (δC 100.5 ppm) can be confirmed by ^2,3^*J*_H–C_ coupling in HMBC between H-4 (δH 3.34 and 3.05 ppm) and these carbons. Two protons showing singlets at δH 7.47 ppm (H-2″) and δH 7.05 ppm (H-5″) together with four quaternary carbons at δC 115.1 ppm (C-1″), δC 143.5 ppm (C-3″), δC 146.0 ppm (C-4″) and δC 151.7 ppm (C-6″) indicated of tetra-substituted benzene containing the trioxygenated group. In addition, ^2,3^*J*_H–C_ coupling between H-2″ (δH 7.47 ppm) and C-8 (δC 104.6 ppm) along with H-5″ (δH 7.05 ppm) and C-1″ (δC 115.1 ppm) and C-6″ (δC 151.7 ppm) in HMBC indicated the *para*-oriented position of these two proton and C-C bond (C-1″ and C-8) and ether bond (C-6″ and C-7) linkages between A-ring and D-ring. 

The existence of the E-ring was demonstrated by the chemical shifts and multiplicities of nuclei of two singlet protons at δH 7.38 ppm (H-2‴) and δH 7.09 ppm (H-5‴) additional with four quaternary carbons at δC 116.3 ppm (C-1‴), δC 143.2 ppm (C-3‴), δC 145.7 ppm (C-4‴) and δC 151.5 ppm (C-6‴). The C-C bond connection between C-6 in A-ring and C-1‴ in E-ring was confirmed by ^3^*J*_H–C_ coupling between H-2‴ and C-6 in HMBC. Moreover, the ether bond linkage between C-5 in A-ring and C-6‴ in E-ring also confirmed by ^2,3^*J*_H–C_ coupling between H-5‴ and C-6‴, and H-4 and C-5 in HMBC.

The relative configuration of positions 2 and 3 are deduced accordingly to the well-known compound (+)-catechin (**5**). In addition, a weak nuclear Overhauser effect between H-3 and H-2′ indicated a close proximity between these two protons. On the other hand, the lacking of NOESY correlation between H-2 and H-3 indicates that these two protons are not spatially close, which suggests that they are not located at the same side of the C-ring. A comprehensive description of the relative and absolute stereochemistry at positions 2 and 3 for compounds **1**–**3** is carried out in Section 2.2. This yet undescribed catechin derivative is named artocarpinol B (**3**) (C_27_H_18_O_10_). The NMR-spectroscopic data is shown in Table 1 and also 1D and 2D NMR spectra are provided in the Supplementary Materials (Figures S17–S23).

In addition to the above described compounds, the three flavan-3-ol derivatives gambircatechol (**4**), (+)-catechin (**5**) and (+)-afzelechin (**6**) could also be isolated and identified from the leaf extract. The stembark extract yielded the stilbene oxyresveratrol (**7**) in higher quantities but was not detectable in the leaf extract by HPLC-UV-PDA. The structures of compounds **4**–**7** were elucidated by 1D and 2D NMR spectroscopy and HR-TOF-ESI-MS mass spectrometry. The ^1^H, ^13^C NMR and mass spectra are provided in the Supplementary Materials (Figures S25–S40). The structure elucidation of these compounds was proven by comparison of the corresponding spectroscopic and spectrometric data with previous reports [13,17,20]. 

The presence of the stilbenoid oxyresveratrol (**7**) in the stem bark deserves special attention due to its ascribed role as phytoalexin [11]. However, the accumulated amount present in the stembark extract (67 mg isolated from 8.5 g ethyl acetate extract) and also in the heartwood of *A. lacucha* [14] may contradict its role as phytoalexin in this plant species. Since other stilbenoid derivatives have already been found in this plant species [7], these compounds may be present constitutively.

Interestingly, none of the prenylated compounds could be detected during this work, although such compounds have already been described from this species. A comprehensive literature survey revealed that most of such compounds were identified from the roots or root bark [8,10,21] and only a few derivatives were isolated from the aerial parts like twigs [7]. Nevertheless, the presence of compounds featuring prenyl-/geranyl side chains in the examined extracts could not be excluded, since the analyses by HPLC-PDA resulted in chromatograms showing a bulk of inseparable compounds, all of them possess identical UV spectra to compounds **5** and **6**.

### 2.2. Stereochemistry of ***1***–***3***

The relative configuration of compounds **1**–**3** were also deduced from ^1^H NMR spectra NOESY spectra and in particular by structure simulation using Gaussian09 software. Therefore the density functional theory (DFT) with six d-type Cartesian−Gaussian polarization functions (6-31G(d,p)) in Gaussian09 was used to calculate the energy-minimized conformer of these compounds. Each simulation structure was compared with the observed correlations in NOESY as shown in Figure 3. Moreover, their optical rotation (OR) was calculated by using the density functional theory (DFT) based on the “self-consistent field” method (SCF). Comparing the observed optical rotation values and the calculated optical rotation of compound **1** −310.00 (calcd = −355.81), **2** −222.00 (calcd = −275.36) and **3** −170.00 (−176.10), the results were close significantly. In addition, the estimated coupling constants from Karplus equation were used to explain the dihedral angle affected between the two protons at C-2 and C-3. The data from theoretical calculation and structure simulation were compared to the NMR results.

Compound **1** indicates *R*-configuration of position 2 and *S*-configuration of position 3, which correspond to the results from theoretical calculation and NMR spectroscopy. The ^4^*J*_H–H_ long range coupling (W coupling) between H-2 at δH 5.70 ppm (d, *J* = 3.1 Hz) and H-4a at δH 2.89 ppm (ddd, *J* = 16.8, 3.7, 1.7 Hz) implied that these two protons were located in the same plane. Moreover, the angle between H-3 and H-4a (*Φ* = 69.9°), compared to H-3 and H-4b (*Φ* = −46.8°), corresponded to the calculated coupling constant obtained from the Karplus equation. This was in accordance to the higher coupling constant value of H-4b at δH 2.68 ppm (dd, *J* = 16.7, 4.3 Hz).

Compound **2** demonstrates *R*-configuration at positions 2 and 3. The protons in position 2 at δH 5.65 ppm (d, *J* = 3.3 Hz), and in position 3 at δH 4.47 ppm (ddd, *J* = 8.1, 5.0, 3.3 Hz) and in position 4a, δH 3.11 ppm (ddd, *J* = 15.9, 5.0, 1.2 Hz) were in the same plane, which was not confirmed alone by the coupling between these three protons. This is also indicated by the lower coupling constant value of proton position 4a (*Φ* = −53.3°, ddd, *J* = 15.9, 5.0, 1.2 Hz) compared to the proton at position 4b (*Φ* = −169.7°, dd, 15.9, 7.7 Hz). The simulation structures of **1** and **2** are displayed in Figure 3. 

Compound **3** indicates *R*-configuration at position 2 and *S*-configuration at position 3, which were the same with compound **1**, however, the conformation at C-ring was different. According to the Karplus equation, the larger coupling constant of proton position 2 in compound **3** (*Φ* = 178.1°, d, *J* = 7.6 Hz) compared to compound **1** (*Φ* = 68.6°, d, *J* = 3.1 Hz) and compound **2** (*Φ* = 52.3°, d, *J* = 3.3 Hz) confirms the relative dihedral angle between H-2 and H-3 from the structure simulation results. Moreover, the larger angle between H-3 and H-4b (axial, *Φ* = 164.8°) compared to the angle between H-3 and H-4a (equatorial, *Φ* = 48.9°) corresponded to the higher coupling constant value of H-4b (dd, *J* = 15.8, 8.2 Hz) rather than H-4a (dd, *J* = 15.8, 5.3 Hz).

The 2*R*,3*S*-configuration of artocarpinol A (**1**) and B (**3**) was also present in compounds **4**, **5** and **6**. With regard to the substitution pattern in ring B, it seems likely that (+)-catechin (**5**) is a biosynthetic precursor of gambircatechol (**4**), from which compounds **1** and **3** are then subsequently formed. In contrast, 3-*epi*-artocarpinol A (**2**) has a 2*R*,3*R*-configuration, which indicates that 3-*epi*-catechin is likely its biosynthetic precursor.

### 2.3. Proposed Biosynthesis of ***1***–***3***

A possible biosynthesis of artocarpinol A (**1**) and artocarpinol B (**3**) starts from (+)-catechin (**5**) and comes across gambircatechol (**4**) as an intermediate. Biosynthesis of 3-*epi*-artocarpinol A (**2**), which starts from the structure of 3-*epi*-catechin and contains this as a central moiety, is quite likely very similar. A C-C bond formation is the key reaction step in this biosynthetic reaction cascade. Comparable C-C bond formations are key steps in further diversification of flavanoids and related or similar aromatic natural products. Examples are flavanoid prenylation [22,23] and the biosynthesis of flavanoid alkaloids [24] and flavonoid C-glycosides [25], which are based on well-studied enzyme-catalyzed reactions.

**Scheme 1:**
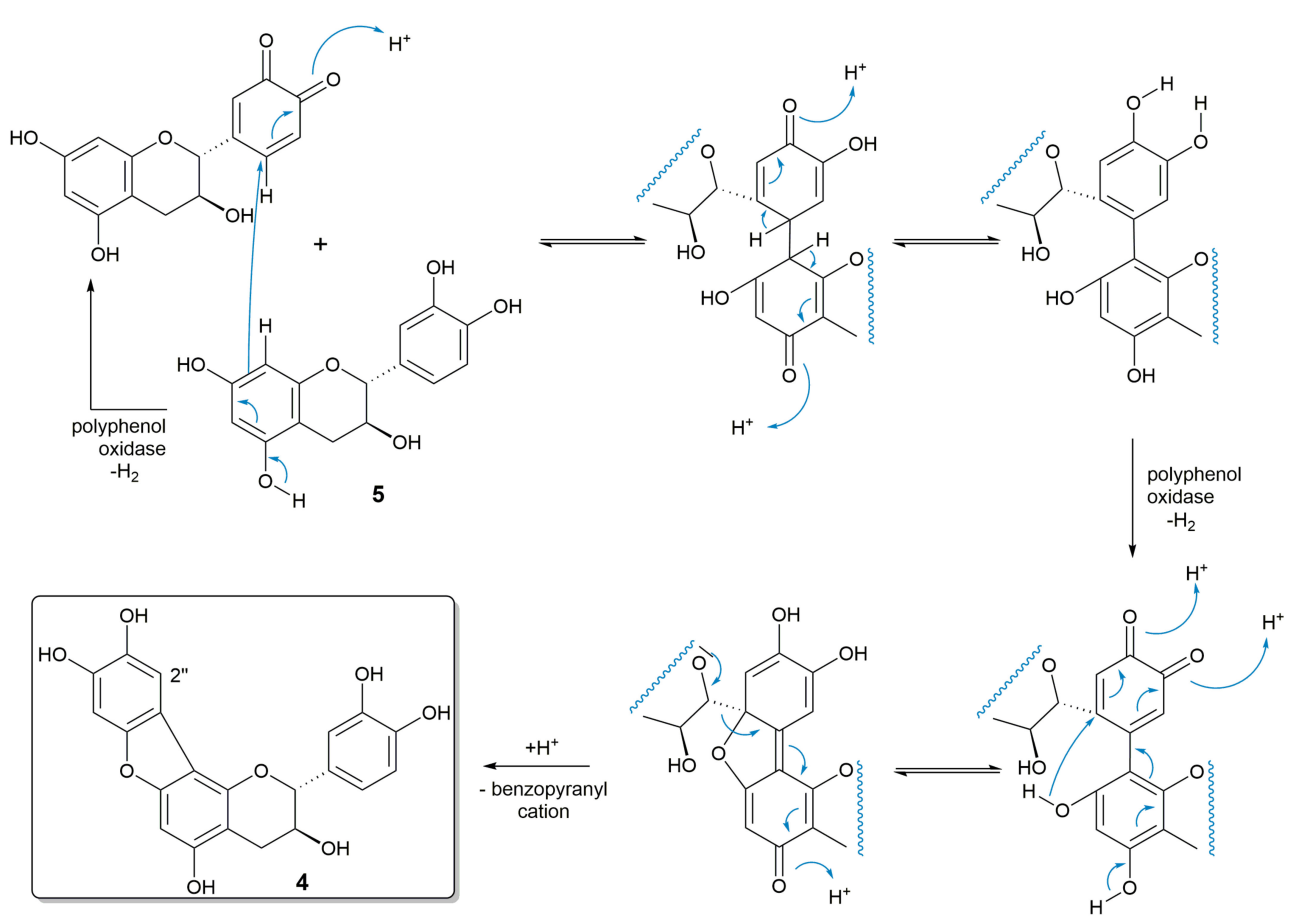
Possible biosynthetic pathway starting from (+)-catechin (**5**) and leading to gambircatechol (**4**) according to Guyot et al. [26] and Janse van Rensburg et al. [27]. Since the enzymes involved in *A. lacucha* are not yet discovered, they are referred as “polyphenol oxidase” in general. The mechanism for the biosynthesis of artocarpinols starting from gambircatechol (**4**) likely proceeds in accordance to this proposed mechanism. This reaction cascade starts from position 2″ in 4 with an attack to an oxidized form of (+)-catechin.

The dimerization of flavanoids is furthermore attributed to polyphenol oxidase-catalyzed oxidation of (+)-catechin (**5**) to the according *ortho*-quinone and a subsequent Michael-type addition to a second (+)-catechin (**5**) [26]. The resulting non-symmetrical dimer is described to be the starting point of a further tyrosinase-catalyzed oxidation, which leads to the formation of the furan ring and splitting off a benzopyranyl group [27]. In this reaction cascade again an oxidation to an *ortho*-quinone is formulated, which is followed by spontaneous intramolecular oxycyclization, loss of the benzopyranyl moiety and rearomatization. According to Janse van Rensburg et al. [27] this reaction leads directly to the structure of gambircatechol (**4**), which was a few years later isolated as a natural product for the first time [17]. A comparable reaction sequence starting from gambircatechol (**4**) and (+)-catechin (**5**) can further lead to artocarpinol A (**1**) and artocarpinol B (**3**). The biosynthesis of 3-*epi*-artocarpinol A (**2**) is likely based on the same mechanism that starts with 3-*epi*-catechin. The proposed reaction sequence is shown schematically in Scheme 1 for the example of the gambircatechol biosynthesis.

### 2.4. Oxidation of ***1***–***4*** to Ortho-Quinone Structures 

The easy oxidizability of polyphenols to *ortho*-quinones, which was already described in the possible biosynthesis [26,27], can also be observed for the isolated compounds **1**–**4**. In addition to the molecular ions of the polyphenol structures, all HR-ESI-MS spectra show signals of ions with a mass, which indicate the loss of two hydrogen atoms. These oxidized analogues appeared in different amounts (Figures S8, S16, S24, S32, S35 and S38); in particular the peak of the *ortho*-quinone form of **4** was the dominant signal resulting from an isolation of gambircatechol (**4**) (Figure S35). However, only the reduced form of gambircatechol was detected in the subsequently performed NMR measurement (Figures S33 and S34). It was therefore not yet possible to determine exactly whether the oxidation and reduction preferentially occur at positions 3″ and 4″ in the D-ring or at positions 3′ and 4′ in the B-ring. The subsequent reactions to the artocarpinols (**1**–**3**) make the positions in the D-ring quite likely. Due to the occurrence of the *ortho*-quinone in (+)-catechin (**5**) (Figure S38), oxidation of the B-ring however cannot be excluded. The same argumentation can be made for the artocarpinols (**1**–**3**), which allow the formation of *ortho*-quinone forms either in the E-ring (positions 3‴ and 4‴) or in the B-ring (positions 3′ and 4′).

Such relative good oxidizability of compounds **1**–**5** as well as the quite good reducibility of their *ortho*-quinone analogues indicate that **1**–**5** can contribute to the defense of *A. lacucha* against predators by irritating the oral area of herbivores [28]. Furthermore, it is interesting to examine the radical scavenger activities, in particular of compounds **1**–**3**, in some more detail.

### 2.5. Radical Scavenging Activities of ***1***–***4***

The DPPH method (2,2-diphenyl-1-picrylhydrazyl) was applied to investigate the radical scavenging activity of isolated compound **1**–**4** [29,30]. To evaluate the EC_50_ values, the UV absorption at 517 nm of each compound was recorded after 30 min and compared to ascorbic acid and α-tocopherol. Compound **1** (EC_50_ = 9.4 ± 1.0 µM), **2** (EC_50_ = 12.2 ± 1.1 µM), **3** (EC_50_ = 10.0 ± 1.5 µM) and **4** (EC_50_ = 19.0 ± 2.6 µM) exhibited higher radical scavenger activity compared to both ascorbic acid (EC_50_ = 34.9 µM) and α-tocopherol (EC_50_ = 48.6 µM). This potent effect is not surprising since compounds **1**–**4** display hydroxy groups in positions 3′ and 4′ and in positions 3‴ and 4‴, which may, depending on the environment, easily form *ortho*-quinones as mentioned in Section 2.4. The formation of comparable *ortho*-quinones in presence of DPPH has already been described [31].

### 2.6. Antioxidative Effects of Artocarpinol A (***1***) in Cancer Cells

**Figure 4:**
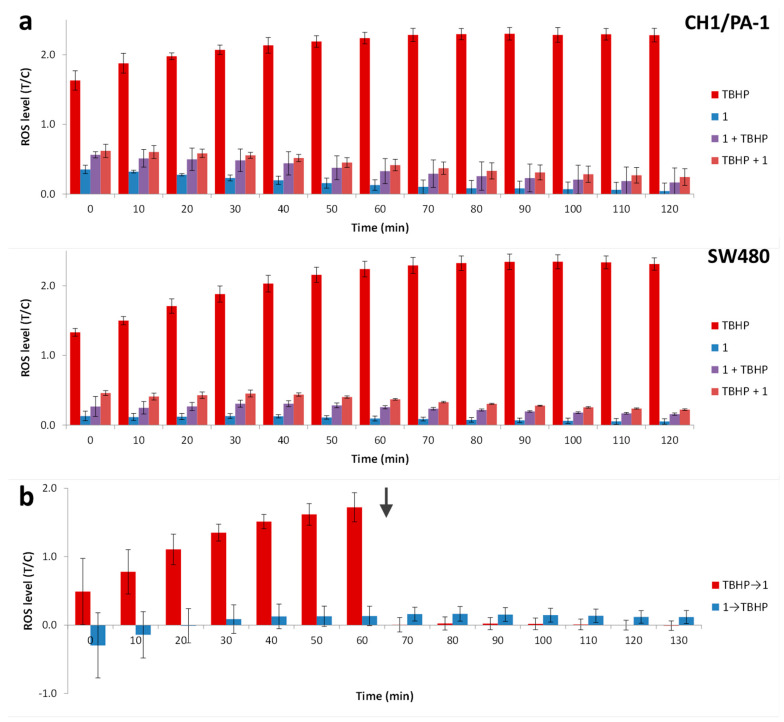
Cellular ROS levels upon treatment with **1** and/or TBHP, monitored by the DCFH-DA assay: (**a**) CH1/PA-1 and SW 480. cells exposed for 2 h to single compounds or simultaneous combinations and (**b**) SW480 cells exposed for 1 h per compound to consecutive combinations (arrows indicate the time point of compound exchange). Values are means ± standard deviations from at least two independent experiments. Values of 0 and 1 correspond to blanks (medium only) and untreated controls, respectively, for each time point.

To figure out whether the radical-scavenging properties of compound **1** might be biologically relevant in cancer cells, its effects on ROS levels were studied by means of the fluorimetric 2′,7′-dichlorodihydrofluorescein diacetate (DCFH-DA) assay [32] in various in vitro settings. When applied alone at a concentration of 20 µM, tremendous antioxidative effects developed within the first 2 h, with average ROS levels decreasing from 35% to 4% and from 13% to 5% in CH1/PA-1 and SW480 cells, respectively, relative to untreated controls (100%), whereas the strong oxidant *tert*-butylhydroperoxide (TBHP; commonly used as a positive control for ROS generation), raises these levels to 230% at the same concentration within the same time period. When the two compounds were added immediately after each other and cells exposed to the equimolar simultaneous combination for 2 h, **1** completely abolished the oxidative effects of TBHP (even reducing the ROS levels to less than 25% of untreated controls towards the end of the 2 h test period in both cell lines), no matter which of the compounds was added first (Figure 4a). When the two compounds were applied consecutively for 1 h each (thereby minimizing their extracellular interaction), SW480 cells treated with TBHP first were relieved of oxidative stress as soon as compound **1** was added, whereas cells pretreated with **1** effectively resisted an oxidative challenge by TBHP (Figure 4b). We hence conclude that artocarpinol A (**1**) is a very potent antioxidative agent even in the cellular environment.

### 2.7. Cytotoxic Properties of Artocarpinol A (***1***)

**Figure 5:**
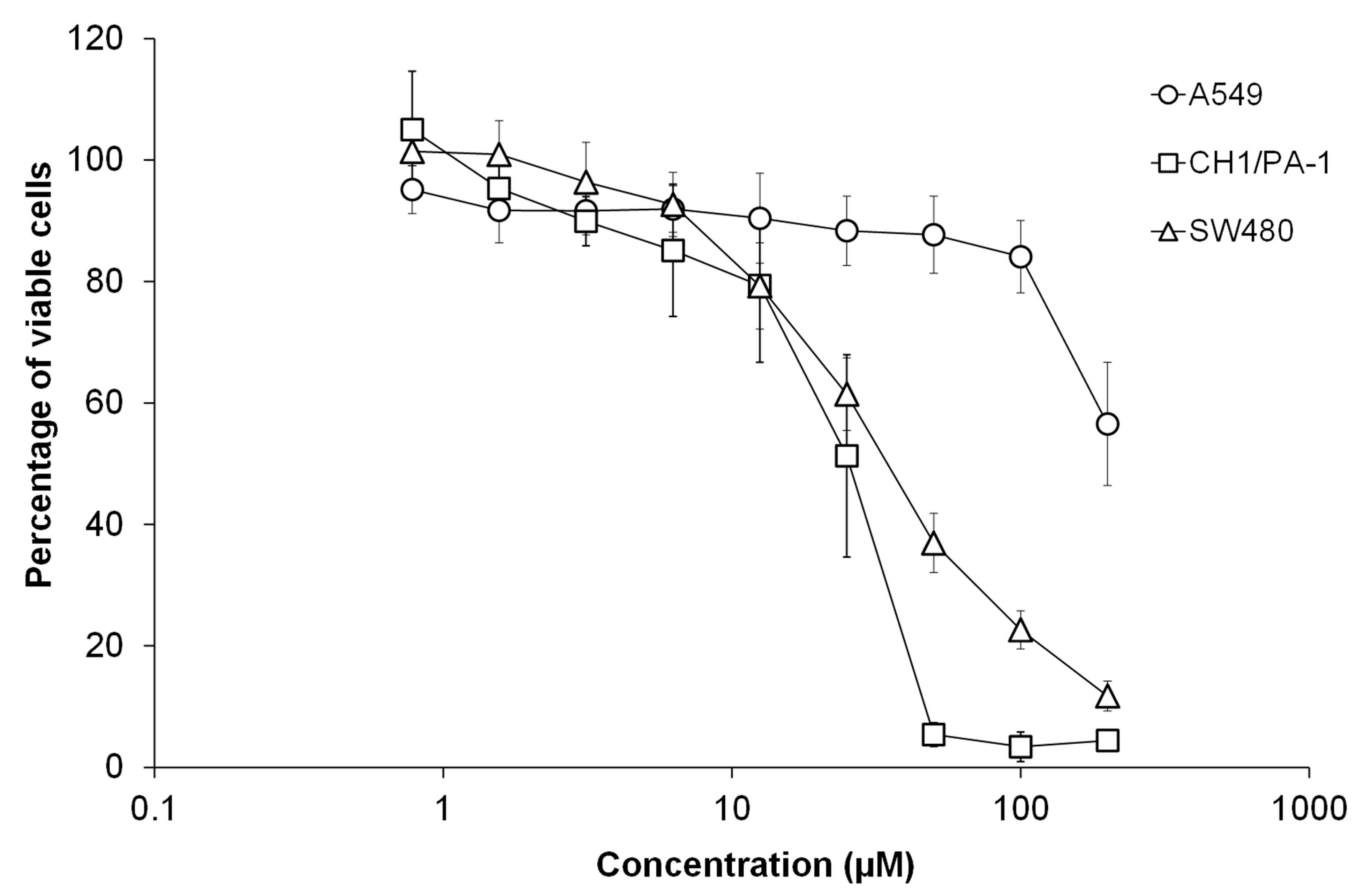
Concentration–effect curves of compound **1** in three human cancer cell lines, obtained by the MTT assay with 96 h exposure (means ± standard deviations from three independent experiments).

Representative for compounds **1**–**3**, artocarpinol A (**1**) was investigated for its capacity to inhibit the proliferation of cancer cells in vitro by means of the MTT assay, a colorimetric microculture test, in three human carcinoma cell lines exposed to the compound for 96 h. Concentration–effect curves (Figure 5) revealed a moderate, but consistent concentration-dependent activity in two of the three cell lines employed, with IC_50_ values of 25 ± 6 µM in the broadly chemosensitive ovarian teratocarcinoma cell line CH1/PA-1 and 34 ± 4 µM in the P-glycoprotein-expressing colon carcinoma cell line SW480. Only in the highly multidrug-resistant non-small cell lung cancer cell line A549, its activity proved insufficient to reach an average IC_50_ within a concentration range of up to 200 µM.

## 3. Experimental

### 3.1. Plant Material and Extraction Procedure

The plant material was collected in Udon Thani, Thailand in January 2020 (17°08′03.7″ N 102°37′02.5″ E). A voucher specimen (BK No. 070908) has been deposited at the Bangkok Herbarium, Plant Varieties Protection Office, Department of Agriculture, Bangkok, Thailand.

Leaves—Air-dried and ground leaves of *A. lacucha* (1.8 kg) were extracted with methanol at room temperature (3 × 7 days). The filtered methanolic extracts were pooled and evaporated by using a rotary evaporator to yield a dry residue (162.6 g). The crude extract was partitioned between distilled water, petroleum ether (PE), chloroform (8.1 g) and ethyl acetate (EtOAc) (13.3 g), respectively.

Stembark—The air-dried and ground stembark of *A. lacucha* (4.0 kg) was sequentially treated with hexane, dichloromethane, ethyl acetate and MeOH (3 × 7 days for each solvent). All extracts were evaporated by using a rotary evaporator to afford dry residue of hexane (11.9 g), dichloromethane (6.4 g), ethyl acetate (8.5 g) and MeOH (212.6 g), respectively.

### 3.2. General Experimental Procedures

HPLC analyses were performed on Agilent 1100 series (Agilent, Vienna, Austria) with UV–diode array detection using a Hypersil BDS-C18 column, 250 mm × 4.6 mm, 5 μm particle size, at a flow rate of 1.0 mL min^−1^ and an injection volume of 10 µL. The concentration of the injected crude extracts was set after evaporation of the extraction solvent at 10 mg mL^−1^ in pure methanol (MeOH; VWR, Vienna, Austria). An aqueous solution containing 10 mM ammonium acetate (A; VWR, Vienna, Austria) and MeOH (B) were used as eluents. The following gradient was applied: From 20 to 90% B in A within 15 min, from 90–100% B in A within 0.1 min and 100% B was kept for 5.9 min. The wavelength of detection was set at 230 nm (reference WL 360 nm). MPLC separations were done over a silica gel 60 column (40–63 µm particle size), eluted with mixtures of petrol ether (PE), ethyl acetate (EtOAc) and MeOH. TLC analyses were done on silica gel 60 F_254_ plates, layer thickness 0.2 mm (Merck, Darmstadt, Germany) developed with CHCl_3_/MeOH 90:10 and 80:20. Final purification was done via CC on Sephadex LH-20 (GE Healthcare) eluted with methanol. All the preparative separation procedures were monitored by HPLC and TLC.

For NMR spectroscopic measurements each compound was dissolved in deuterated solvent (CD_3_OD, DMSO-*d_6_*; Eurisotop, Saarbrücken, Germany) (the isolated amounts of 1–5 mg in 0.6 mL) and transferred into 5 mm high precision NMR sample tubes. NMR spectra were recorded on a Bruker AVIII 600 spectrometer at 600.25 MHz (^1^H) and 150.93 MHz (^13^C) at the Department of Organic Chemistry, University of Vienna and Bruker 400 MHz AVANCE III HD spectrometer at 400 MHz (^1^H) and 100 MHz (^13^C) at the Department of Chemistry, Faculty of Science, Kasetsart University, Bangkok, Thailand. Spectra were processed with MestReNova 14.1.2 software. Chemical shifts (δ) are given in ppm; for ^1^H relative to residual non-deuterated solvent signals in methanol (δ_H_ = 3.31 ppm) and DMSO-*d*_6_ (δ_H_ = 2.50 ppm) and for ^13^C relative to solvent signals (CD_3_OD, δ_C_ = 49.0 ppm; DMSO-*d*_6_, δ_C_ = 39.5 ppm). CH_3_, CH_2_, CH and C_q_ are indicated by the multiplicities (q, t, d and s), respectively, which indicate the signal form, as if the ^13^C NMR measurements had been taken without proton broadband decoupling. 

The optical rotations (OR) of compounds **1**–**3** were measured by the sodium D line using a 100 mm of path length cell on a Perkin Elmer Automatic Polarimeter 341 (Perkin Elmer, Rodgau, Germany). The concentration of compound **1**–**3** was each set at 0.25 mg mL^−1^ in pure methanol. 

HR-ESI-MS spectra were obtained on a maXis UHR ESI-Qq-TOF mass spectrometer (Bruker Daltonics, Bremen, Germany). Samples were dissolved and further diluted in ACN/MeOH/H_2_O in the ratio of 99:99:2 (*v*/*v*/*v*) and directly infused into the ESI source with a syringe pump. The ESI ion source was operated as follows: capillary voltage: 4.0–4.5 kV, nebulizer: 0.4 bar (N_2_), dry gas flow: 4 L min^−1^ (N_2_) and dry temperature: 180 °C. Mass spectra were recorded in the range of *m*/*z* 50–1900 in the positive and negative ion mode. The sum formulae of the detected ions were determined using Bruker Compass DataAnalysis 4.1 based on the mass accuracy (Δ*m*/*z* ≤ 5 ppm) and isotopic pattern matching (SmartFormula algorithm).

Initially, the Gaussian09 software package was used for energy optimization and frequency calculations of a single molecule. The DFT method was carried out by hybrid function Becke−3−Lee−Yang−Parr (B3LYP) and double-ζ polarized basis set with six d-type Cartesian−Gaussian polarization functions (6-31G(d,p)). The DFT calculations were used for observation energy minima and vibration mode of a single molecule. The optical rotation (OR) values were also calculated by DFT and “self-consistent field” method (SCF).

IR spectra were recorded on a Bruker Tensor 37 FT-IR spectrometer with Bruker Platinum ATR (Diamant); resolution: 4 cm^−1^, number of scans: 64.

### 3.3. Isolation Procedure

Leaves—The ethyl acetate fraction was chromatographed over MPLC using mixtures consisting of petrol ether, ethyl acetate and methanol, starting with 20% EtOAc in petrol ether to 100% MeOH (*v*/*v*) with the compositions 80/20/0; 70/30/0; 60/40/0; 50/50/0; 30/70/0; 10/90/0; 0/100/0; 0/90/10; 0/80/20; 0/70/30; 0/50/50 and 0/0/100 (100–200 mL each). This separation afforded 30 fractions between 20 and 300 mL named as M01–30. The pooled fractions M05 and M06 (680 mg) were further chromatographed over Sephadex LH20 eluted with MeOH. This step yielded 13.4 mg of impure **6**. Repetition of this step yielded 3.1 mg of **6**. Separation of the combined fractions M10 and M11 (439 mg) on Sephadex LH20/MeOH yielded 22 fractions, of 10 mL each, yielded 11.5 mg of **1** and 1.5 mg of **3** (8.3 × 10^−5^%) together with 130.3 mg of impure **2**. Repeated CC over Sephadex LH20/MeOH afforded 1.1 mg of **2** (6.1 × 10^−5^%. Purification of fraction M08 (67.2 mg) via Sephadex LH20/MeOH gave 5 mg of **5** (2.8 × 10^−4^%) and further 2 mg (1.1 × 10^−4^%) of **4**. Fraction M07 (420 mg) was chromatographed over 20 g silica gel 60, (40–60 µm) using *n*-heptane/ethyl acetate/MeOH mixtures in the compositions 90/10/0; 80/20/0; 70/30/0; 50/50/0; 30/70/0; 0/100/0; 0/80/20; 0/50/50 and 0/0/100 (100 mL each; *v*/*v*/*v*). This step afforded 18 fractions á 50 mL. Fractions 9 and 10 were pooled (327 mg) and subjected to CC over Sephadex LH20/MeOH, followed prep. TLC developed in CHCl_3_/MeOH 80:20. The broadest band was scraped off and the extraction was accomplished with MeOH. The final purification was achieved by over Sephadex LH20/MeOH. This yielded 1.3 mg of an *ortho*-quinone of **4**. 

Stembark—The ethyl acetate fraction was fractionated over silica gel column chromatography using mixtures of hexane, ethyl acetate and methanol, starting with 35% ethyl acetate in hexane to 20% MeOH (*v*/*v*/*v*) in the compositions 65/35/0, 55/45/0, 45/55/0, 35/65/0, 35/75/0, 15/85/0, 5/95/0, 0/95/5 and 0/80/20. This separation step afforded ten fractions between 20 and 300 mL named as C01–10. The selected fraction C06 (284 mg) was further chromatographed over silica gel column chromatography with 60% ethyl acetate in *n*-hexane. This step yielded 67.0mg of oxyresveratrol (**7**).

### 3.4. DPPH Assay

From compounds **1**–**4** stock solutions in MeOH with concentrations of 59 µg mL^−1^ for compound **1**, 80 µg mL^−1^ (**2**), 100 µg mL^−1^ (**3**) and 100 µg mL^−1^ (**4**) were prepared. From these stock solutions dilution series in microwell plates reaching conc. ranges of 117.5 µmol L^−1^ to 57.3 nmol L^−1^ for **1**, 199.1 µmol L^−1^ to 97.2 nmol L^−1^ (**2**), 159.3 µmol L^−1^ to 77.7 nmol L^−1^ (**3**) and 252.4 µmol L^−1^ to 123.2 nmol L^−1^ (**4**) were prepared by transferring 50 µL of the stock solution into the first well and diluting it with the equal volume of MeOH. After that, 50 µL of freshly prepared DPPH solution at a concentration of 200 µM was added. After 30 min the UV extinctions were measured at 517 nm (free radical DPPH) and 700 nm (reference) using a Tecan Sunrise plate reader. The EC_50_ values were calculated using the online tool from www.ic50.tk (accessed on 25 January 2021). The potent antioxidants ascorbic acid and α-tocopherol were used for comparison.

### 3.5. Cytotoxicity Assay

For biological experiments, a 40 mM stock solution of compound **1** was prepared in DMSO (Fisher Scientific, Waltham, MA, USA) and stored at 4–8 °C. All cell culture media, supplements and reagents were purchased from Sigma-Aldrich (St. Louis, MO, USA) unless stated otherwise, all plasticware from Starlab (Hamburg, Germany), and all incubations were at 37 °C under a moist atmosphere containing 5% CO_2_ in air. CH1/PA-1 ovarian teratocarcinoma (kindly provided as CH1 by Lloyd R. Kelland, CRC Centre for Cancer Therapeutics, Institute of Cancer Research, Sutton, UK; later identified as PA-1 by STR profiling at Multiplexion, Heidelberg, Germany), SW480 (ATCC CCL-228) colon carcinoma and A549 (ATCC CCL-185) non-small cell lung cancer cells (both kindly provided by the Institute of Cancer Research, Department of Medicine I, Medical University of Vienna, Austria) were harvested from adherent cultures in minimal essential medium (MEM; supplemented with 10% (*v*/*v*) heat-inactivated fetal calf serum (FCS from BioWest, Nuaillé, France), 4 mM l-glutamine, 1 mM sodium pyruvate and 1% (*v*/*v*) non-essential amino acid solution) by trypsinization and seeded in 100 µL aliquots in densities of 1 × 10^3^ (CH1/PA-1), 2 × 10^3^ (SW480) or 3 × 10^3^ (A549) cells/well into clear flat-bottom 96-well microculture plates. After 24 h incubation, 100 µL aliquots of test compound serially diluted in supplemented MEM were added in triplicates, with final DMSO content not exceeding 0.5% *v*/*v*. After incubation for 96 h, MEM was exchanged for 100 µL/well of a 1:7 *v*/*v* mixture of MTT dye (5 mg mL^−1^ 3-(4,5-dimethylthiazol-2-yl)-2,5-diphenyl-2H-tetrazolium bromide in phosphate-buffered saline) and RPMI 1640 medium (supplemented with 10% FCS and 4 mM l-glutamine). After incubation for another 4 h, mixtures were replaced with 150 µL DMSO per well and optical densities were measured with an ELx808 microplate reader (BioTek, Winooski, VT, USA) at 550 nm (and 690 nm as a reference). The 50% inhibitory concentrations (IC_50_) relative to untreated controls were interpolated from concentration–effect curves and averaged from three independent experiments.

### 3.6. DCFH-DA Assay

CH1/PA-1 and SW480 cells were harvested from adherent cultures as described in the Section 3.6 and seeded in 100 µL aliquots in densities of 2.5 × 10^4^ cells/well into clear flat-bottom 96-well microculture plates. After a 24 h incubation, cells were washed with 200 µL of Hanks′ balanced salt solution (HBSS; supplemented with 1% FCS), incubated for 45 min with 100 µL of 25 µM 2′,7′-dichlorodihydrofluorescein diacetate (DCFH-DA) in HBSS (with 1% FCS), and washed again with 200 µL of HBSS (with 1% FCS). Then, 200 µL aliquots of compound **1** or the positive control *tert*-butylhydroperoxide (TBHP), each of them diluted to 20 µM in phenol-red-free Opti-MEM (Gibco, Thermo Fisher Scientific, Waltham, MA, USA); supplemented with 1% FCS, were added in triplicates. In parallel, 100 µL aliquots of the two appropriately diluted compounds were added immediately after each other (and in reverse order) to further wells to yield triplicate combinations with final concentrations of 20 + 20 µM of the applied substances. Apart from these simultaneous combinations, consecutive combinations were tested in SW480 cells in additional plates by exchanging after 1 h the medium containing 20 µM of compound **1** for 200 µL aliquots of fresh medium containing 20 µM of TBHP (and in reverse order). Fluorescence (ex/em = 485/516 nm) was measured every 10 min over a total period of at least 2 h with a Synergy HT multimode microplate reader (BioTek, Winooski, VT, USA). Blank-corrected values relative to negative (untreated) controls were averaged from three independent experiments.

### 3.7. Isolated Compounds

#### 3.7.1. Artocarpinol A (**1**) 

Dark amorphous powder; yield 11.5 mg (=6.4 × 10^−4^% of the plant material); ${\left[\alpha \right]}_{D}^{20}$ = −310.00 (c 0.25 mg mL^−1^, MeOH); UV_(MeOH)_ *λ* nm (log ε): 204 (4.38), 224 (4.38), 260 (4.30), 284 (4.23), 318 (4.27), 336 (4.23), 352 (4.29); IR: 3364, 1616, 1521, 1466, 1454, 1287, 1155, 1065, 801 cm^−1^. The IR spectrum is depicted in Figure S47. HR-TOF-ESI-MS *m*/*z* 501.0856 [M − H]^−^ (calcd for C_27_H_17_O_10_ 501.0822); ^1^H NMR and ^13^C NMR data see Table 1. The 1D and 2D NMR spectra and the mass spectrum are shown in Figures S1–S8. 

#### 3.7.2. 3-*epi*-Artocarpinol A (**2**) 

Dark amorphous powder; yield 1.1 mg (=6.1 × 10^−5^%); ${\left[\alpha \right]}_{D}^{20}$ = −170.00 (c 0.25 mg mL^−1^, MeOH); UV_(MeOH)_ λ nm (log ε): 202 (4.33), 222 (4.32), 254 (4.39), 266 (4.24), 312 (4.19), 324 (4.21); IR: 3355, 2924, 2854, 1605, 1466, 1286, 1160, 1117, 1064 cm^−1^. The IR spectrum is depicted in Figure S49. HR-TOF-ESI-MS *m*/*z* 501.0835 [M − H]^−^ (calcd for C_27_H_17_O_10_ 501.0822); ^1^H NMR and ^13^C NMR data see Table 1. The 1D and 2D NMR spectra and the mass spectrum are shown in Figures S9–S16.

#### 3.7.3. Artocarpinol B (**3**) 

Dark amorphous powder; yield 1.5 mg (=8.3 × 10^−5^%); ${\left[\alpha \right]}_{D}^{20}$ = −222.00 (c 0.25 mg mL^−1^, MeOH); UV_(MeOH)_ *λ* nm (log ε): 204 (4.17), 224 (4.18), 260 (4.12), 284 (4.08), 318 (4.06), 336 (4.07), 352 (4.05); IR: 3364, 1615, 1461, 1313, 1165, 1120, 1048, 866, 784 cm^−1^. The IR spectrum is depicted in Figure S48. HR-TOF-ESI-MS *m*/*z* 501.0845 [M − H]^−^ (calcd for C_27_H_17_O_10_ 501.0822); ^1^H NMR and ^13^C NMR data see Table 1. The 1D and 2D NMR spectra and the mass spectrum are shown in Figures S17–S24. 

#### 3.7.4. Gambircatechol (**4**)

Dark amorphous powder; yield 2.0 mg (=1.1 × 10^−4^%); UV_(MeOH)_ λ nm (log ε): 204 (5.16), 224 (4.54), 264 (4.06), 296 (3.94), 322 (3.92); HR-TOF-ESI-MS *m*/*z* 395.0779 [M − H]^−^ (calcd for C_21_H_15_O_8_ 395.0767); ^1^H NMR and ^13^C NMR data in agreement with [17]. The ^1^H and ^13^C NMR spectra and the mass spectrum are shown in Figures S25–S32. NMR spectroscopic data are listed in Table S1. 

Oxidized form of gambircatechol (**4**): Dark amorphous powder; yield 1.3 mg (=7.2 × 10^−5^%); UV_(MeOH)_ λ nm (log ε): 220 (5.36), 254 (4.21), 308 (4.02), 459 (3.62), 692 (3.54); HR-TOF-ESI-MS *m*/*z* 393.0616 [M − H]^−^ (calcd for C_21_H_13_O_8_ 393.0610). The ^1^H and ^13^C NMR spectra mass spectrum is depicted in Figures S33–S35; comparison of UV spectra from gambircatechol (**4**) and oxidized form of gambircatechol are shown in Figure S45.

#### 3.7.5. (+)- Catechin (**5**)

White amorphous powder; yield 5.0 mg (=2.8 × 10^−4^%); HR-TOF-ESI-MS *m*/*z* 289.0720 [M − H]^−^ (calcd for C_15_H_13_O_6_ 289.0712); ^1^H NMR and ^13^C NMR data in agreement with [17]. The ^1^H and ^13^C NMR spectra are shown in Figures S36 and S37 and the mass spectrum is depicted in Figure S38. NMR spectroscopic data are listed in Table S1.

#### 3.7.6. (+)- Afzelechin (**6**)

White amorphous powder; yield 3.1 mg (=1.7 × 10^−4^%); HR-TOF-ESI-MS *m*/*z* 273.0773 [M − H]^−^ (calcd for C_15_H_13_O_5_ 273.0763); ^1^H NMR and ^13^C NMR data in agreement with [20]. The ^1^H and ^13^C NMR spectra are shown in Figures S39 and S40 and the mass spectrum is depicted in Figure S41. NMR spectroscopic data are listed in Table S1.

#### 3.7.7. Oxyresveratrol (**7**)

White amorphous powder; yield: 67.0 mg (1.7 × 10^−3^%); HR-TOF-ESI-MS *m*/*z* 267.0621 [M + Na]^+^ (calcd for C_14_H_12_O_4_Na 267.0633); ^1^H NMR and ^13^C NMR data in agreement with [13]. The ^1^H and ^13^C NMR spectra are shown in Figures S42 and S43 and the mass spectrum is depicted in Figure S44.

## 4. Conclusions

Chromatographic separation of the methanolic leaf extract of *Artocarpus lacucha* yielded in sum seven flavan-3-ol derivatives, whereas three of them (**1**, **3** and **4**) were very likely derived from (+)-catechin (**5**) and one (**2**) might be derived from 3-*epi*-catechin. Compounds **1**–**3** are described for the first time. From the stem bark extract of this species, the stilbenoid oxyresveratrol (**7**) could be isolated in higher quantities, which may contract its general ascribed role as phytoalexin in planta. Compounds **1**–**5** possess hydroquinone type structural moieties, which are easily oxidizable to *ortho*-quinone moieties, which, however, have only been detected by HR-ESI-MS. All of them show a highly aromatic core structure with either one benzofuran moiety (in **4**) or with two benzofuran moieties in compounds **1**–**3**, attached to the flavan-3-ol core structure. Compounds **1**–**4** exhibited strong radical scavenging activities, which were assessed by employing the DPPH assay. Furthermore, **1** also showed antiproliferative and antioxidative properties in the two cancer cell lines CH1/PA-1 and SW480. These radical scavenger activities may also contribute to the defense mechanism of plant species against the formation of reactive oxygen species. Overall, these results contribute to the phytochemical knowledge of this plant species and showed the bandwidth of possible biosynthetic modifications of flavan-3-ol derivatives.

## Figures and Tables

**Table 1:** NMR spectroscopic data [ppm] of artocarpinol A (**1**) 3-*epi*-artocarpinol A (**2**) and artocarpinol B (**3**) recorded in CD_3_OD. Positions of the carbon atoms are demonstrated in Figure 1. The relative integral, the multiplicity and the coupling constants (Hz) are provided. In addition, the ^13^C NMR chemical shifts and multiplicities are given.

Position	Artocarpinol A (1)		3-*epi*-Artocarpinol A (2)		Artocarpinol B (3)	
	δ_H_ (*J* in Hz)	δ_C_	δ_H_ (*J* in Hz)	δ_C_	δ_H_ (*J* in Hz)	δ_C_
Catechin moiety						
2	5.70 (1H, d, 3.1)	80.4, d	5.65 (1H, d, 3.3)	79.7, d	4.96 (1H, d, 7.6)	83.7, d
3	4.51 (1H, q, 3.9)	67.4, d	4.47 (1H, ddd, 8.1, 5.0, 3.3)	67.3, d	4.28 (1H, td, 8.0, 5.3)	68.1, d
4	2.89 (1H, ddd, 16.8, 3.7, 1.7) 2.68 (1H, dd, 16.7, 4.3)	25.5, t	3.11 (1H, ddd, 15.9, 5.0, 1.2) 2.72 (1H, dd, 15.9, 7.7)	27.9, t	3.34 (1H, dd, 15.8, 5.3) 3.05 (1H, dd, 15.8, 8.2)	28.6, t
5	-	156.0, s	-	155.8, s	-	155.3, s
6	6.63 (1H, s)	91.2, d	6.65 (1H, s)	91.3, d	-	109.8, s
7	-	158.0, s	-	158.2, s	-	149.4, s
8	-	107.4, s	-	107.4, s	-	104.6, s
9	-	148.7, s	-	149.4, s	-	148.4, s
10	-	102.9, s	-	103.6, s	-	100.5, s
1′	-	132.1, s	-	130.7, s	-	131.9, s
2′	6.82 (1H, d, 2.0)	113.9, d	7.01 (1H, d, 2.1)	116.0, d	7.01 (1H, d, 2.1)	115.2, d
3′	-	145.9, s	-	145.8, s	-	146.5, s
4′	-	146.3, s	-	146.1, s	-	146.4, s
5′	6.68 (1H, d, 8.3)	116.4, d	6.68 (1H, d, 8.2)	115.8, d	6.82 (1H, d, 8.1)	116.2, d
6′	6.73 (1H, dd, 8.3, 2.1)	120.3, d	6.87 (1H, dd, 8.5, 2.0)	120.4, d	6.89 (1H, dd, 8.2, 2.1)	120.2, d
D-ring						
1″	-	108.7, s	-	108.6, s	-	115.1, s
2″		119.7, s	-	119.7, s	7.47 (1H, s)	107.2, d
3″	-	141.7, s	-	141.9, s	-	143.5, s
4″	-	143.6, s	-	143.6, s	-	146.0, s
5″	7.00 (1H, s)	98.2, d	6.98 (1H, s)	98.1, d	7.05 (1H, s)	99.1, d
6″	-	153.8, s		153.8, s	-	151.7, s
E-ring						
1‴	-	117.7, s	-	117.5, s	-	116.3, s
2‴	8.45 (1H, s)	111.7, d	8.34 (1H, s)	111.9, d	7.38 (1H, s)	108.1, d
3‴	-	142.5, s	-	142.4, s	-	143.2, s
4‴	-	147.1, s	-	147.1, s	-	145.7, s
5‴	7.00 (1H, s)	96.3, d	6.97 (1H, s)	96.3, d	7.09 (1H, s)	99.3, d
6‴	-	152.8, s	-	152.5, s	-	151.5, s

## Data Availability

The data, except the data from the bioassays, are available in this article.

## Supplementary Materials

The following are available online at. 1D and 2D-NMR and mass spectra of the isolated compounds as well as IR spectra of compounds **1**–**3**.

## Sample Availability

Samples of the compounds are not available from the authors.
