# Supplementary material for: Highly Aromatic Flavan-3-ol Derivatives from Palaeotropical Artocarpus lacucha Buch.-Ham Possess Radical Scavenging and Antiproliferative Properties
Source: Molecules. 2021 Feb 18;26(4):1078. doi: 10.3390/molecules26041078 (PMC7922997; doi:10.3390/molecules26041078)
Supplement: Supplementary file 1 [file molecules-26-01078-s001.pdf]

SUPPLEMENTARY MATERIAL

Highly Aromatic Flavan-3-ol Derivatives from Palaeotropical *Artocarpus lacucha* Buch.-Ham Possess Radical Scavenging and Antiproliferative Properties

Weerasak Songoen [a, c], Witthawat Phanchai [b], Lothar Brecker [c], Dominik Wenisch [d], Michael A. Jakupec [d], Wanchai Pluempanupat [a,*], Johann Schinnerl [e,*]

[a] *Department of Chemistry and Center of Excellence for Innovation in Chemistry, Special Research Unit for Advanced Magnetic Resonance, Faculty of Science, Kasetsart University, Bangkok 10900, Thailand.*

[b] *Department of Physics, Faculty of Science, Khon Kaen University, Khon Kaen 40002, Thailand.*

[c] *Department of Organic Chemistry, Faculty of Chemistry, University of Vienna, Währinger Strasse 38, A-1090 Vienna, Austria.*

[d] *Institute of Inorganic Chemistry, Faculty of Chemistry, University of Vienna, Währinger Strasse 42, A-1090 Vienna, Austria.*

[e] *Department of Botany and Biodiversity Research, Faculty of Life Science, University of Vienna, Rennweg 14, A-1030 Vienna, Austria.*

* Corresponding authors

Wanchai Pluempanupat (fsciwcp@ku.ac.th)

Johann Schinnerl (johann.schinnerl@univie.ac.at)

Figure S1. ^{1}H NMR of artocarpinol A (**1**) in CD_3OD. 4
Figure S2. ^{13}C NMR of artocarpinol A (**1**) in CD_3OD. 5
Figure S3. COSY of artocarpinol A (**1**) in CD_3OD. 6
Figure S4. TOCSY artocarpinol A (**1**) in CD_3OD. 6
Figure S5. HSQC of artocarpinol A (**1**) in CD_3OD. 7
Figure S6. HMBC of artocarpinol A (**1**) in CD_3OD. 7
Figure S7. NOESY of artocarpinol A (**1**) in CD_3OD. 8
Figure S8. Mass spectrum of artocarpinol A (**1**) 9
Figure S9. ^{1}H NMR of 3-epi-artocarpinol A (**2**) in CD_3OD. 10
Figure S10. ^{13}C NMR of 3-epi-artocarpinol A (**2**) in CD_3OD**.** 11
Figure S11. COSY of 3-epi-artocarpinol A (**2**) in CD_3OD. 12
Figure S12. TOCSY 3-epi-artocarpinol A (**2**) in CD_3OD. 12
Figure S13. HSQC of 3-epi-artocarpinol A (**2**) in CD_3OD. 13
Figure S14. HMBC of 3-epi-artocarpinol A (**2**) in CD_3OD. 13
Figure S15. NOESY of 3-epi-artocarpinol A (**2**) in CD_3OD. 14
Figure S16. Mass spectrum of 3-epi-artocarpinol A (**2**). 15
Figure S17. ^{1}H NMR of artocarpinol B (**3**) in CD_3OD. 16
Figure S18. ^{13}C NMR of artocarpinol B (**3**) in CD_3OD. 17
Figure S19. COSY of artocarpinol B (**3**) in CD_3OD. 18
Figure S20. TOCSY of artocarpinol B (**3**) in CD_3OD. 18
Figure S21. HSQC of artocarpinol B (**3**) in CD_3OD. 19
Figure S22. HMBC of artocarpinol B (**3**) in CD_3OD. 19
Figure S23. NOESY of artocarpinol B (**3**) in CD_3OD. 20
Figure S24. Mass spectrum of artocarpinol B (**3**). 21
Figure S25. ^{1}H NMR of gambircatechol (**4**) in CD_3OD. 22
Figure S26. ^{13}C NMR of gambircatechol (**4**) in CD_3OD. 23
Figure S27. COSY of gambircatechol (**4**) in CD_3OD. 24
Figure S28. TOCSY of gambircatechol (**4**) in CD_3OD. 24
Figure S29. HSQC of gambircatechol (**4**) in CD_3OD. 25
Figure S30. HMBC of gambircatechol (**4**) in CD_3OD. 25
Figure S31. NOESY of gambircatechol (**4**) in CD_3OD. 26
Figure S32. Mass spectrum of gambircatechol (**4**). 27
Figure S33. ^{1}H NMR of ortho-quinone of **4** in DMSO-d_6. 28
Figure S34. ^{13}C NMR of ortho-quinone of **4** in DMSO-d_6. 29

Figure S35. Mass spectrum of ortho-quinone of **4**. 30
Figure S36. ^{1}H NMR of (+)-catechin (**5**) in CD_3OD. 31
Figure S37. ^{13}C NMR of (+)-catechin (**5**) in CD_3OD. 32
Figure S38. Mass spectrum of (+)-catechin (**5**). 33
Figure S39. ^{1}H NMR of (+)-afzelechin (**6**) in CD_3OD. 34
Figure S40. ^{13}C NMR of (+)-afzelechin (**6**) in CD_3OD. 35
Figure S41. Mass spectrum of (+)-afzelechin (**6**). 36
Figure S42. ^{1}H NMR of oxyresveratrol (**7**) in CD_3OD. 37
Figure S43. ^{13}C NMR of oxyresveratrol (**7**) in CD_3OD. 38
Figure S44. Mass spectrum of oxyresveratrol (**7**). 39
Figure S45. UV spectra of gambircatechol (**4**; blue) and the ortho-quinone (red). 40
Figure S46. HPLC profile of the stem extract at 230 nm. **7** = oxyresveratrol. 40
Figure S47. IR spectrum of artocarpinol A (**1**). 41
Figure S48. IR spectrum of 3-epi-artocarpinol A (**2**). 41
Figure S49. IR spectrum of artocarpinol B (**3**). 42
Table S1. 43

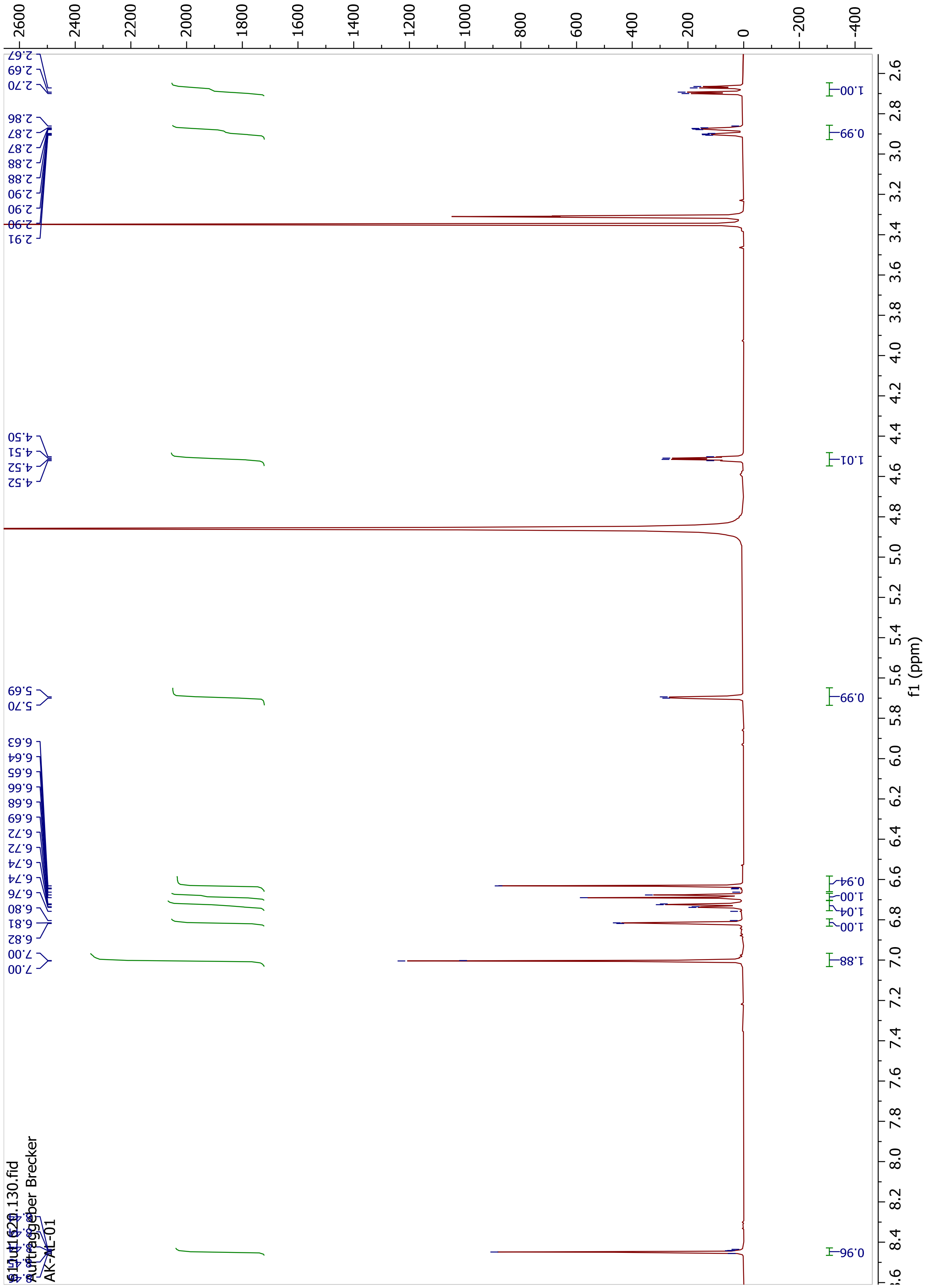

Figure S1. ^{1}H NMR of artocarpinol A (**1**) in CD_3OD.

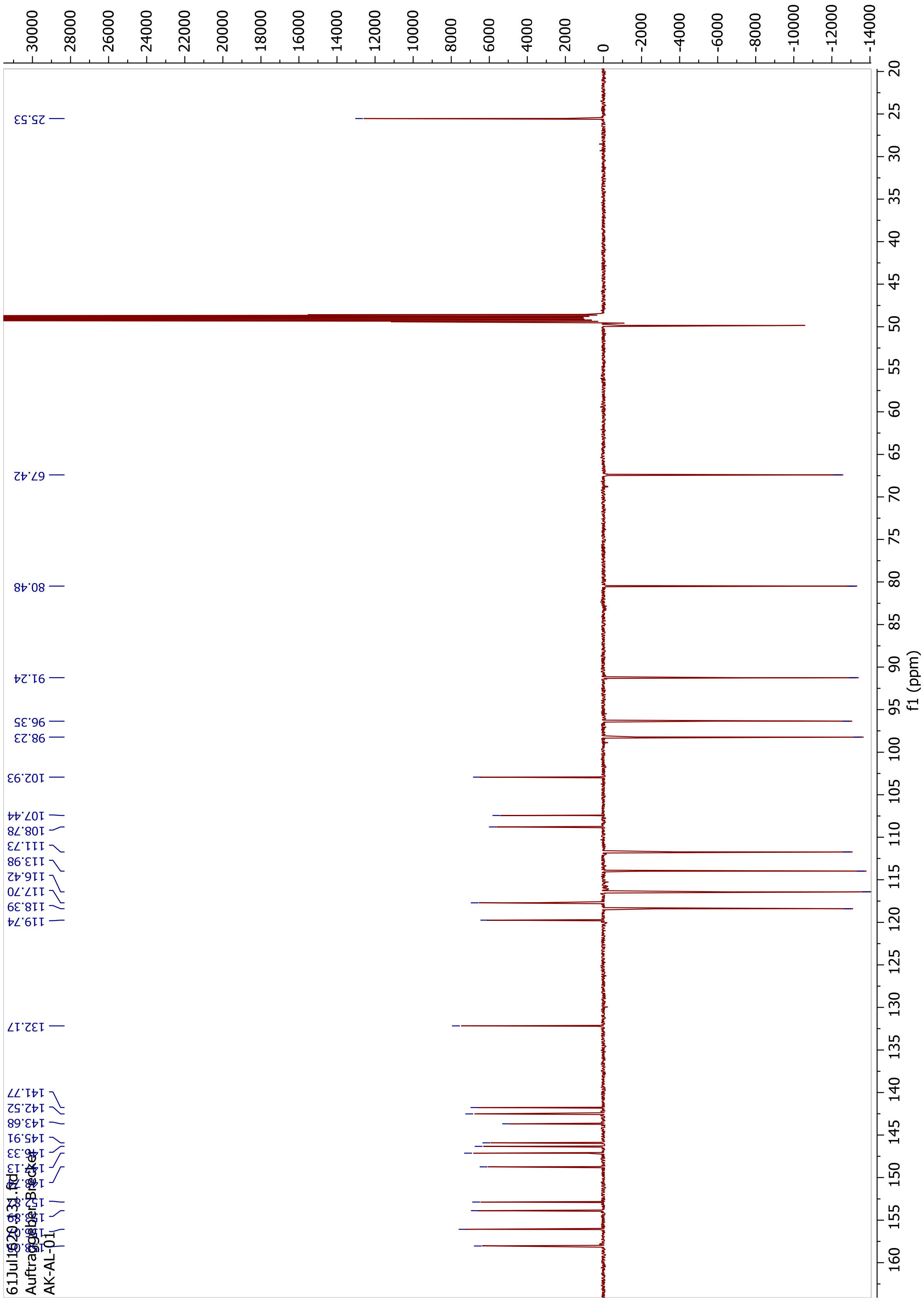

Figure S2. ^{13}C NMR of artocarpinol A (**1**) in CD_3OD.

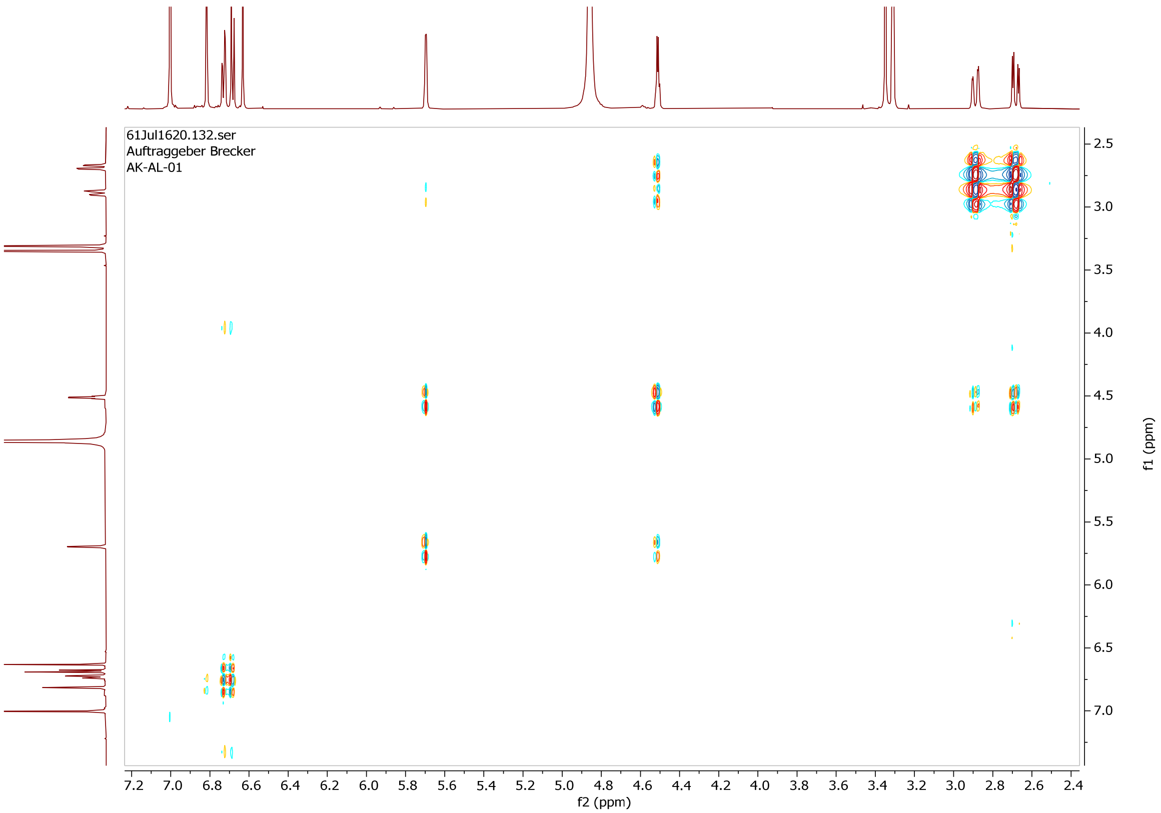

Figure S3. COSY of artocarpinol A (**1**) in CD_3OD.

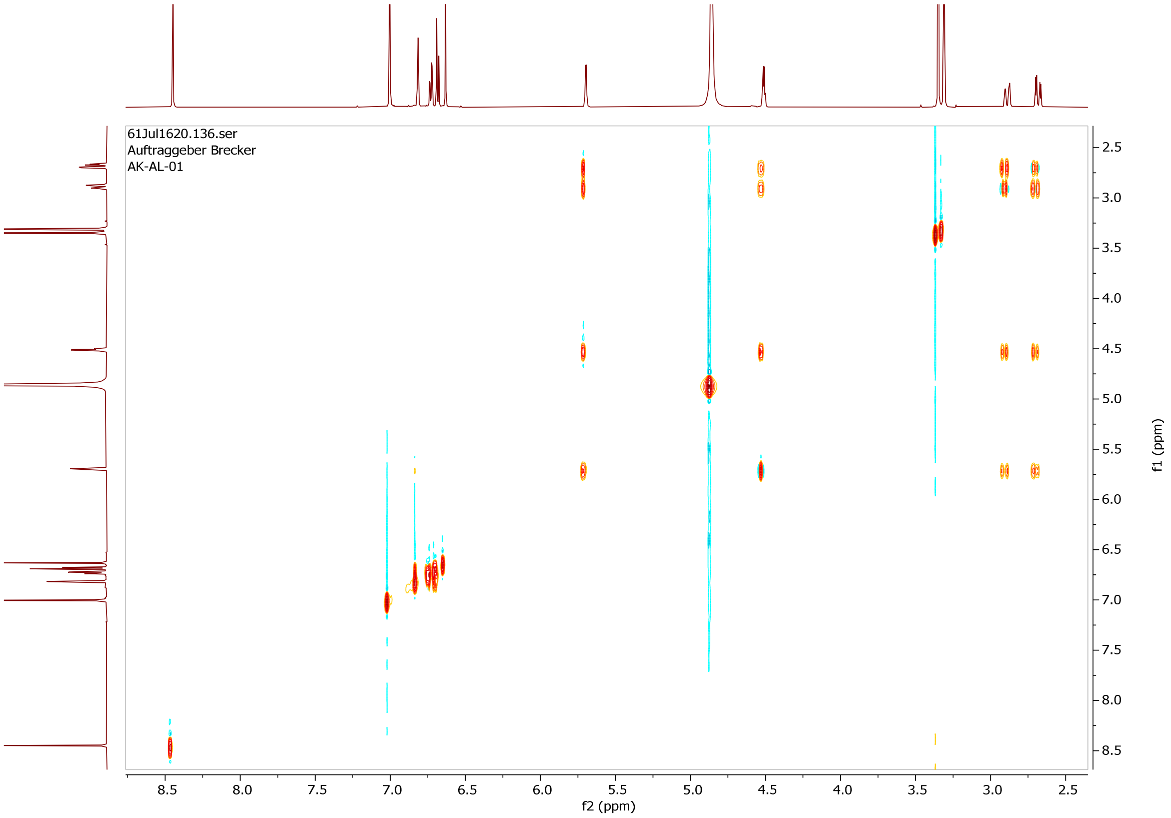

Figure S4. TOCSY artocarpinol A (**1**) in CD_3OD.

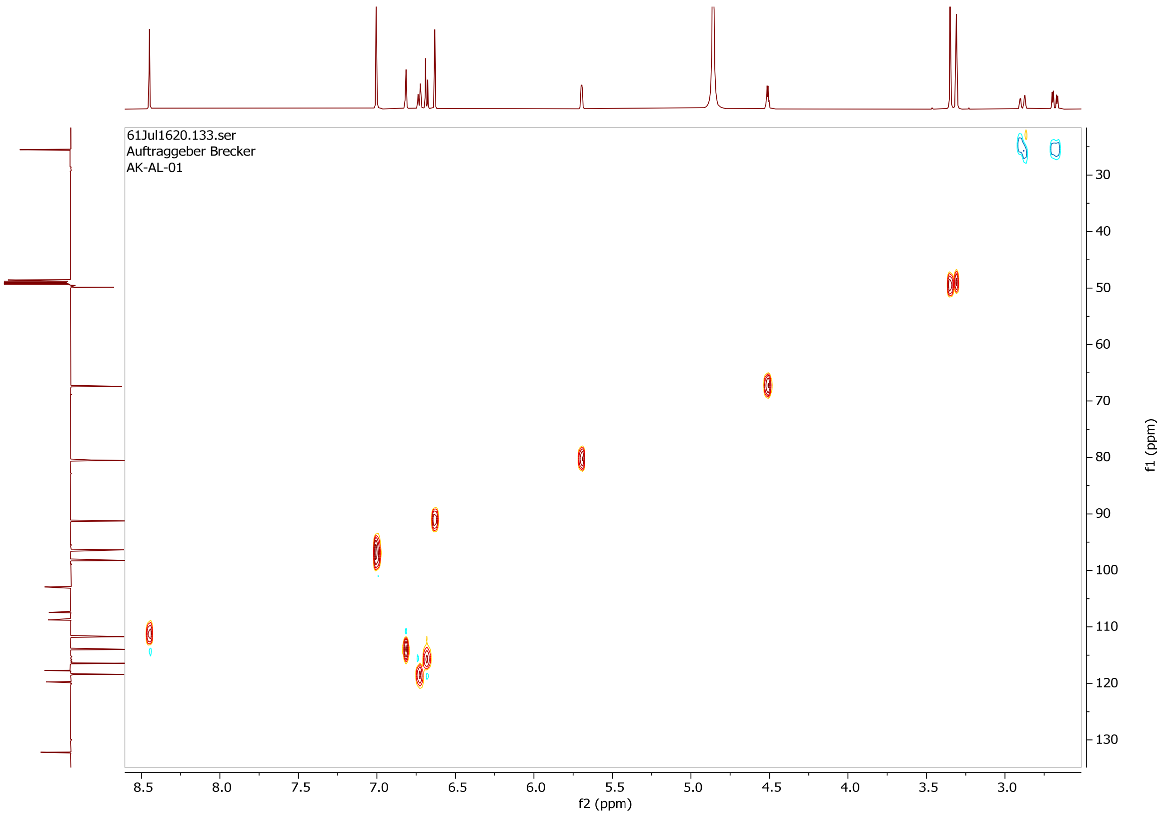

Figure S5. HSQC of artocarpinol A (**1**) in CD_3OD.

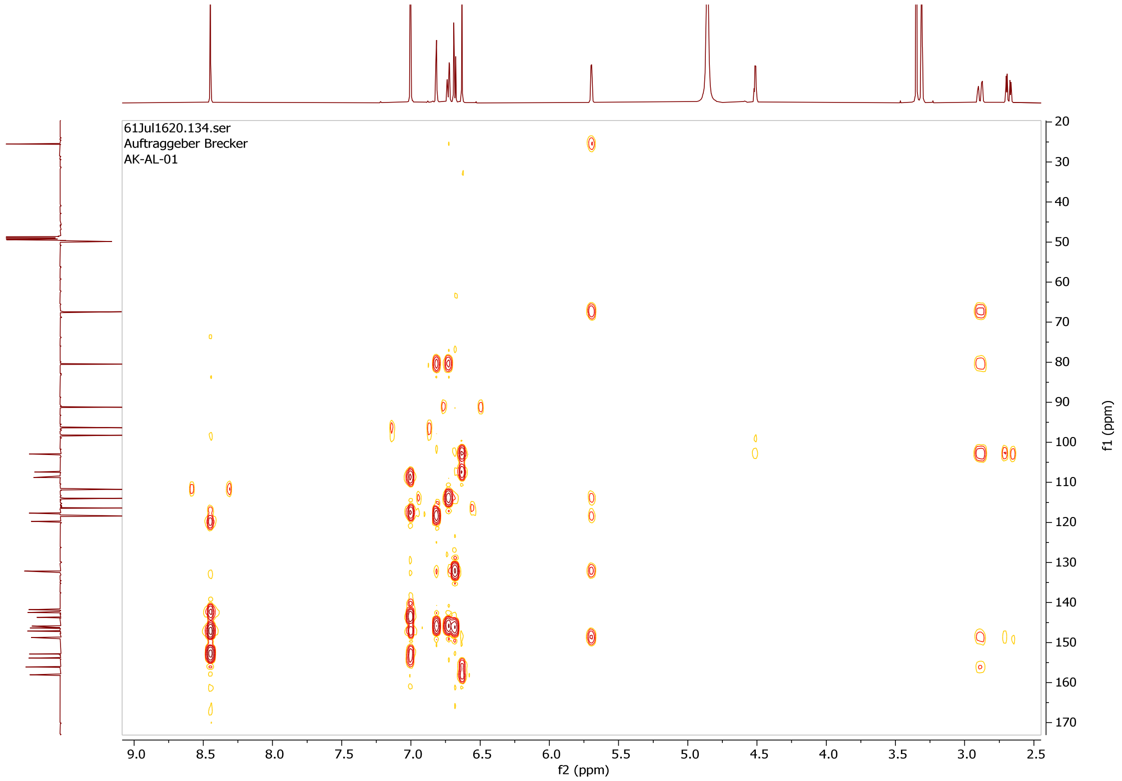

Figure S6. HMBC of artocarpinol A (**1**) in CD_3OD.

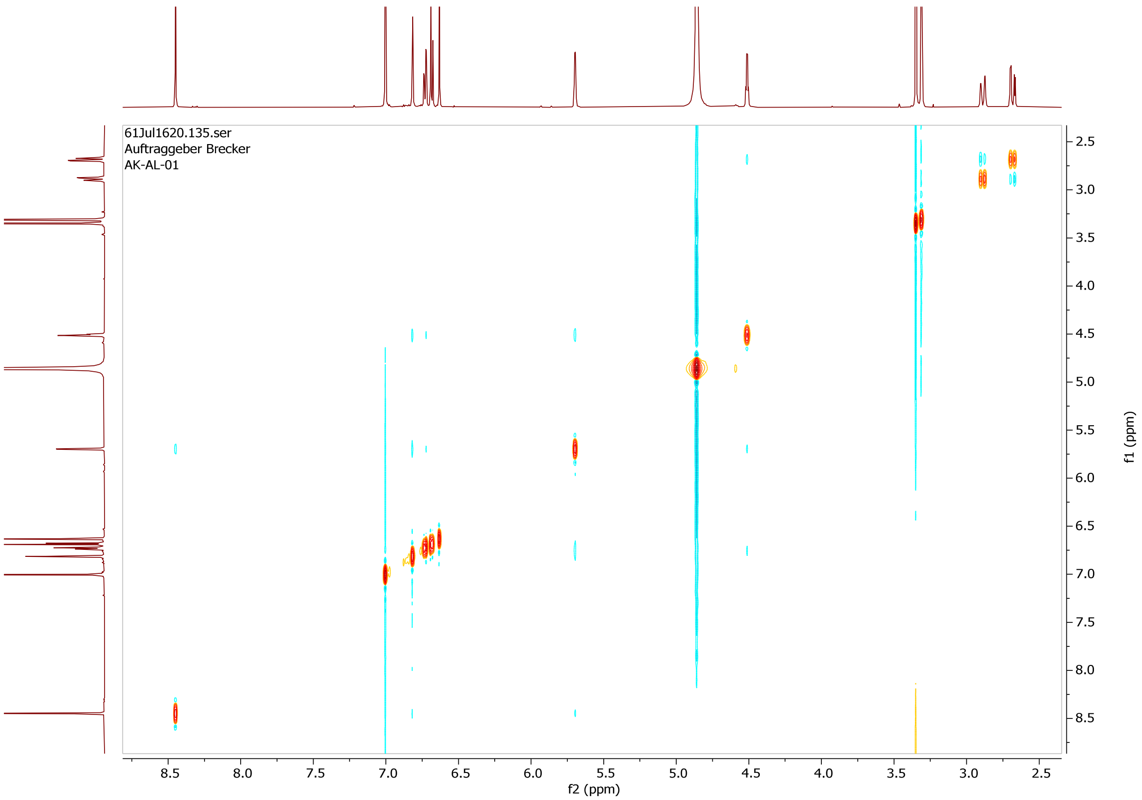

Figure S7. NOESY of artocarpinol A (**1**) in CD_3OD.

Generic Display Report

Analysis Info

Analysis Name	E:\Data\MS_MessService\71710000004.d	Acquisition Date	7/17/2020 12:05:26 PM
Method	tune_low_MS_Service_07_20.m	Operator	msc
Sample Name	AK-AL-01	Instrument	maXis
Comment	Schedl / Brecker / Botanik / Org.Chem.<br>Ergebnis +/- 5ppm<br>ACN / MeOH + 1 % H2O		

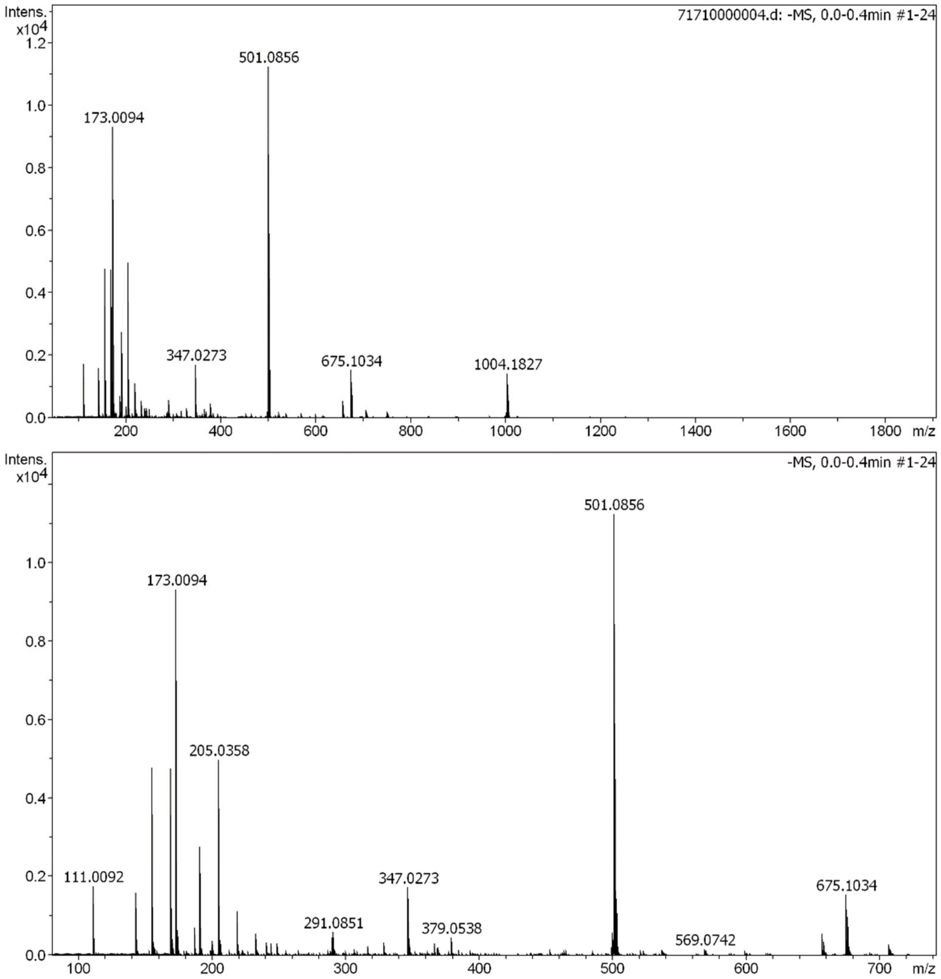

Bruker Compass DataAnalysis 4.1 printed: 7/17/2020 12:10:28 PM by: msc Page 1 of 1

Figure S8. Mass spectrum of artocarpinol A (**1**)

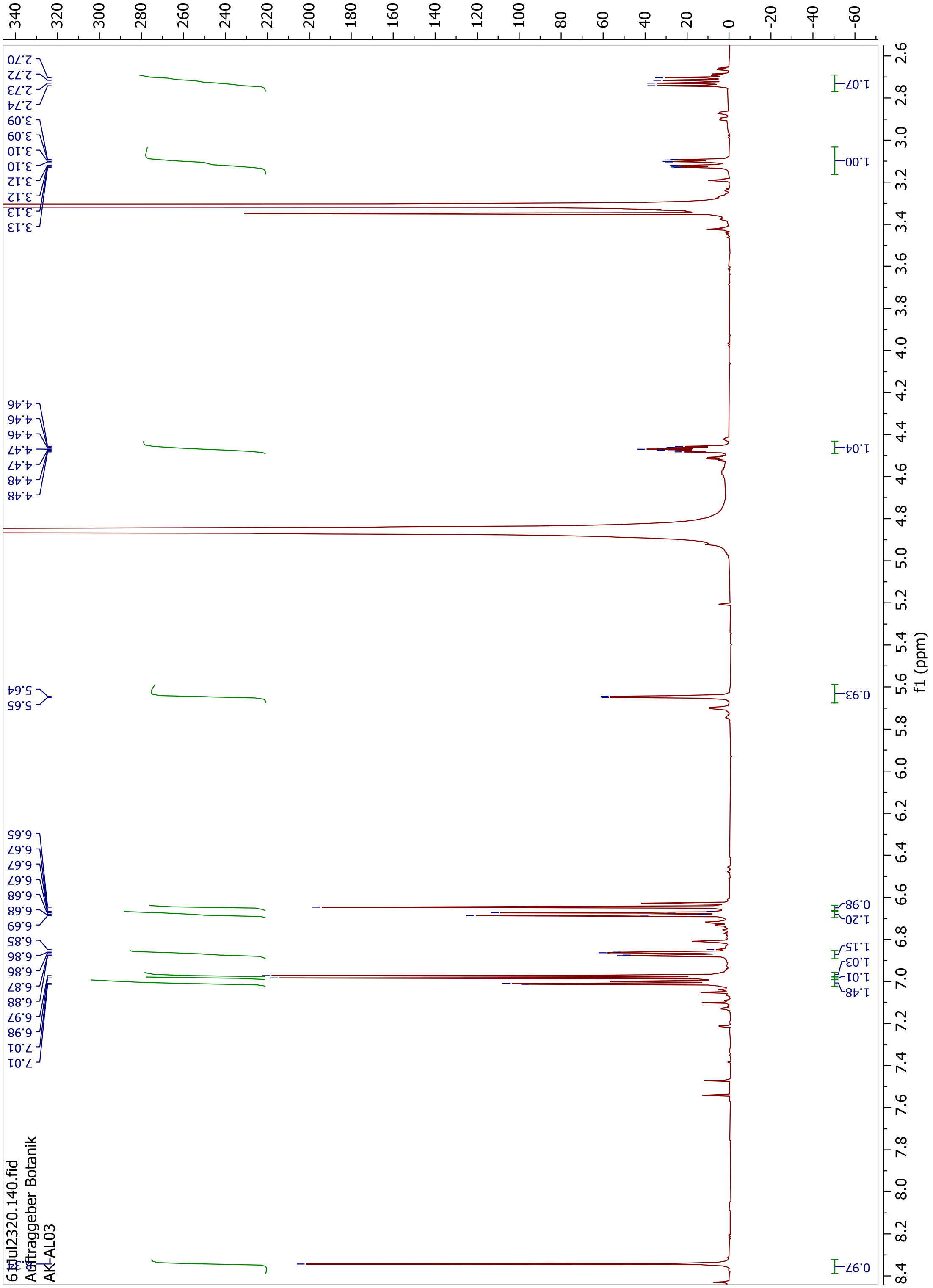

Figure S9. ^{1}H NMR of 3-*epi*-artocarpinol A (**2**) in CD_3OD.

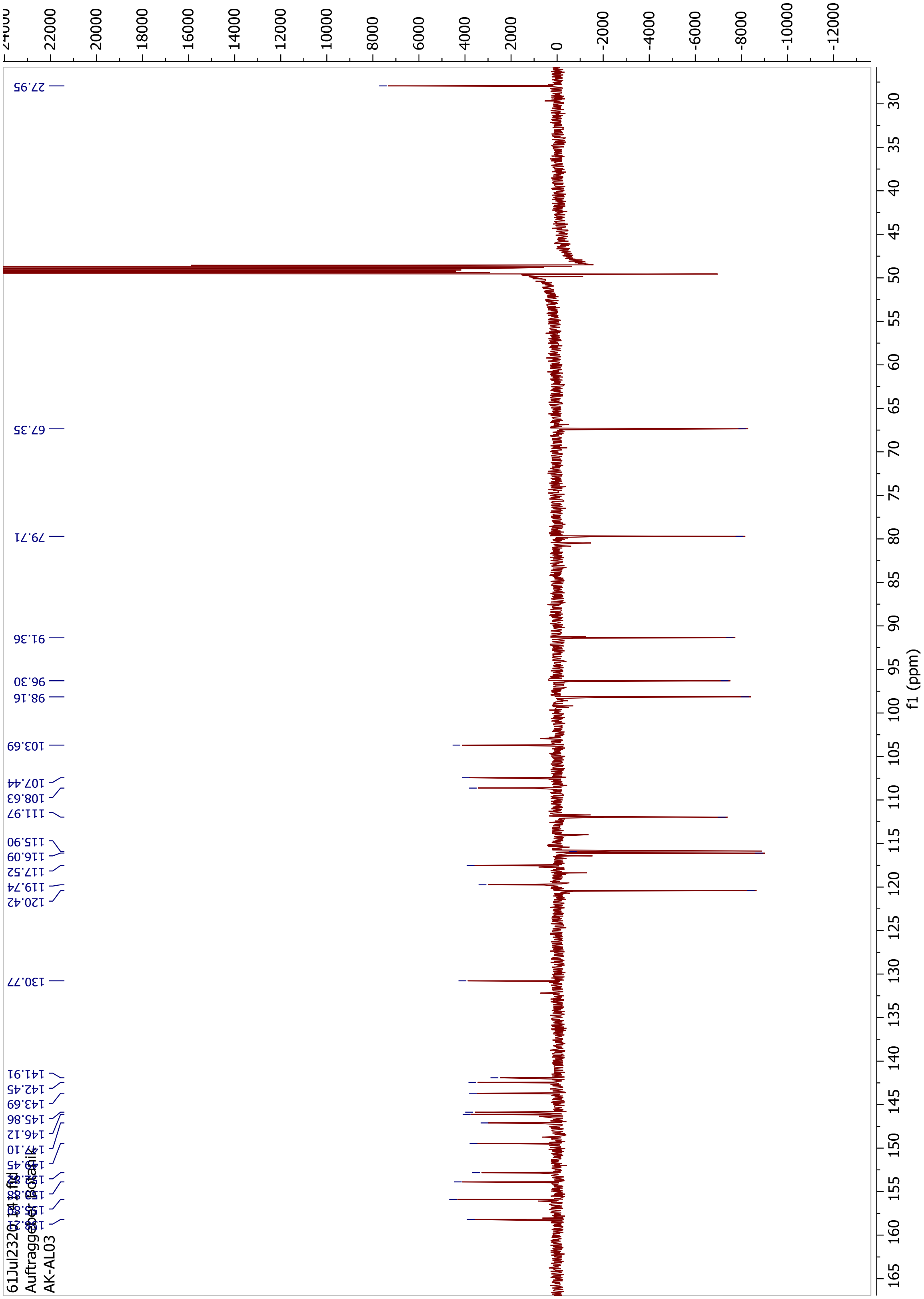

Figure S10. ^{13}C NMR of 3-*epi*-artocarpinol A (**2**) in CD_3OD**.**

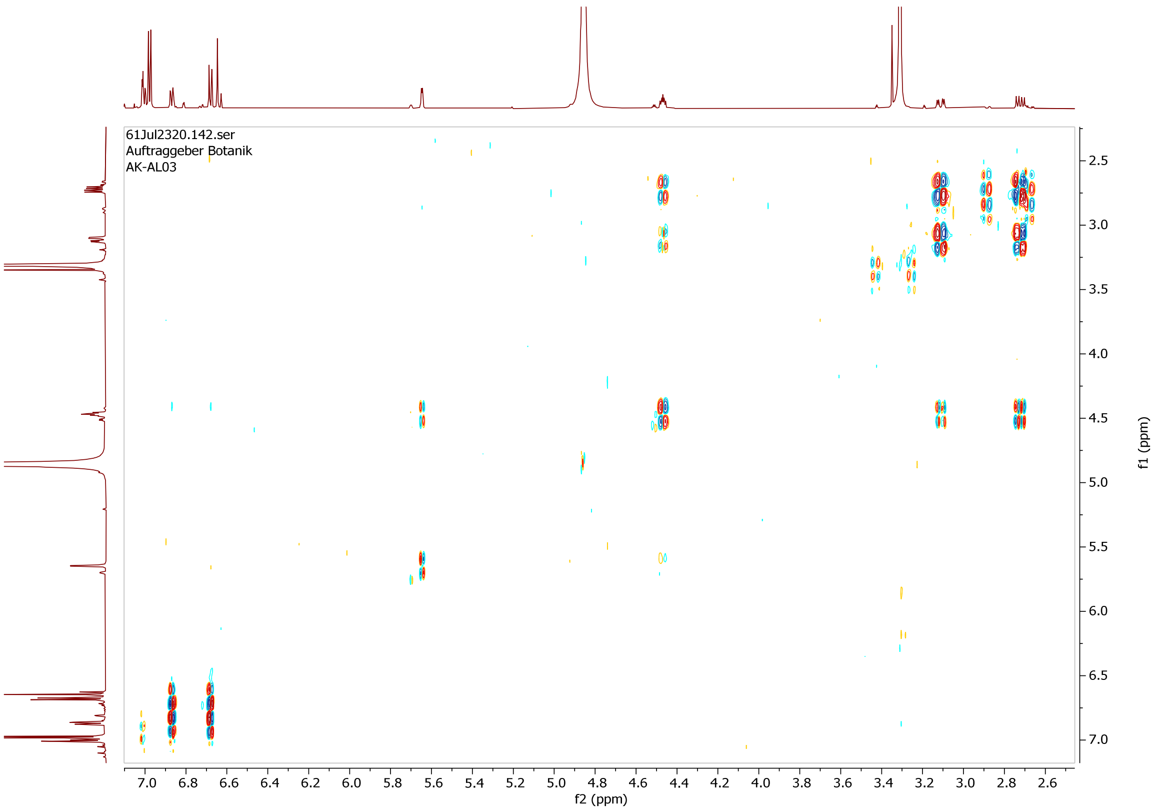

Figure S11. COSY of 3-*epi*-artocarpinol A (**2**) in CD_3OD.

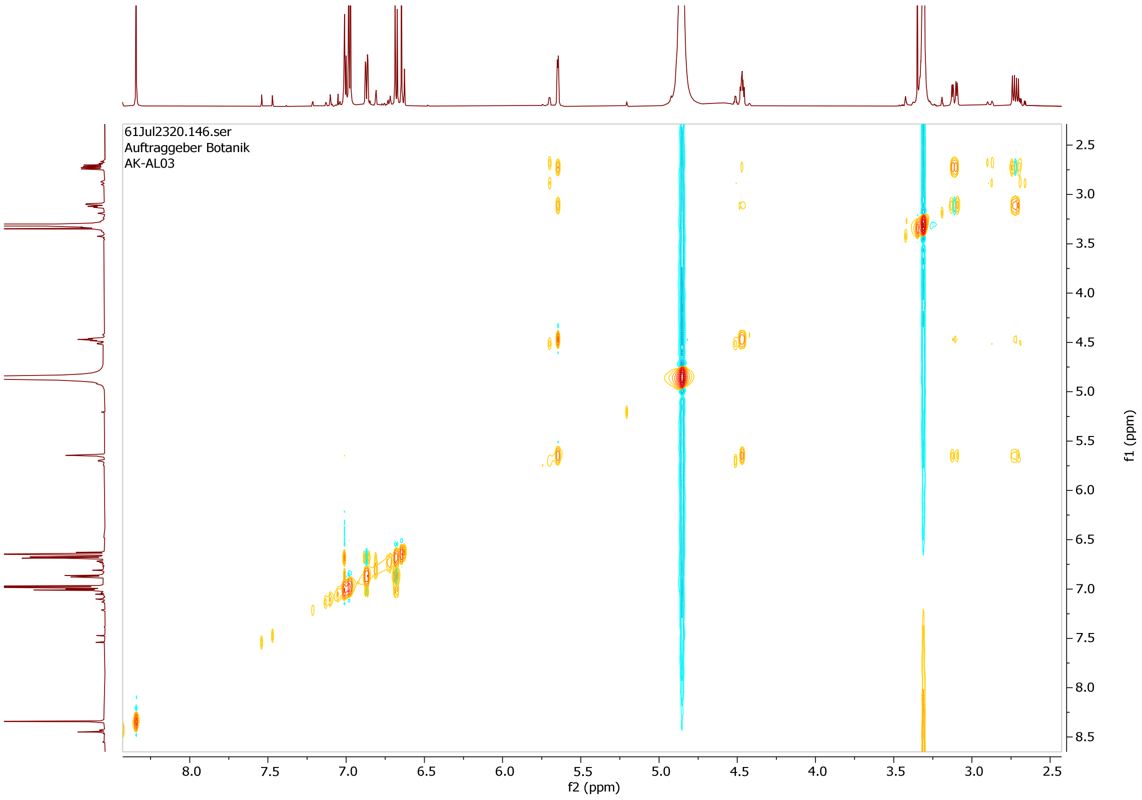

Figure S12. TOCSY 3-*epi*-artocarpinol A (**2**) in CD_3OD.

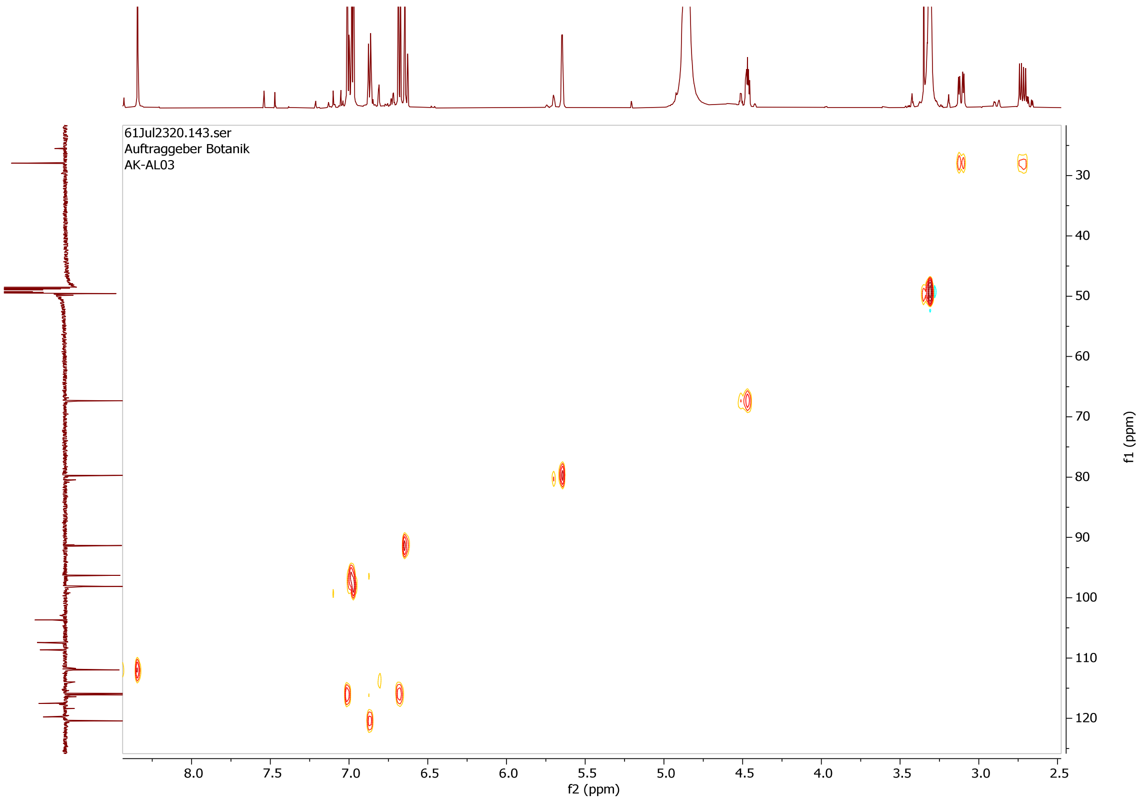

Figure S13. HSQC of 3-*epi*-artocarpinol A (**2**) in CD_3OD.

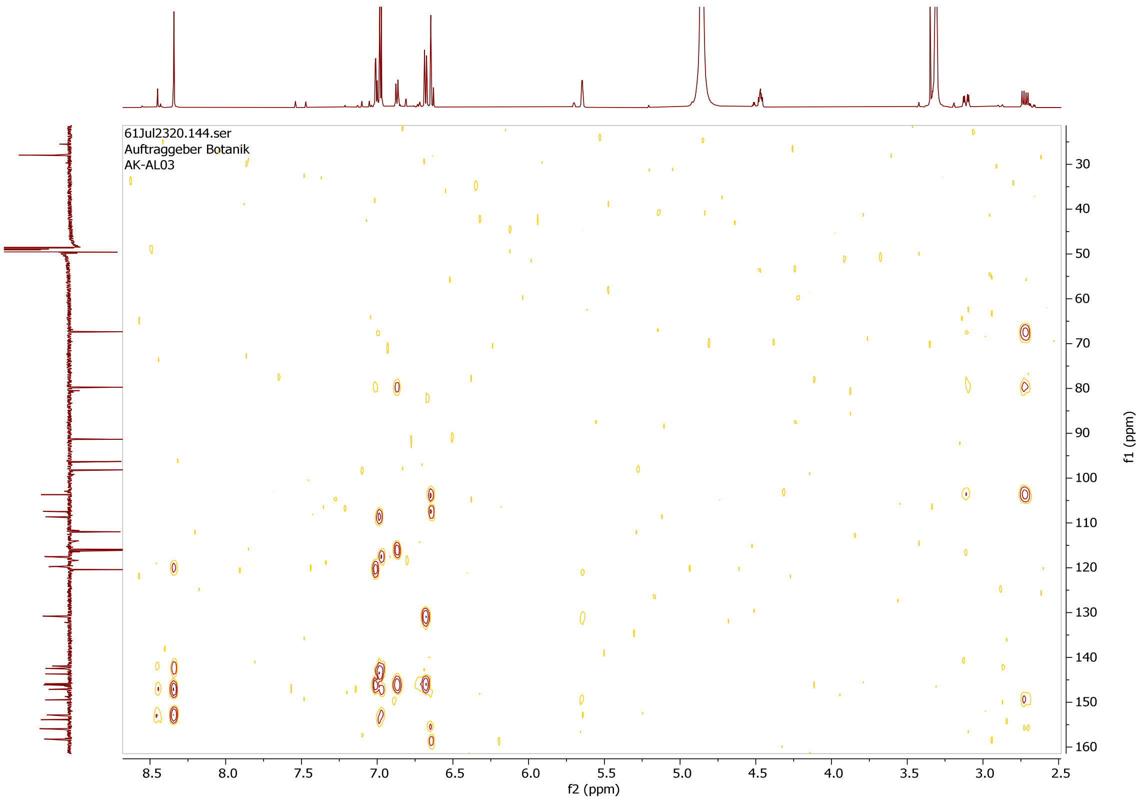

Figure S14. HMBC of 3-*epi*-artocarpinol A (**2**) in CD_3OD.

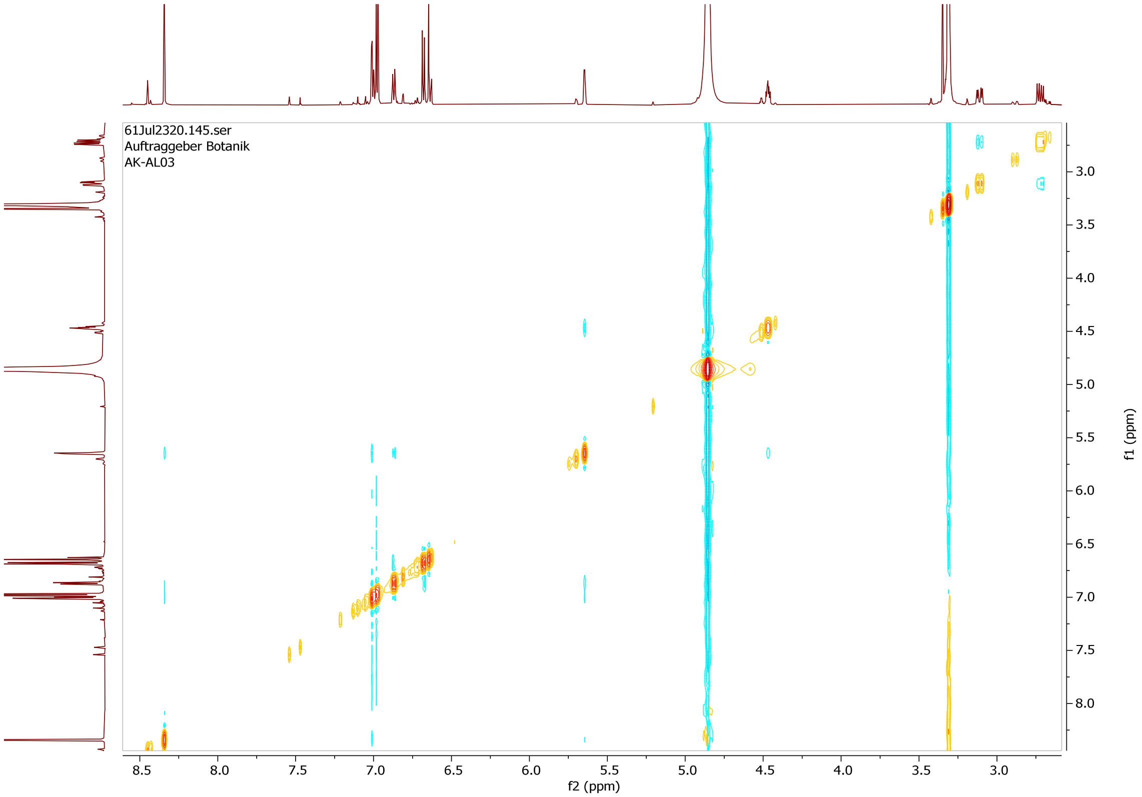

Figure S15. NOESY of 3-*epi*-artocarpinol A (**2**) in CD_3OD.

Generic Display Report

**Analysis Info**		Acquisition Date	7/24/2020 2:59:18 PM
Analysis Name	E:\Data\MS_MessService\71873000002.d		
Method	tune_low_MS_Service_07_20.m	Operator	msc
Sample Name	AK-AL-03	Instrument	maXis
Comment	Schedl / Brecker / Org.Chem.<br>ACN / MeOH + 1 % H2O<br>Ergebnis +/- 5 ppm		

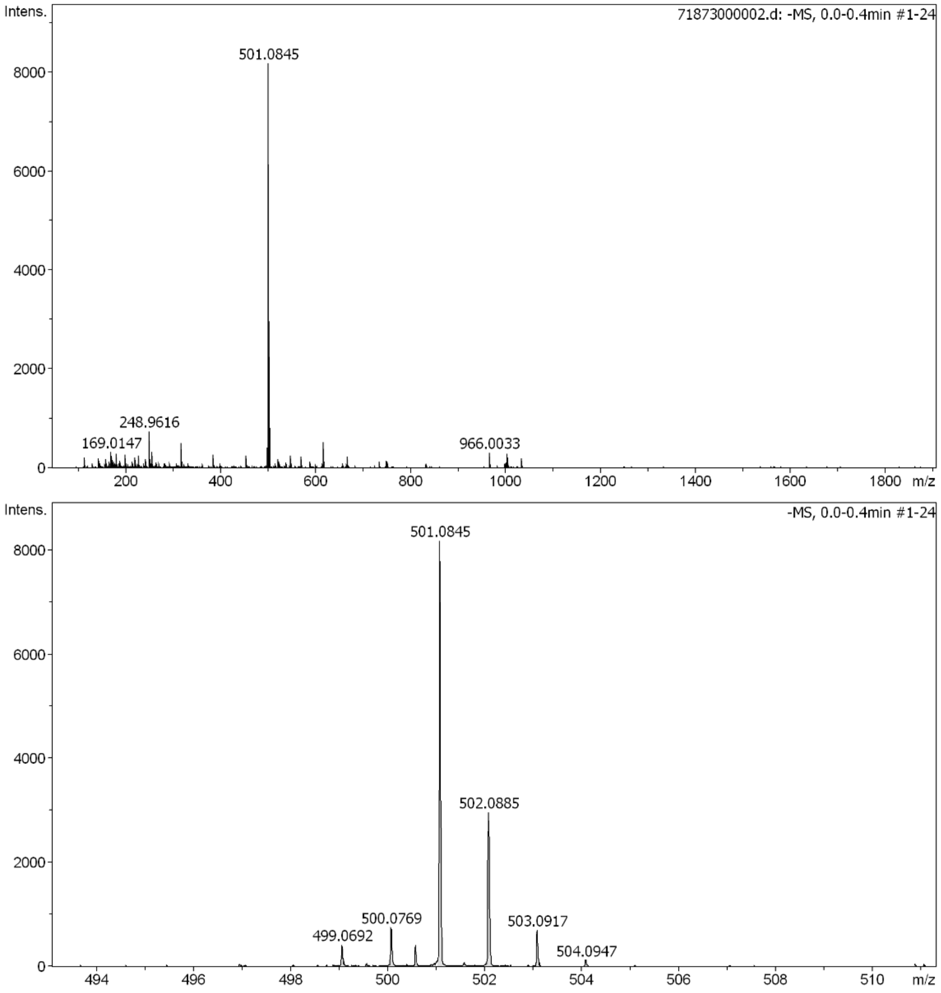

Bruker Compass DataAnalysis 4.1 printed: 7/24/2020 3:03:16 PM by: msc Page 1 of 1

Figure S16. Mass spectrum of 3-*epi*-artocarpinol A (**2**).

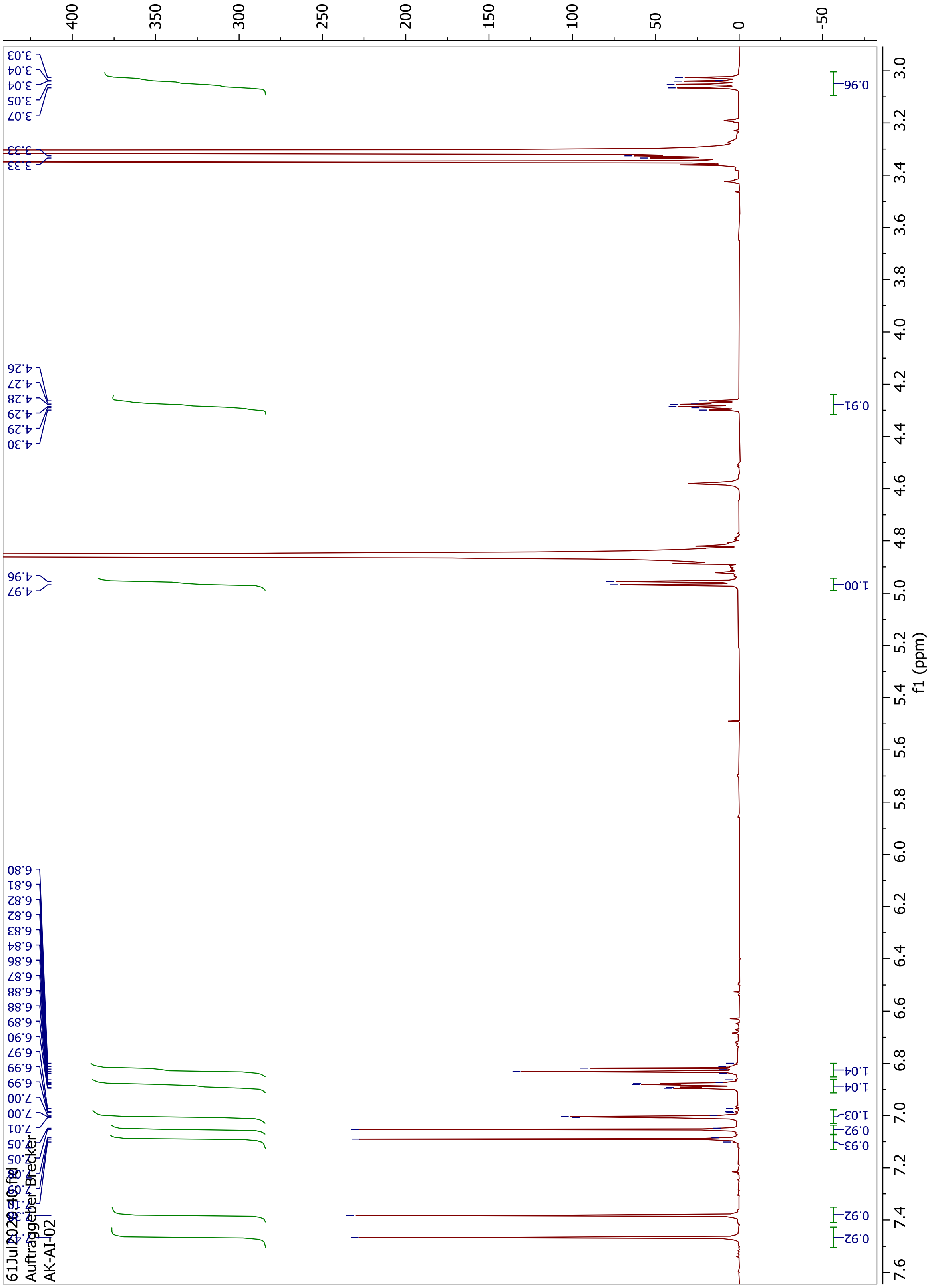

Figure S17. ^{1}H NMR of artocarpinol B (**3**) in CD_3OD.

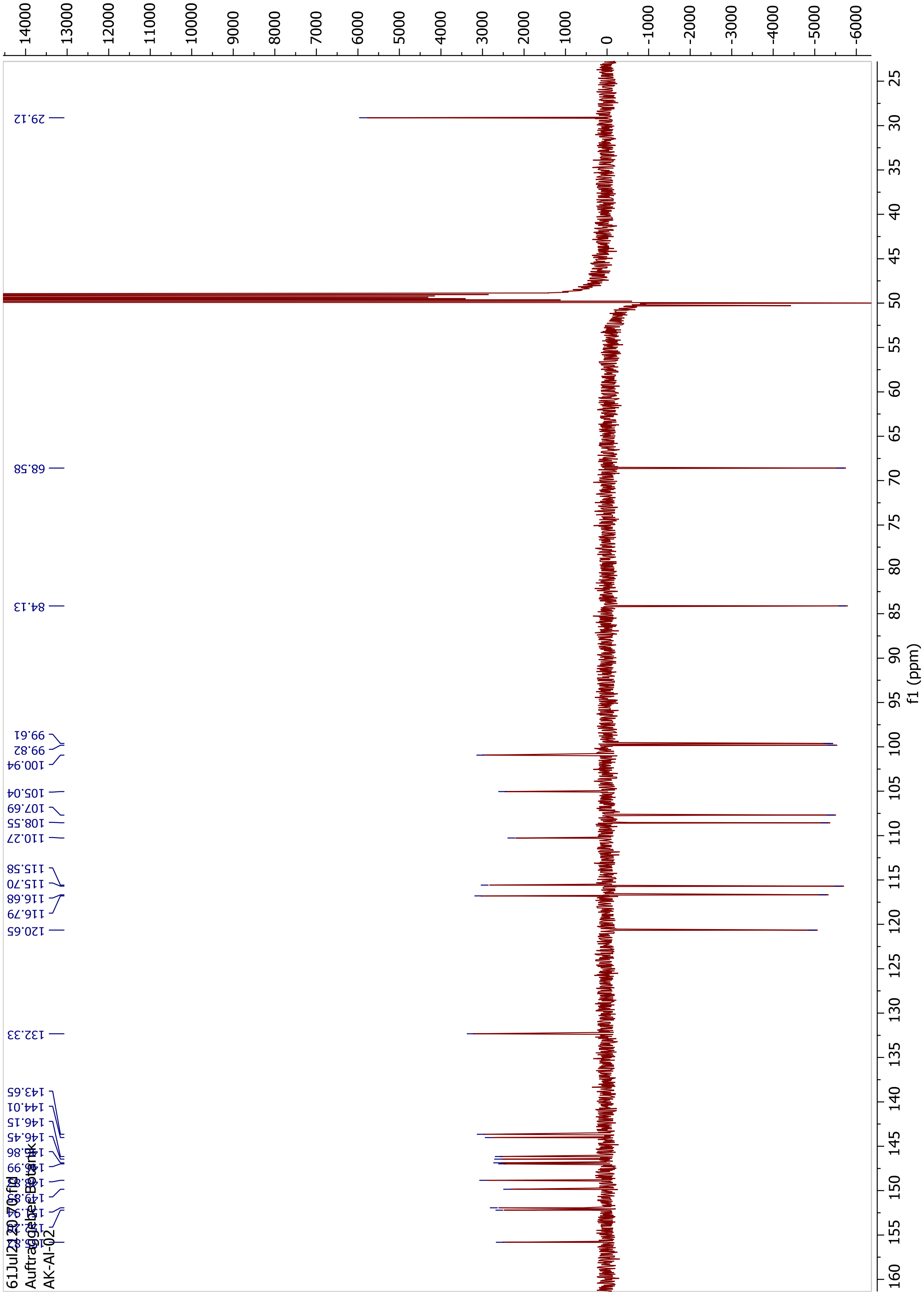

Figure S18. ^{13}C NMR of artocarpinol B (**3**) in CD_3OD.

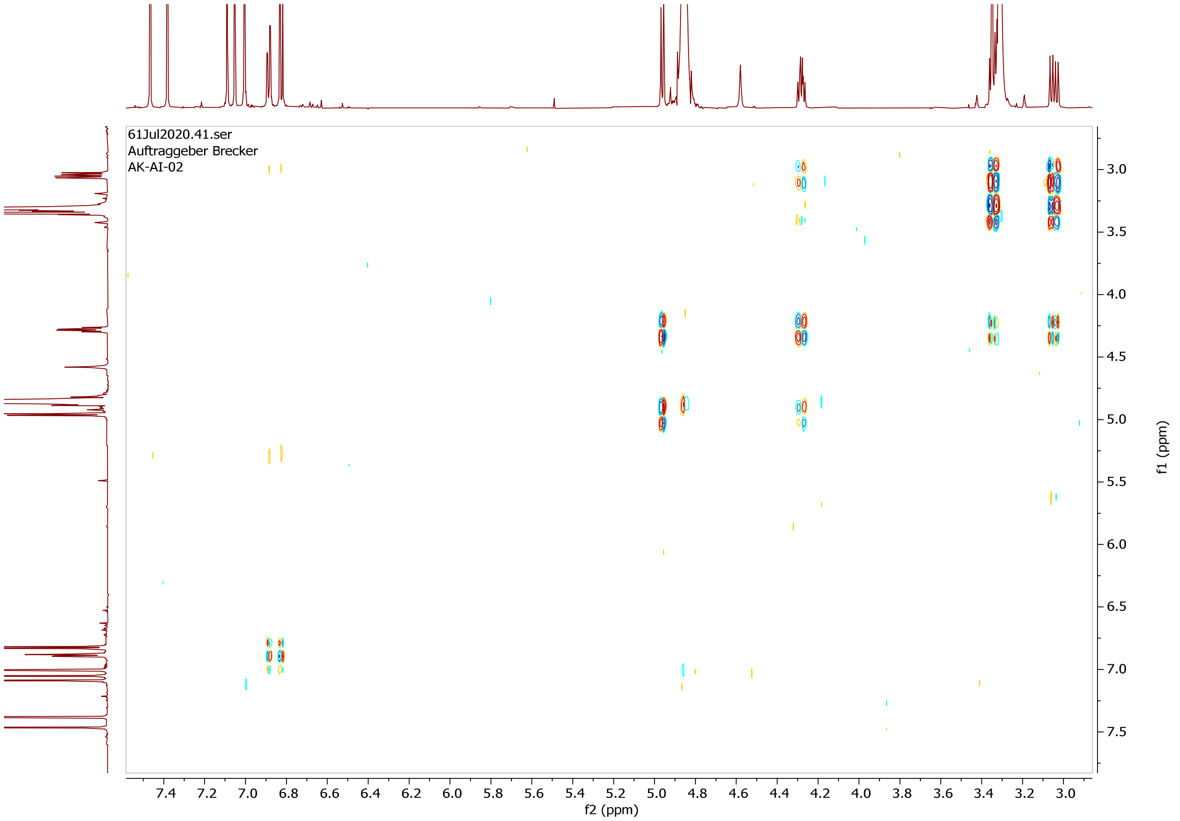

Figure S19. COSY of artocarpinol B (**3**) in CD_3OD.

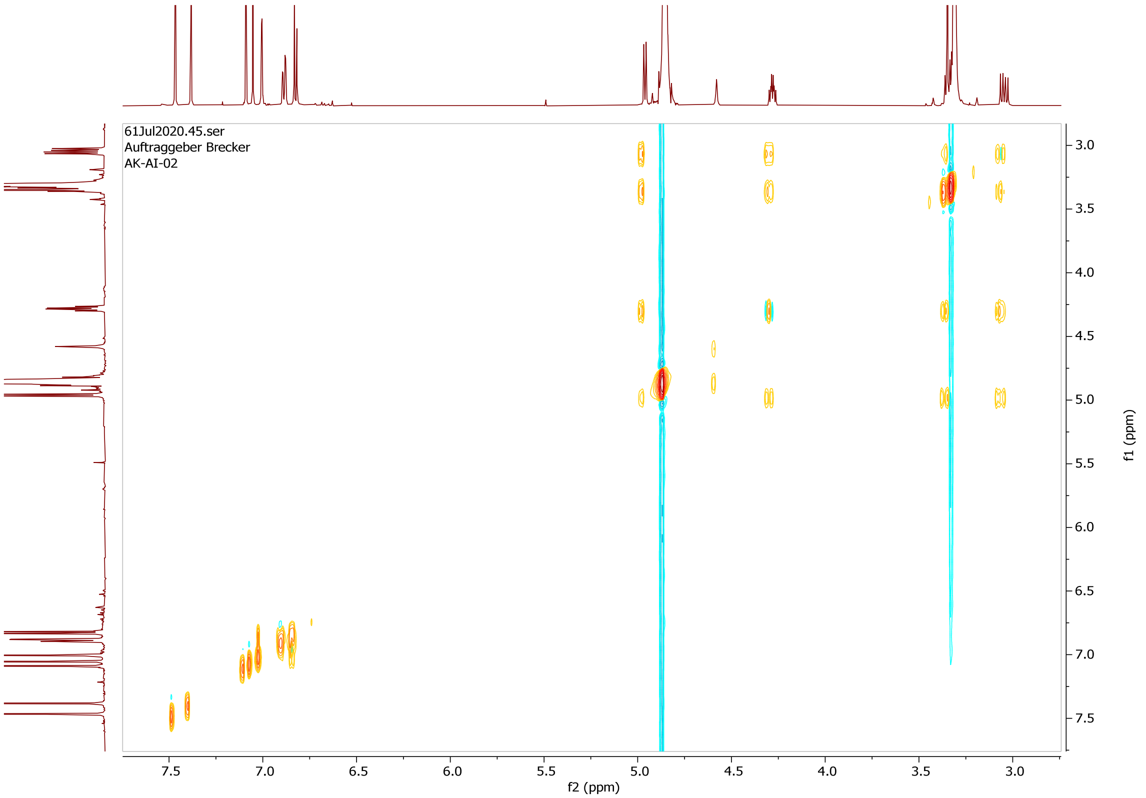

Figure S20. TOCSY of artocarpinol B (**3**) in CD_3OD.

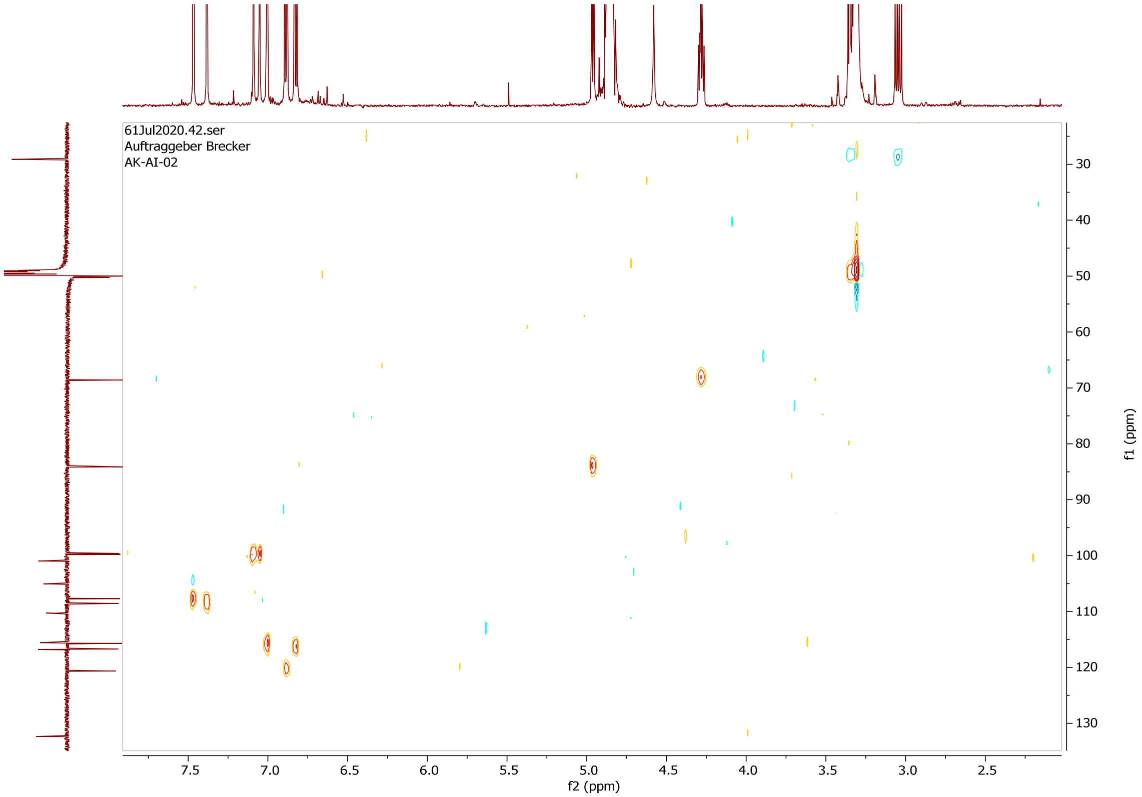

Figure S21. HSQC of artocarpinol B (**3**) in CD_3OD.

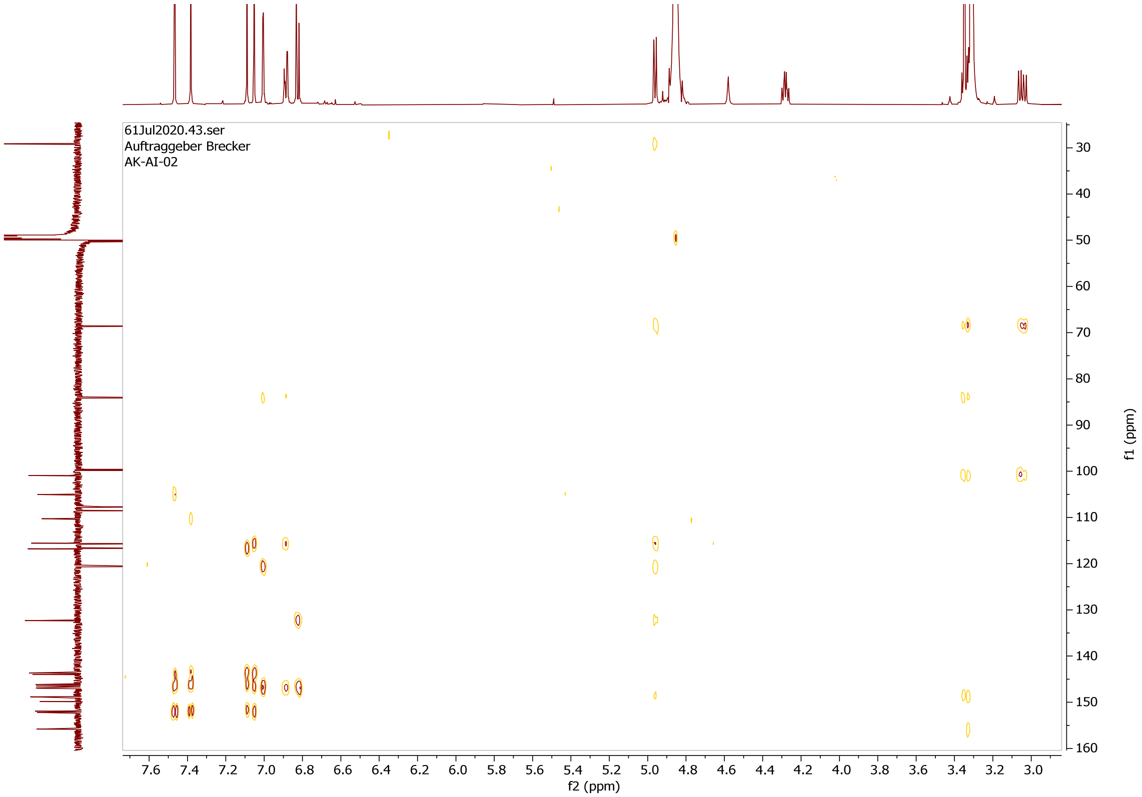

Figure S22. HMBC of artocarpinol B (**3**) in CD_3OD.

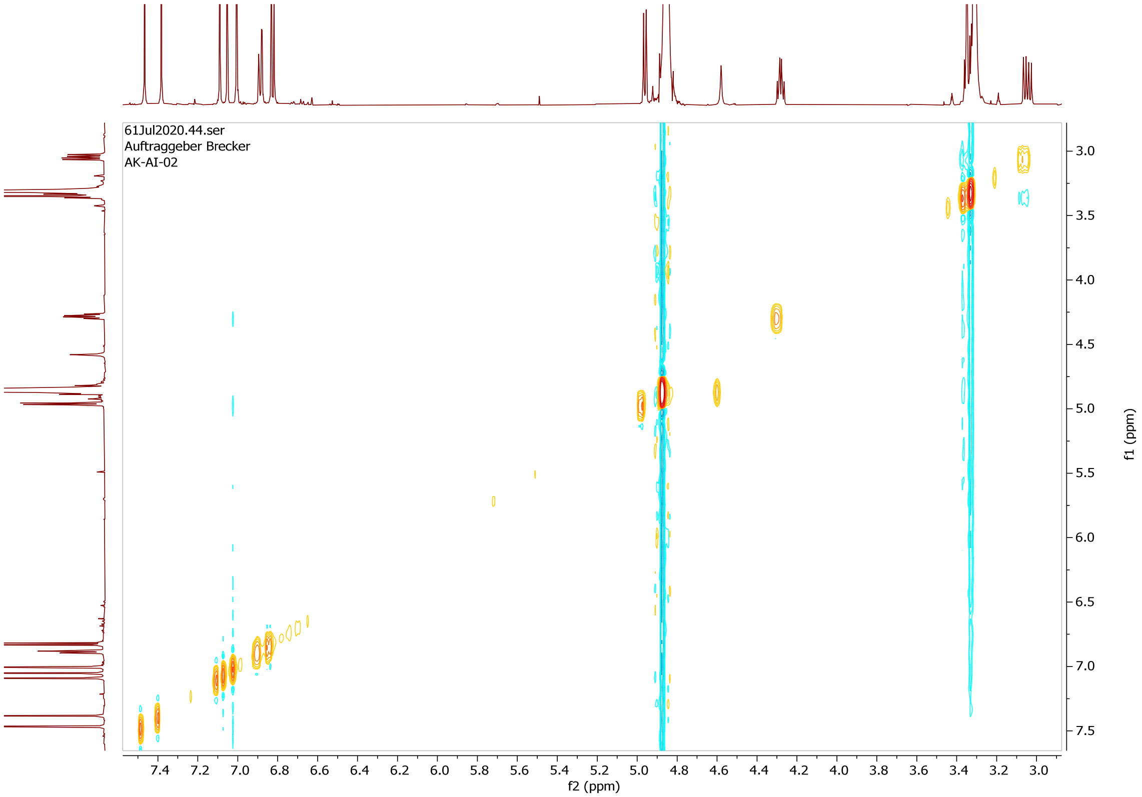

Figure S23. NOESY of artocarpinol B (**3**) in CD_3OD.

Generic Display Report

Analysis Info

Analysis Name	E:\Data\MS_MessService\71761000002.d	Acquisition Date	7/21/2020 8:45:36 AM
Method	tune_low_MS_Service_07_20.m	Operator	msc
Sample Name	AK-AI02	Instrument	maXis
Comment	Karbalei / Brecker<br>ACN/MeOH + 1 % H2O<br>Ergebnis +/- 5 ppm		

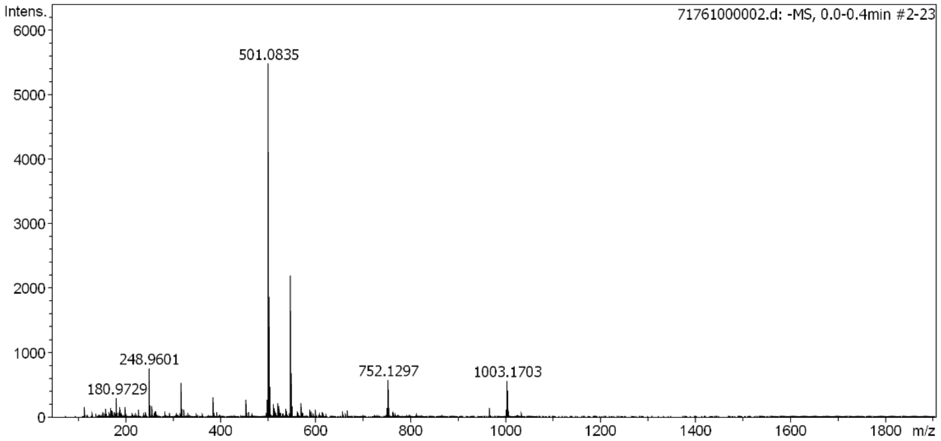

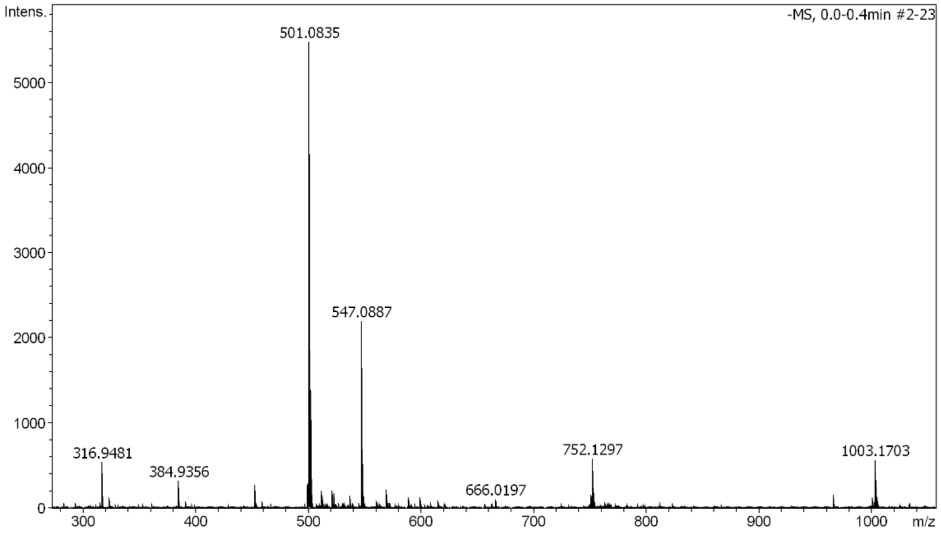

Bruker Compass DataAnalysis 4.1 printed: 7/21/2020 8:46:59 AM by: msc Page 1 of 1

Figure S24. Mass spectrum of artocarpinol B (**3**).

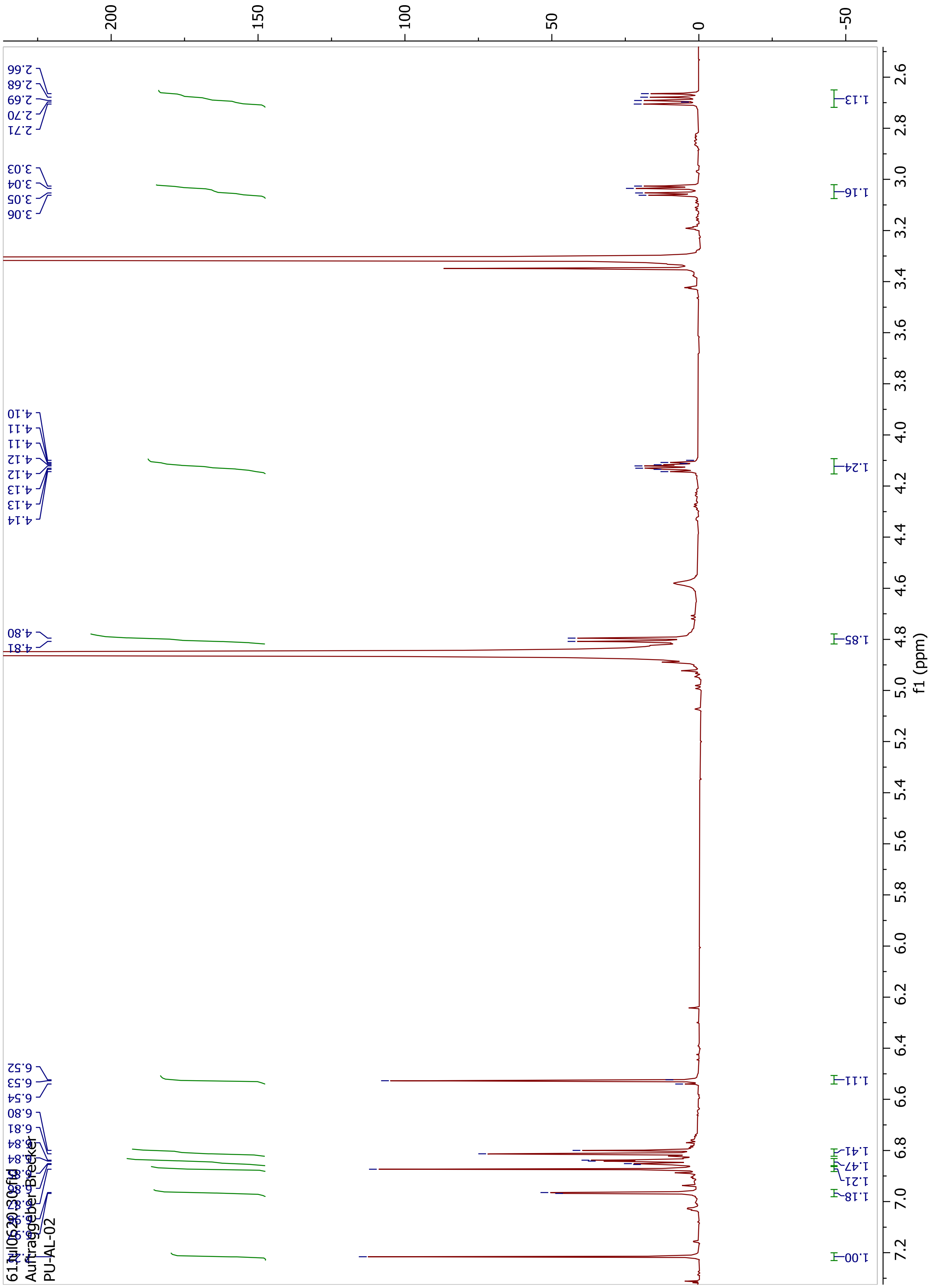

Figure S25. ^{1}H NMR of gambircatechol (**4**) in CD_3OD.

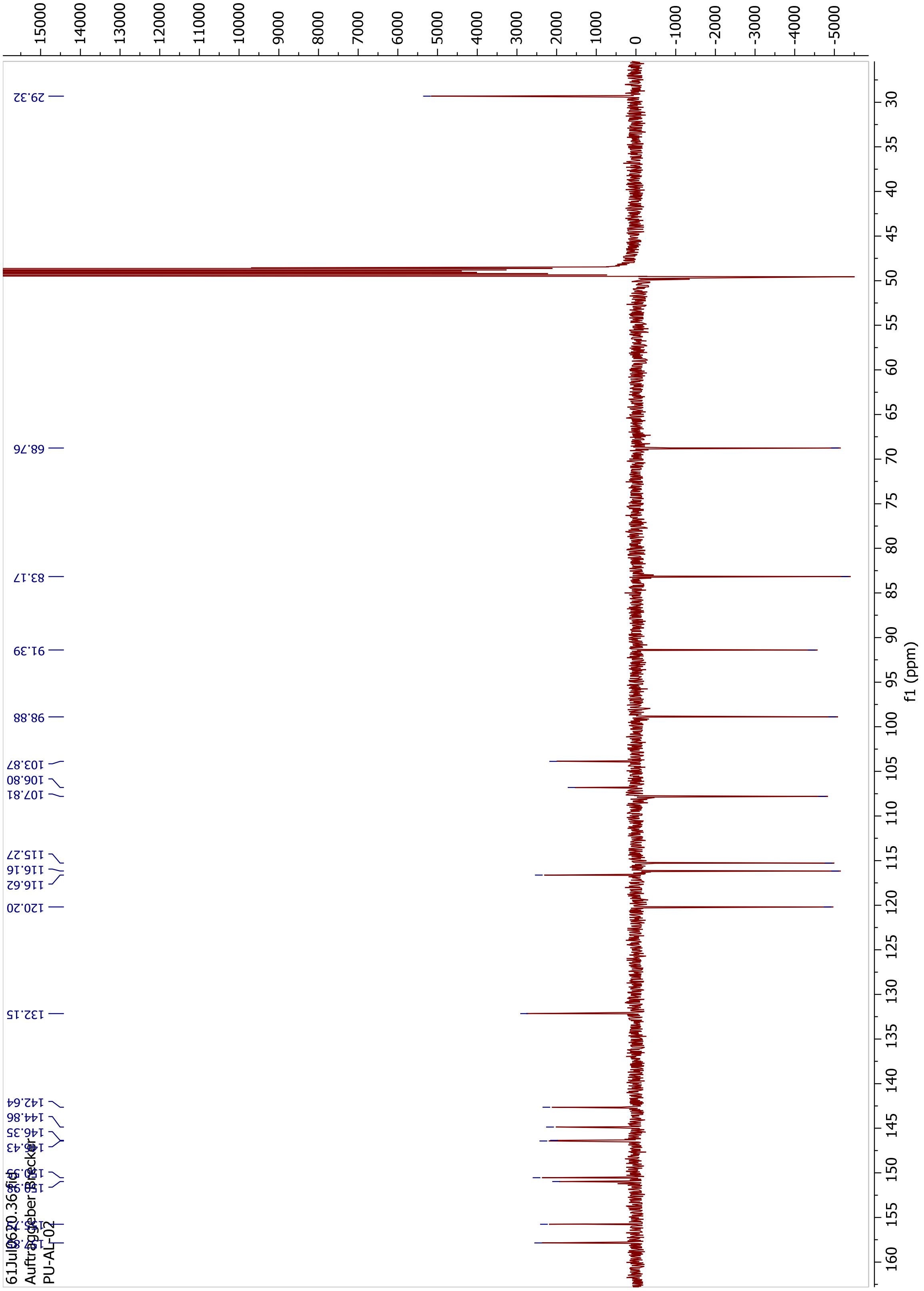

Figure S26. ^{13}C NMR of gambircatechol (**4**) in CD_3OD.

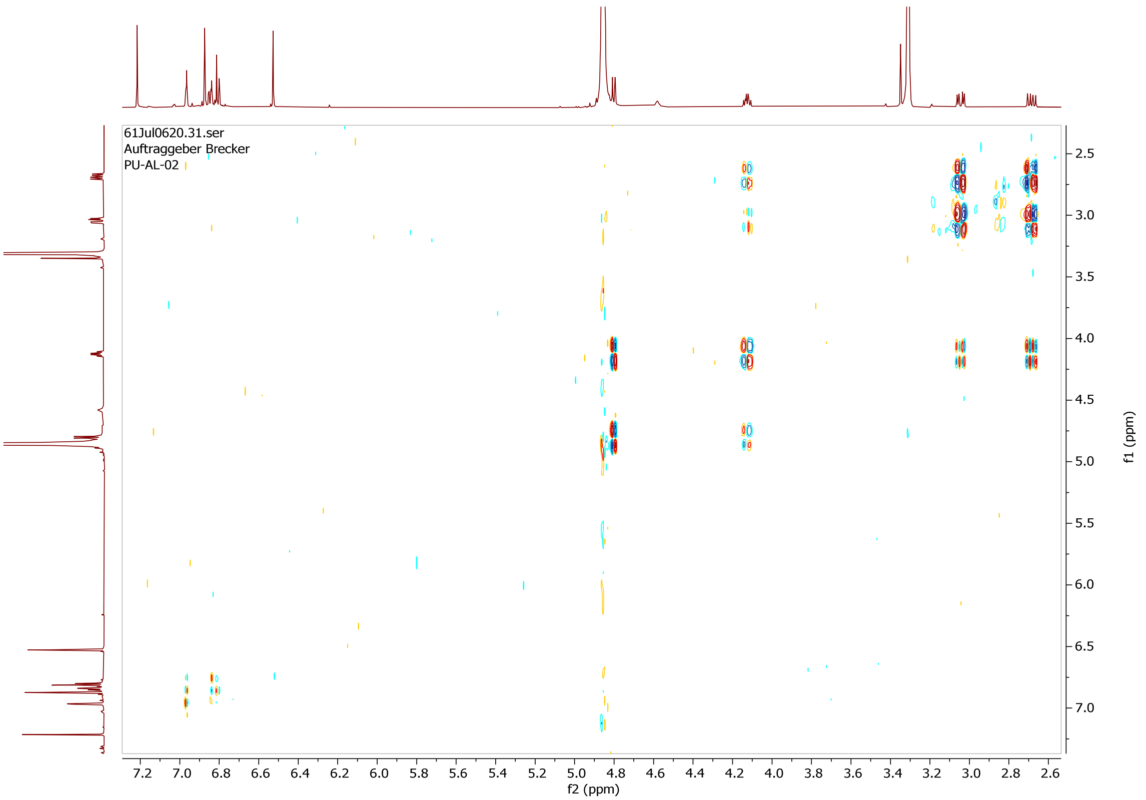

Figure S27. COSY of gambircatechol (**4**) in CD_3OD.

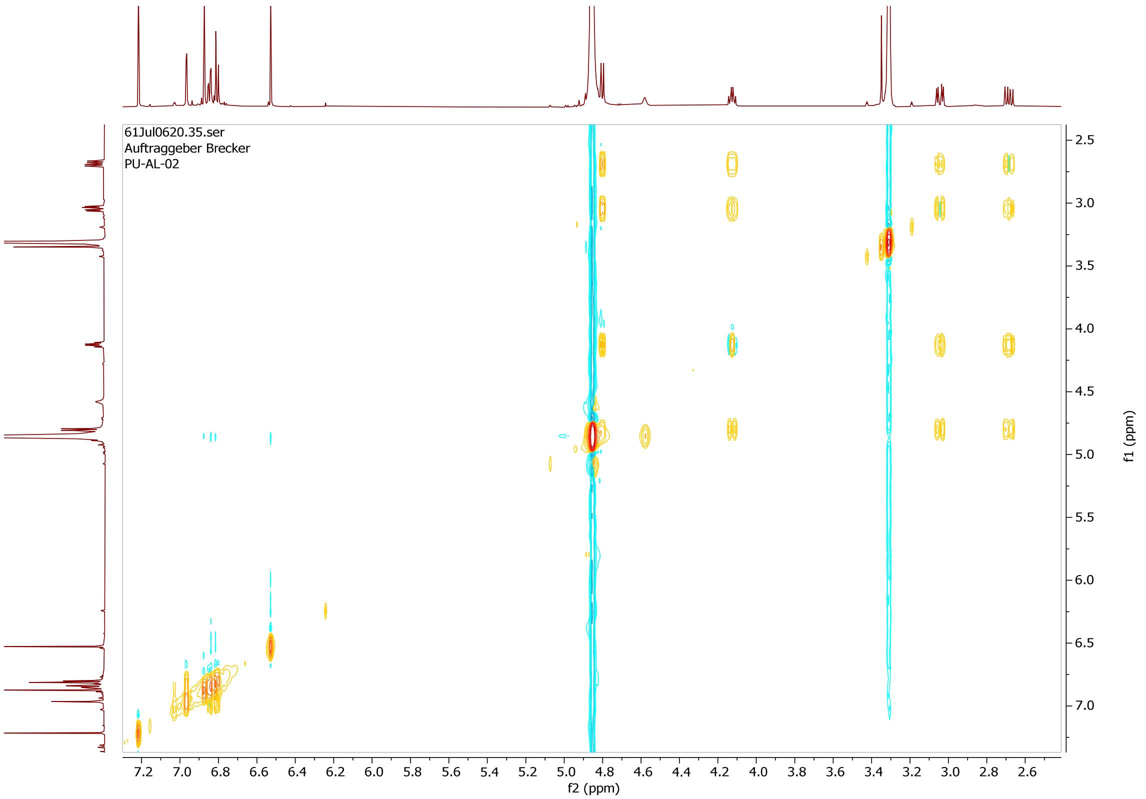

Figure S28. TOCSY of gambircatechol (**4**) in CD_3OD.

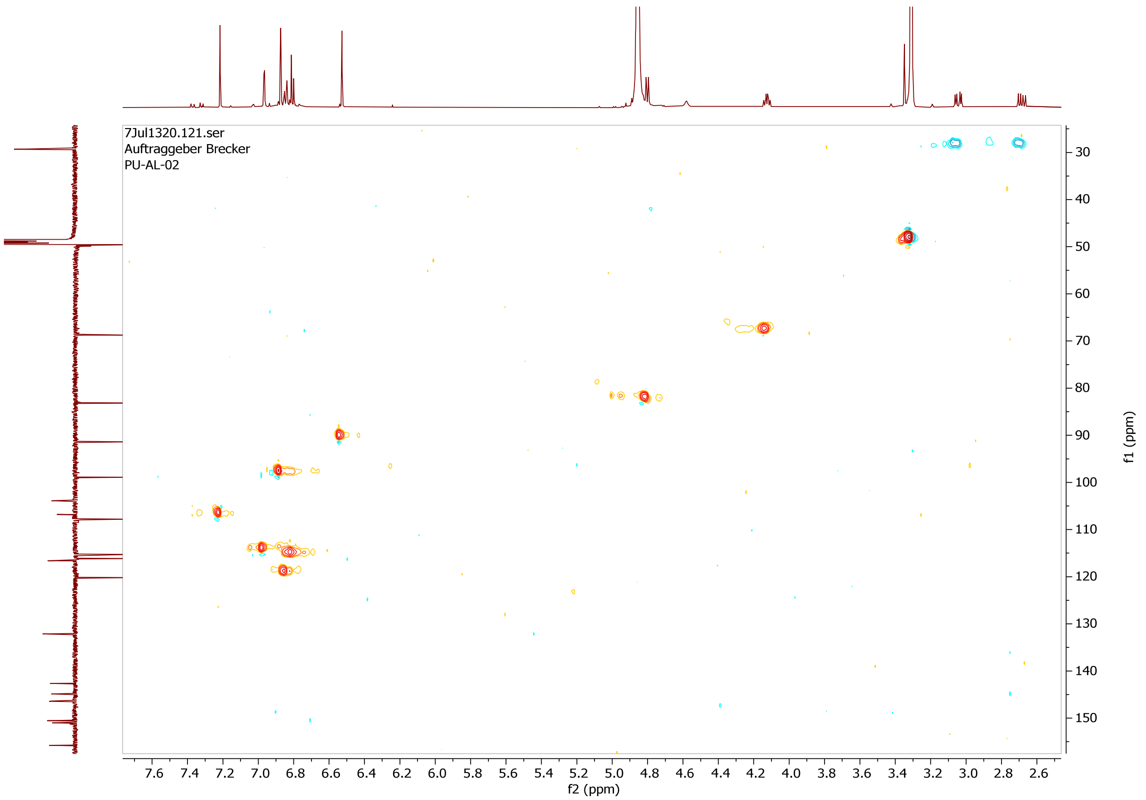

Figure S29. HSQC of gambircatechol (**4**) in CD_3OD.

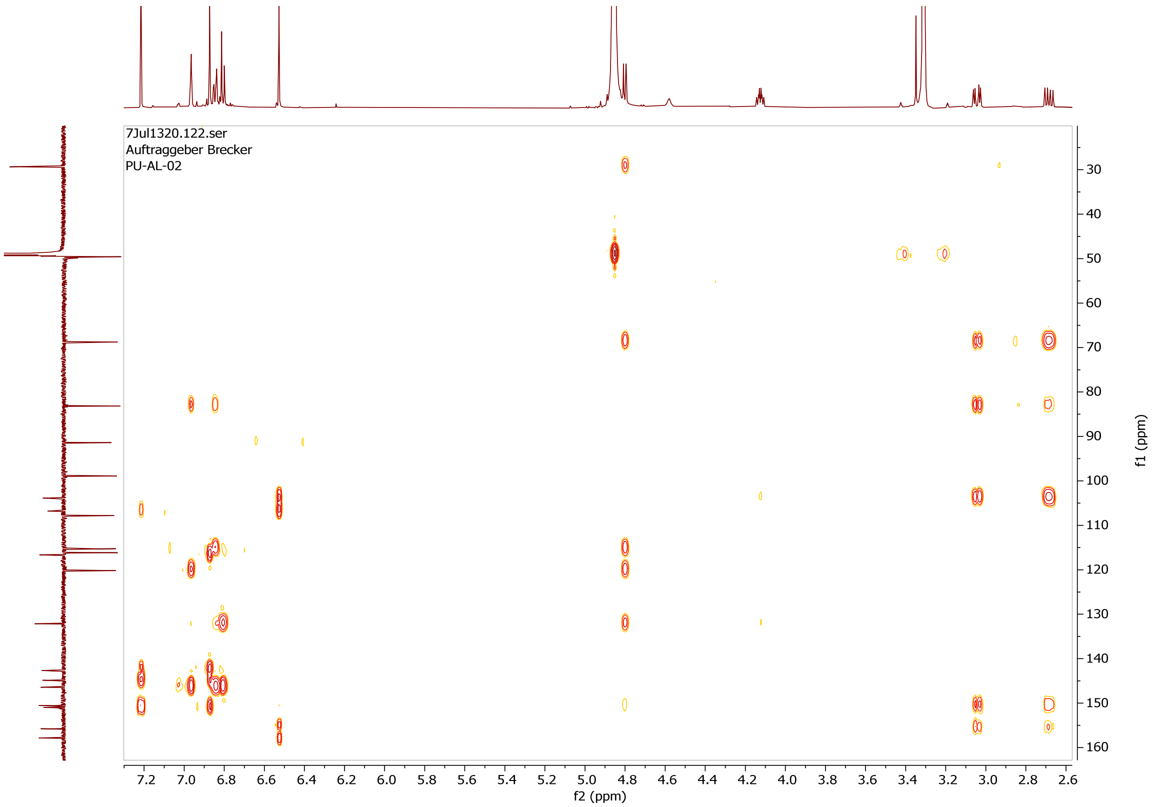

Figure S30. HMBC of gambircatechol (**4**) in CD_3OD.

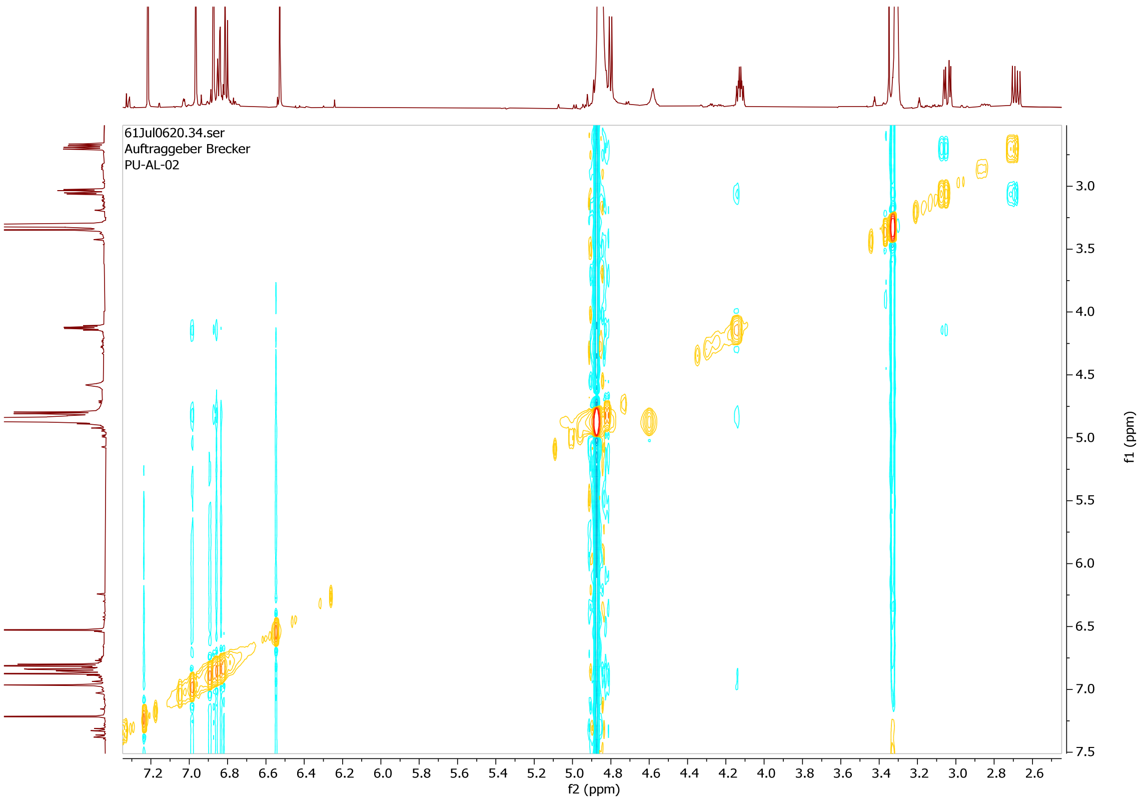

Figure S31. NOESY of gambircatechol (**4**) in CD_3OD.

Generic Display Report

Analysis Info

Analysis Name	E:\Data\MS_MessService\71363000002.d	Acquisition Date	7/2/2020 4:57:44 PM
Method	tune_low_MS_Service_06_20.m	Operator	msc
Sample Name	PU-AL02	Instrument	maXis
Comment	Urban/Brecker/Botanik<br>Ergebnis +/- 5 ppm<br>ACN/MeOH + 1% H2O		

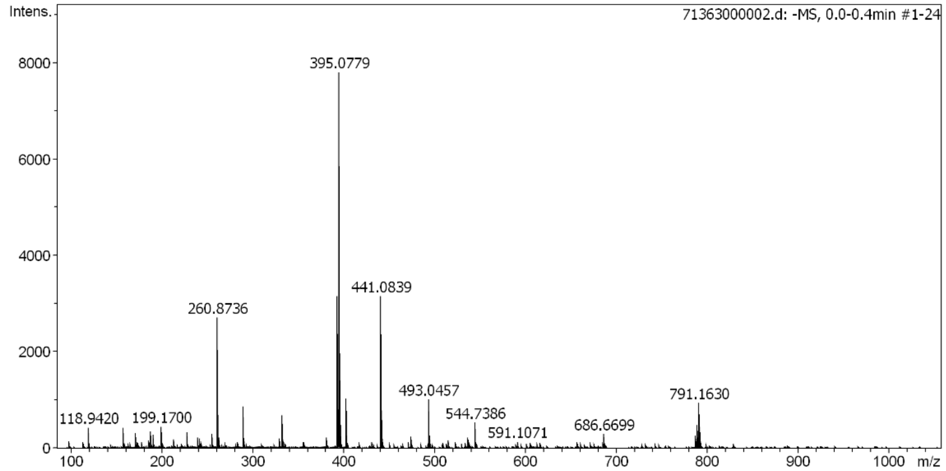

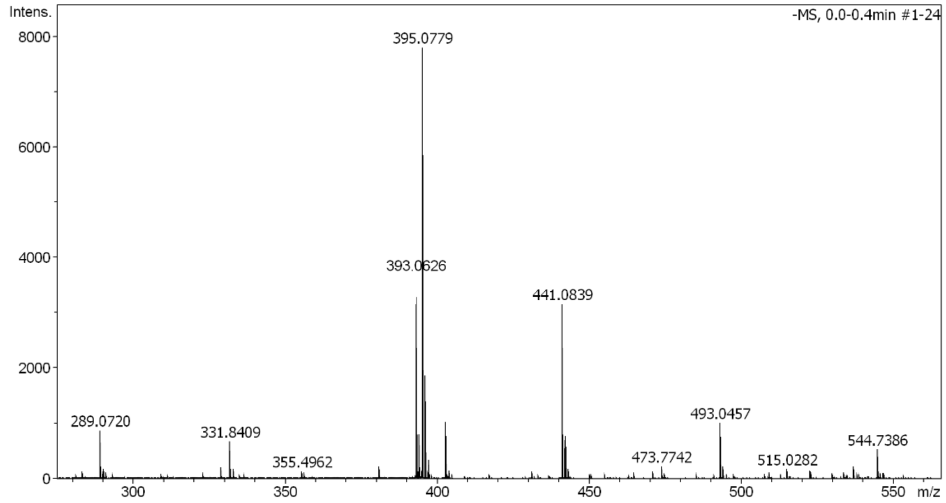

Bruker Compass DataAnalysis 4.1 printed: 7/2/2020 5:32:36 PM by: msc Page 1 of 1

Figure S32. Mass spectrum of gambircatechol (**4**).

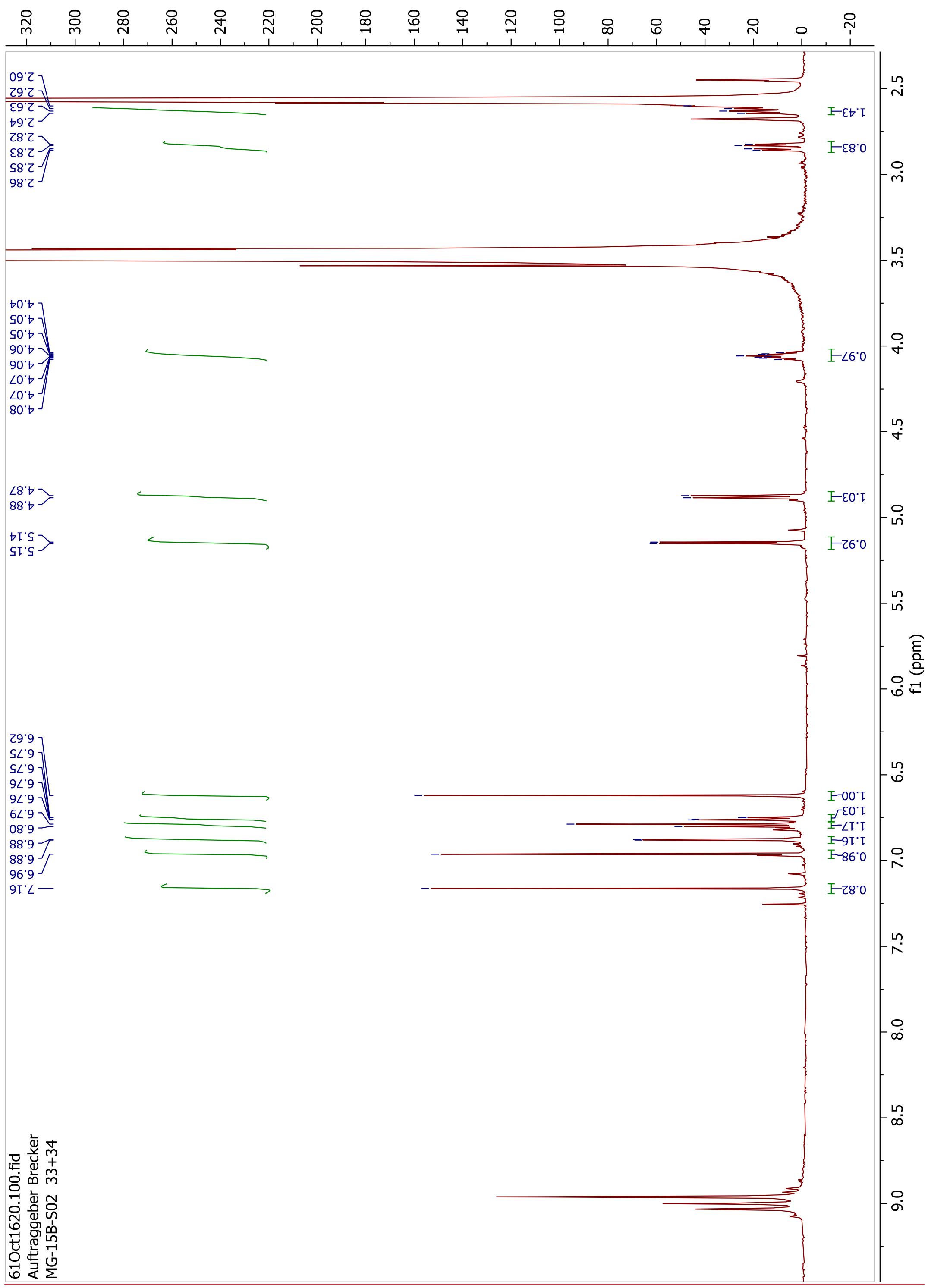

Figure S33. ^{1}H NMR of *ortho*-quinone of **4** in DMSO-d_6.

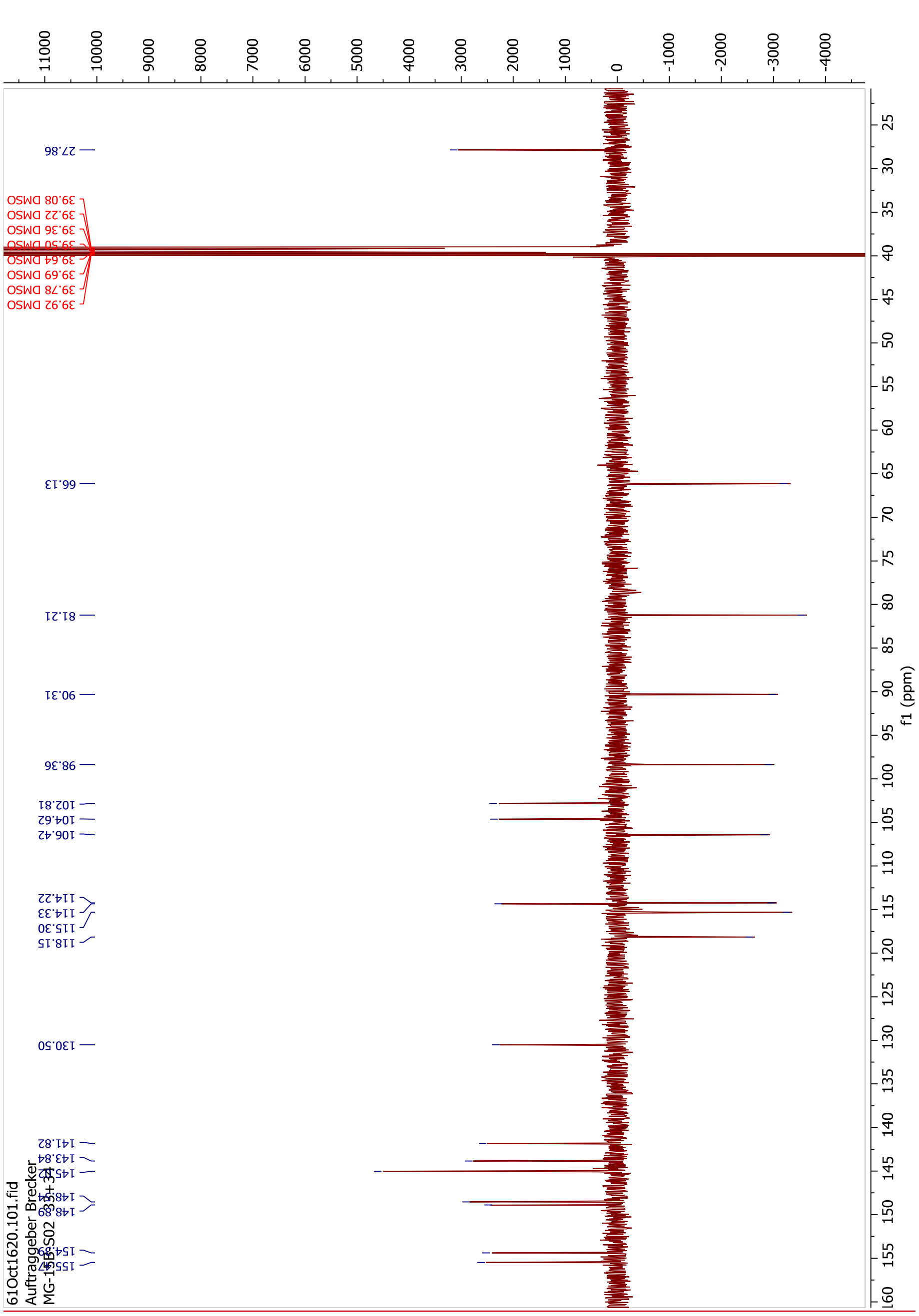

Figure S34. ^{13}C NMR of *ortho*-quinone of **4** in DMSO-*d*$_6$.

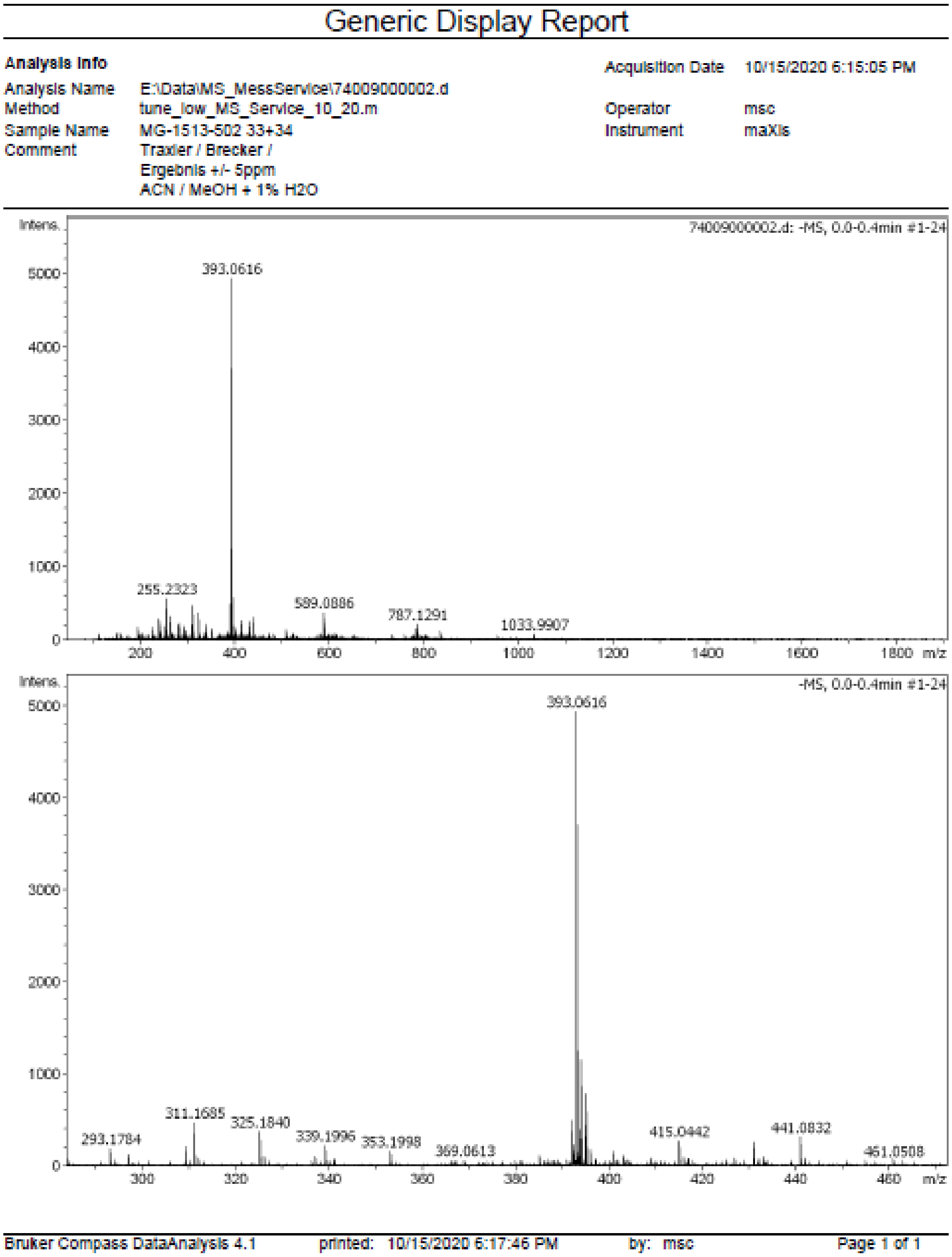

Figure S35. Mass spectrum of *ortho*-quinone of **4**.

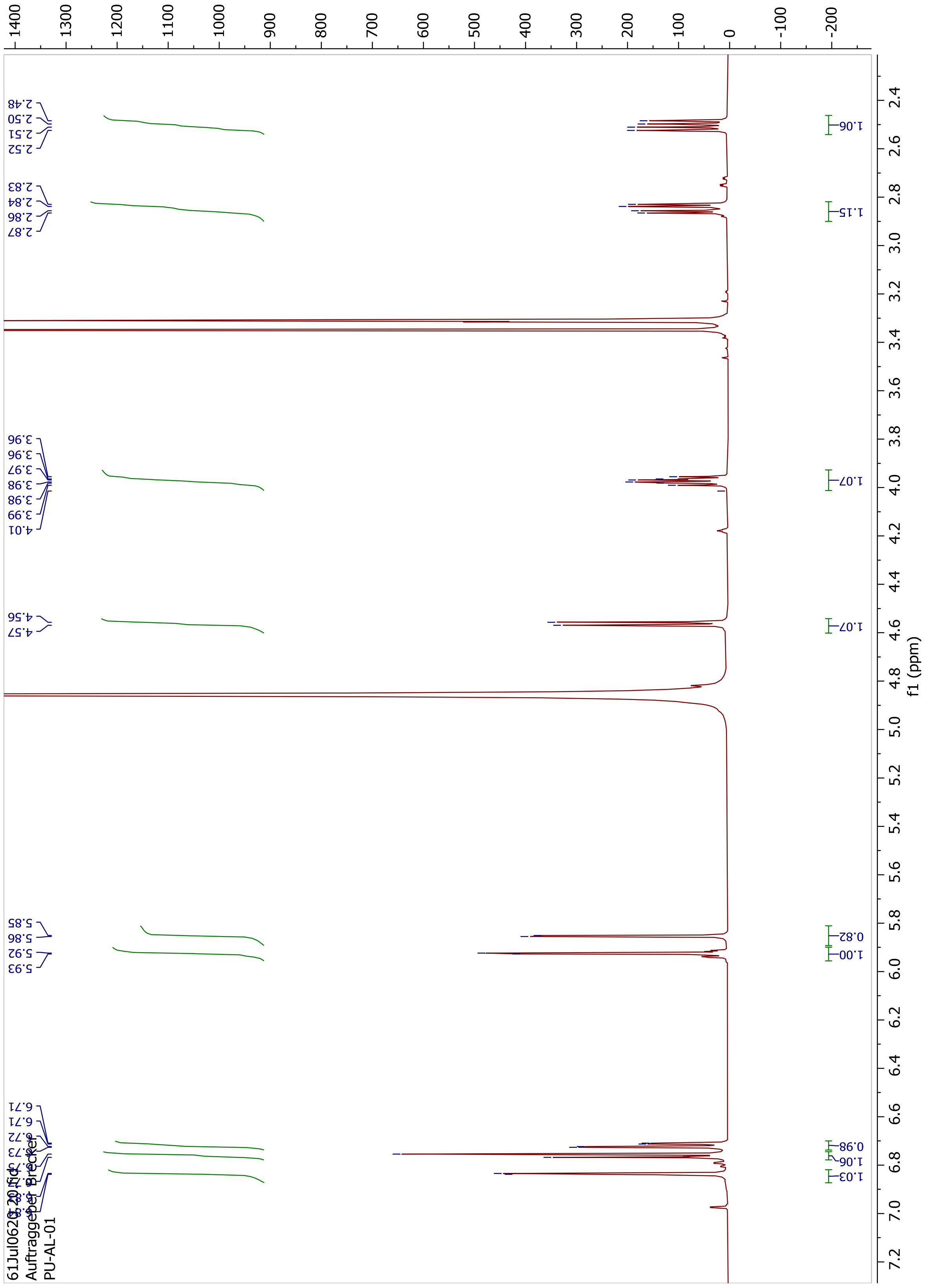

Figure S36. ^{1}H NMR of (+)-catechin (**5**) in CD_3OD.

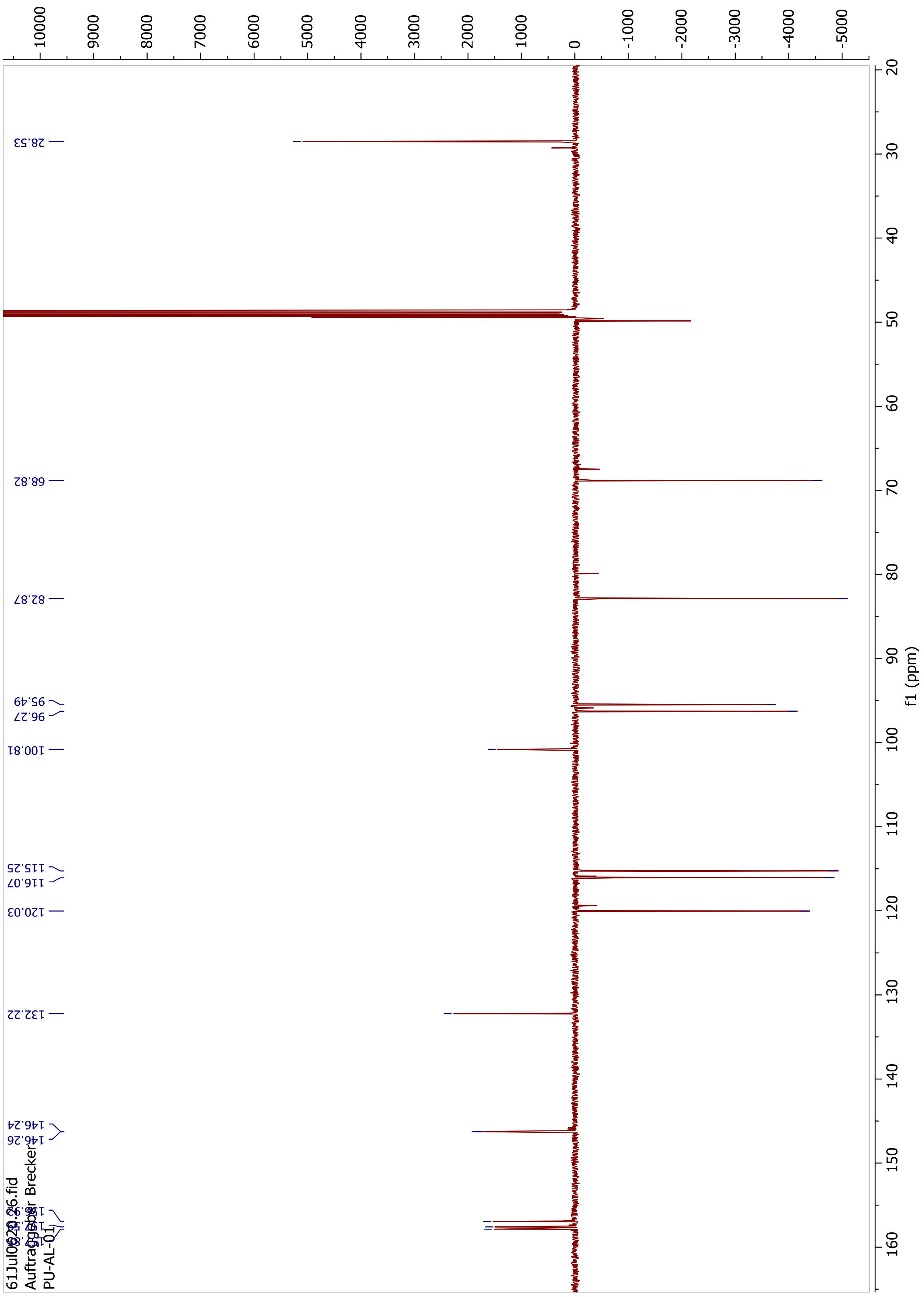

Figure S37. ^{13}C NMR of (+)-catechin (**5**) in CD_3OD.

Generic Display Report

Analysis Info

Analysis Name	E:\Data\MS_MessService\71362000002.d	Acquisition Date	7/2/2020 4:56:39 PM
Method	tune_low_MS_Service_06_20.m	Operator	msc
Sample Name	PU-AL01	Instrument	maXis
Comment	Urban/Brecker/Botanik<br>Ergebnis +/- 5 ppm<br>ACN/MeOH + 1% H2O		

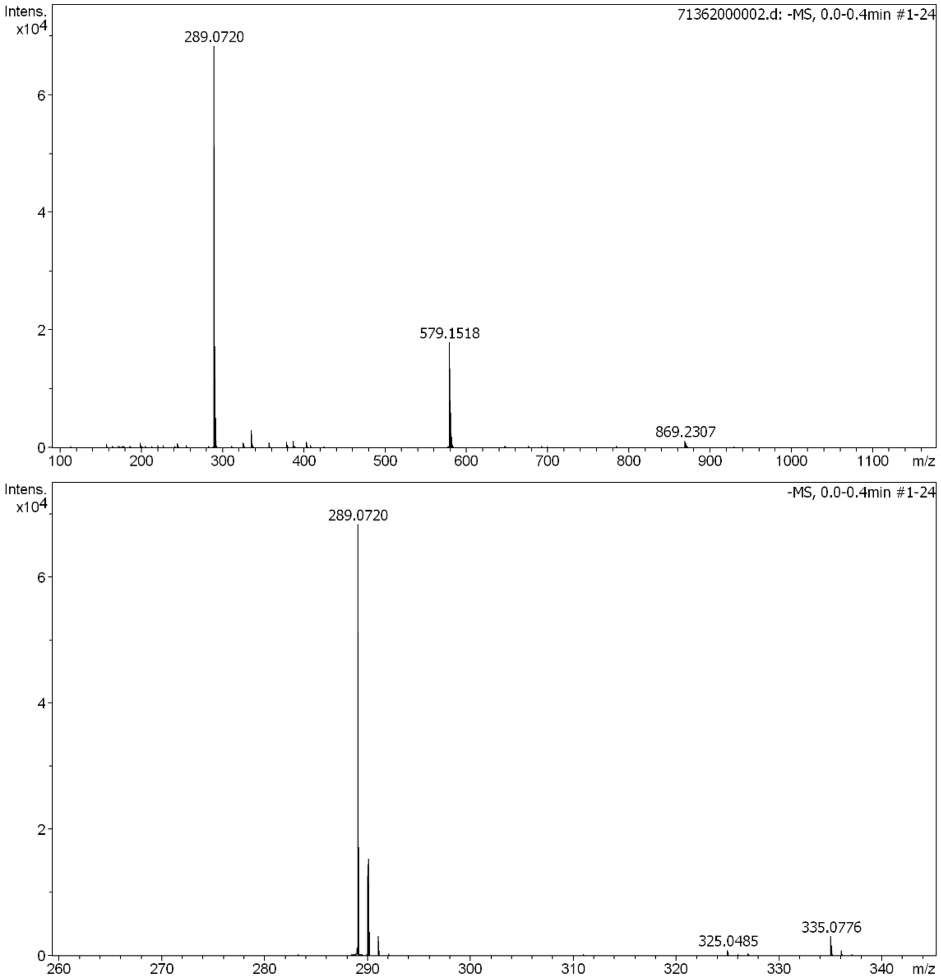

Bruker Compass DataAnalysis 4.1 printed: 7/2/2020 5:25:34 PM by: msc Page 1 of 1

Figure S38. Mass spectrum of (+)-catechin (**5**).

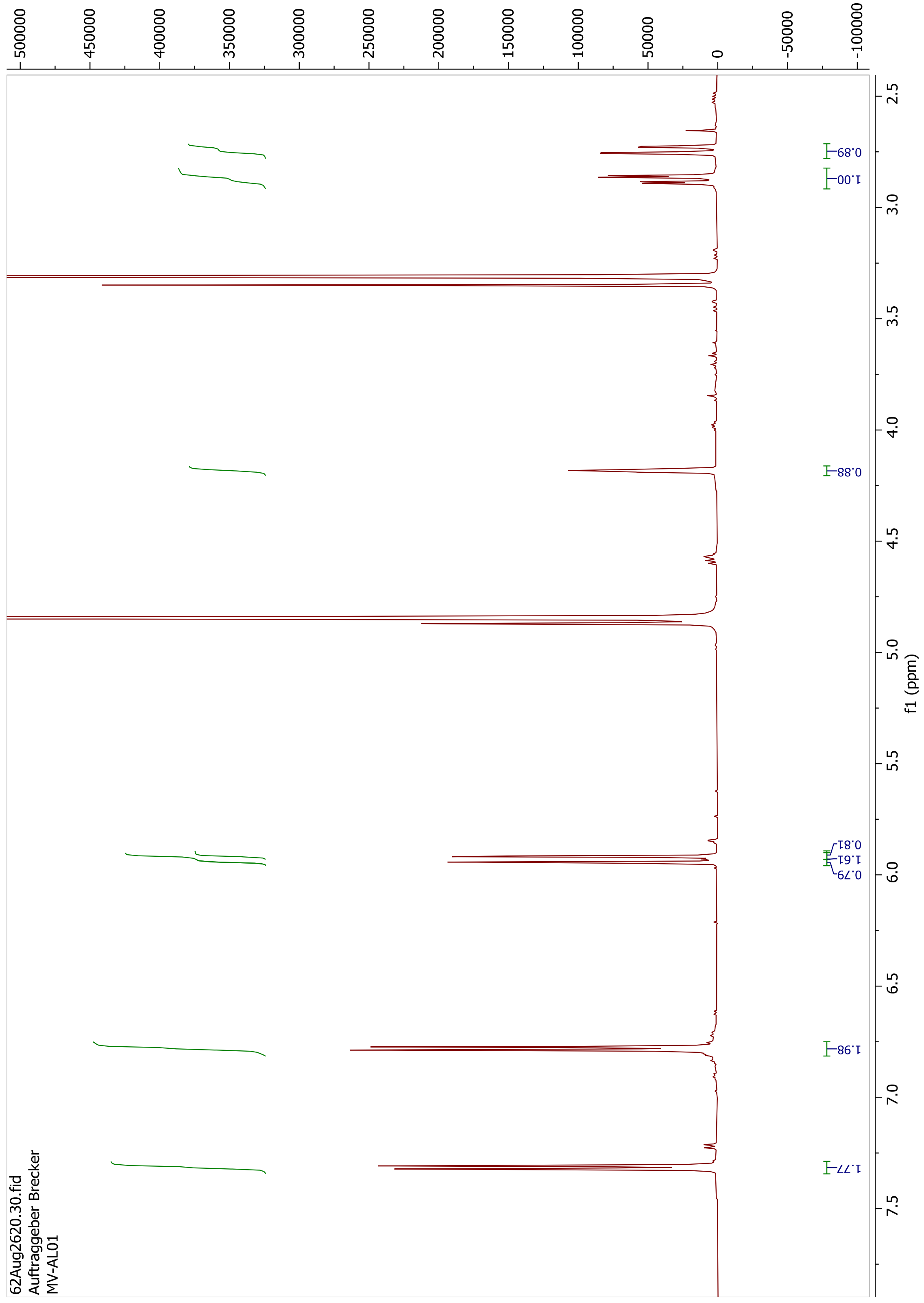

Figure S39. ^{1}H NMR of (+)-afzelechin (**6**) in CD_3OD.

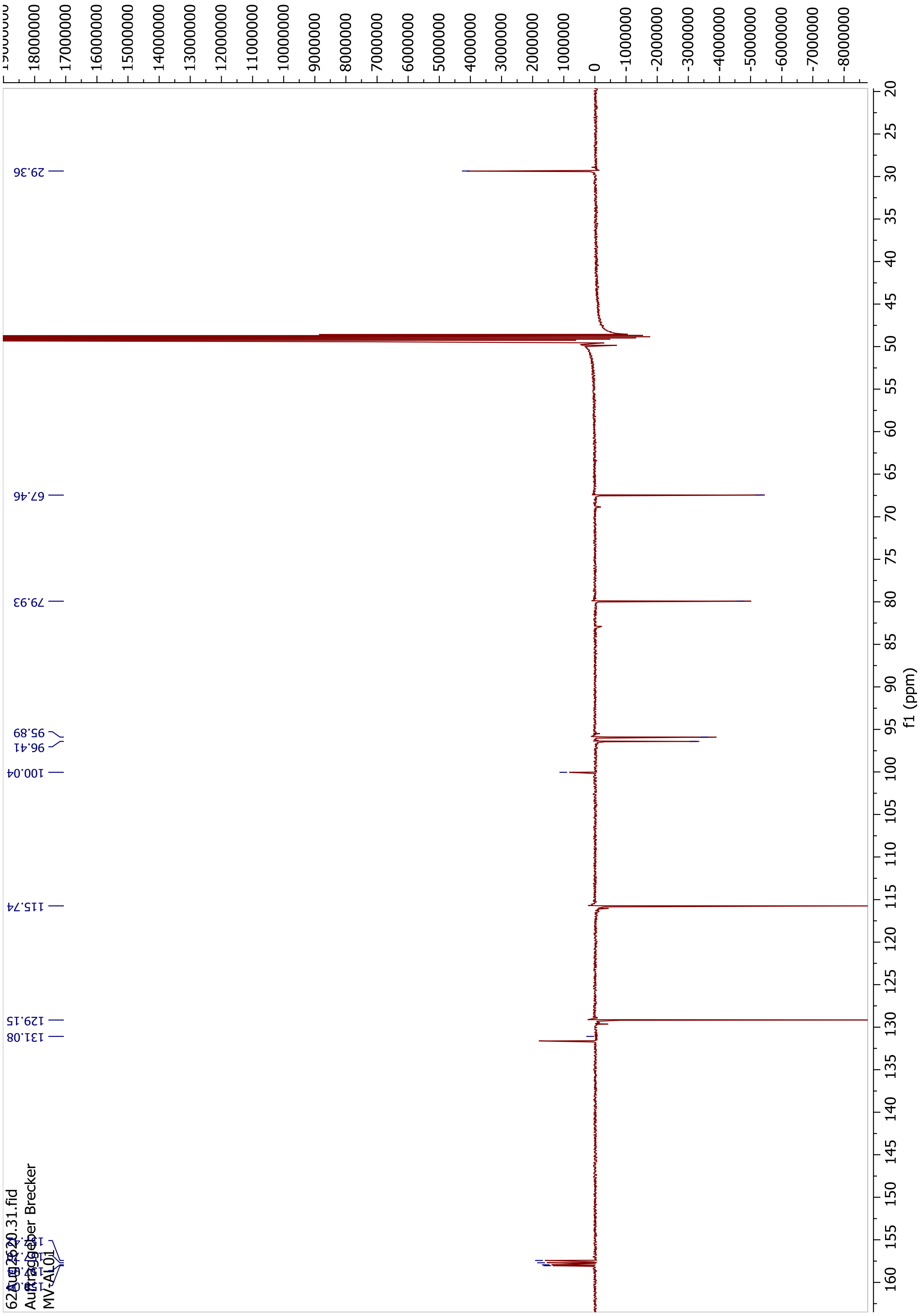

Figure S40. ^{13}C NMR of (+)-afzelechin (**6**) in CD_3OD.

Generic Display Report

Analysis Info

Analysis Name	E:\Data\MS_MessService\72662000002.d	Acquisition Date	8/26/2020 11:30:32 AM
Method	tune_low_MS_Service_08_20.m	Operator	msc
Sample Name	MV-AL01	Instrument	maXis
Comment	Völkl/Brecker<br>Ergebnis +/- 5ppm<br>ACN / MeOH + 1% H2O		

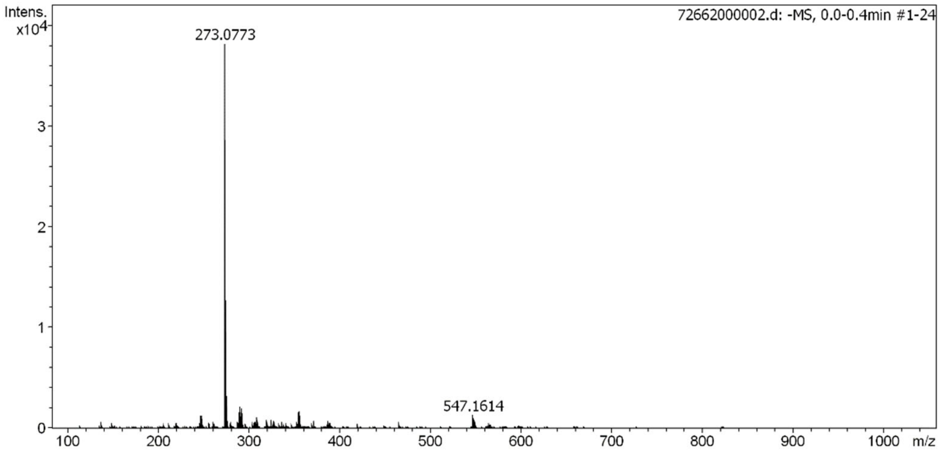

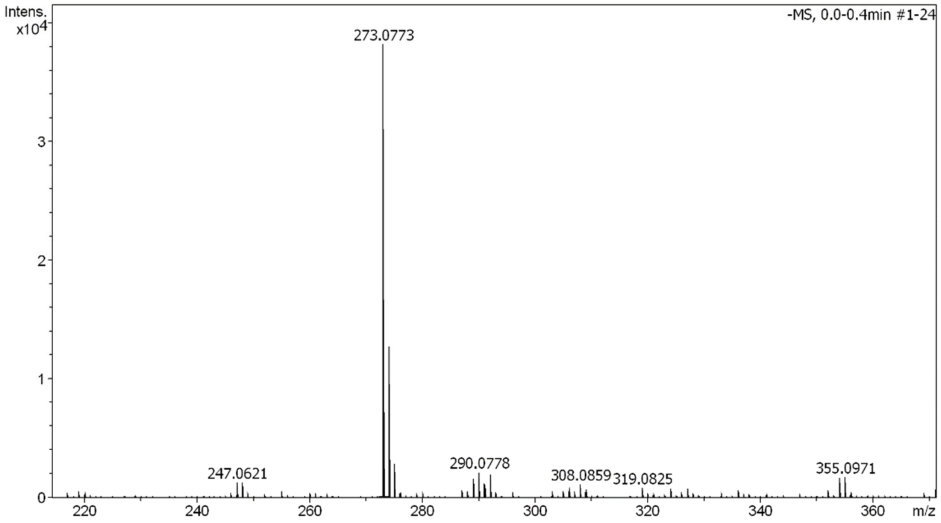

Bruker Compass DataAnalysis 4.1 printed: 8/26/2020 11:35:35 AM by: msc Page 1 of 1

Figure S41. Mass spectrum of (+)-afzelechin (**6**).

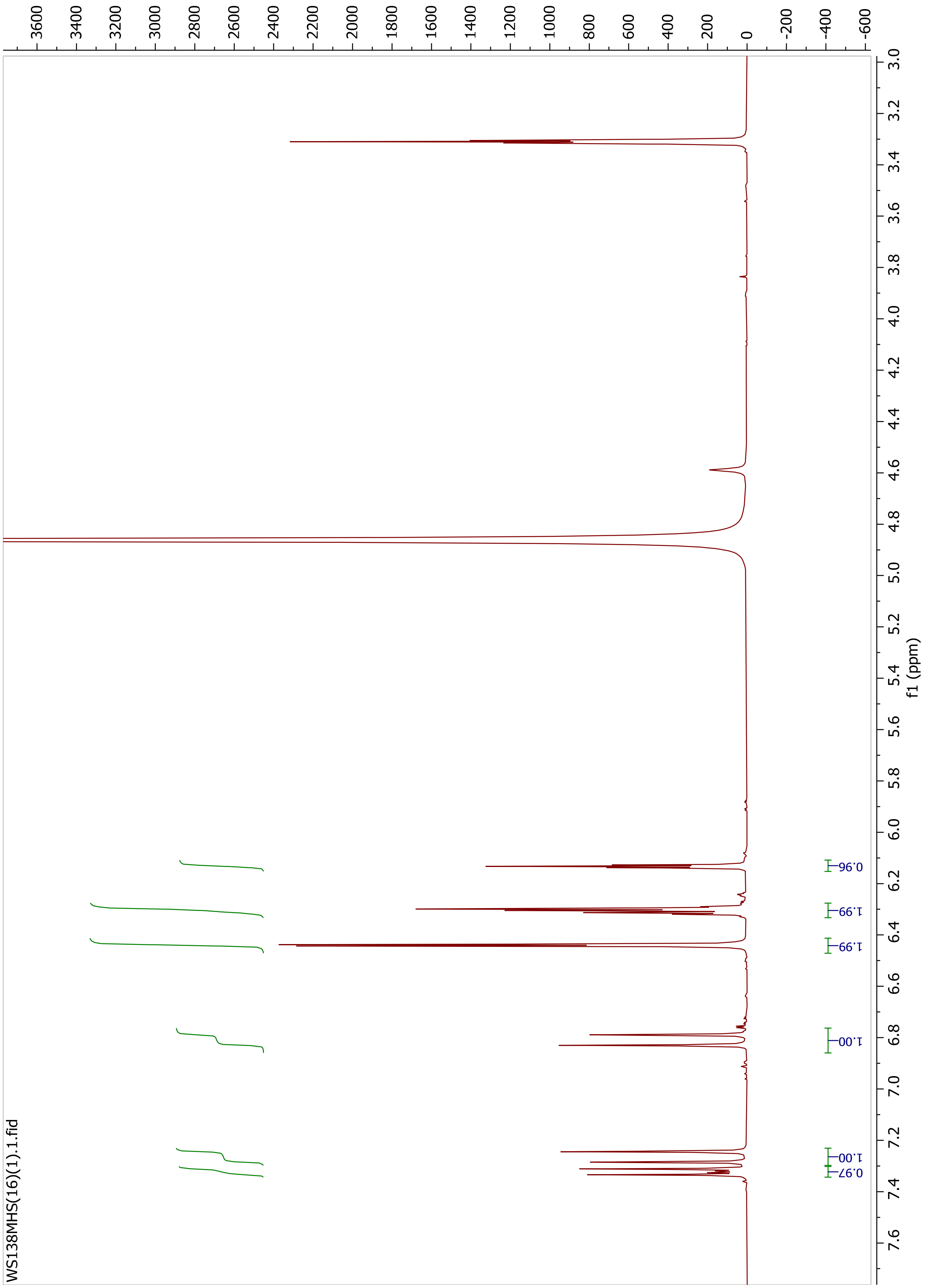

Figure S42. ^{1}H NMR of oxyresveratrol (**7**) in CD_3OD.

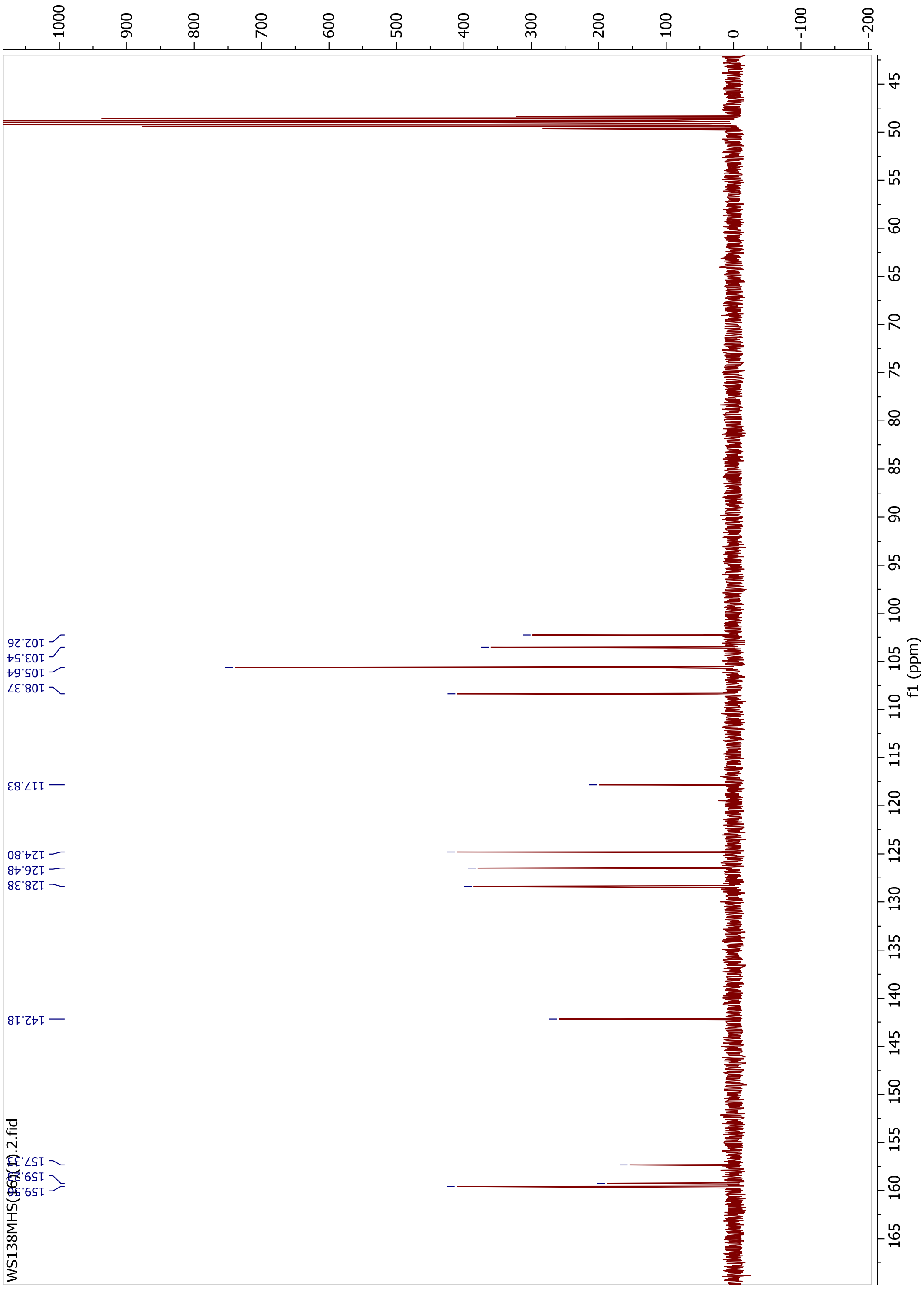

Figure S43. ^{13}C NMR of oxyresveratrol (**7**) in CD_3OD.

Generic Display Report

Analysis Info		Acquisition Date	9/17/2020 3:48:50 PM
Analysis Name	E:\Data\MS_MessService\73241000001.d		
Method	tune_low_MS_Service_09_20.m	Operator	msc
Sample Name	Oxyresveratrol	Instrument	maXis
Comment	Weerasak Songoen / Brecker<br>Ergebnis +/- 5ppm<br>ACN/MeOH + 1 % H2O		

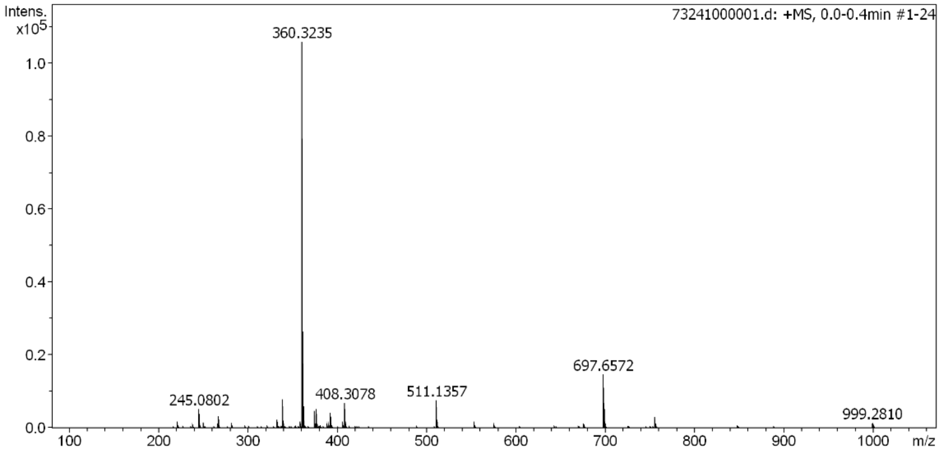

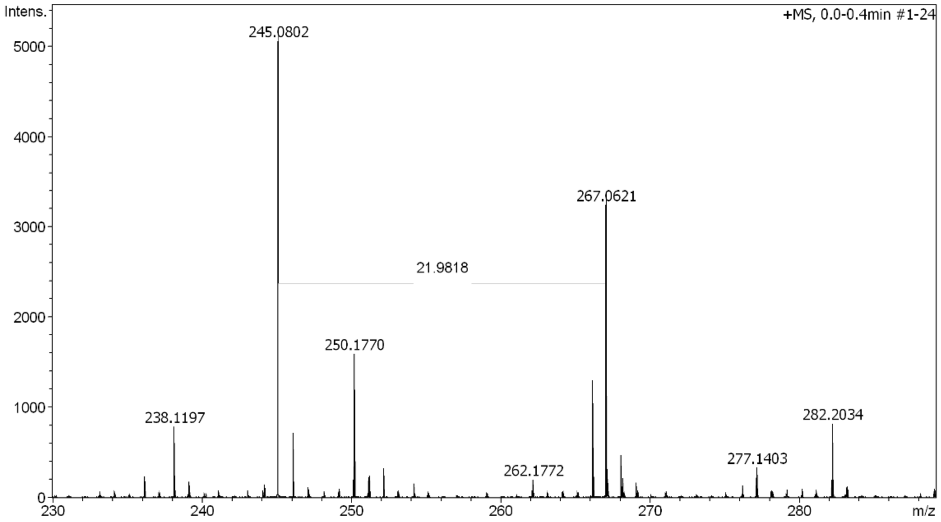

Bruker Compass DataAnalysis 4.1 printed: 9/17/2020 6:01:00 PM by: msc Page 1 of 1

Figure S44. Mass spectrum of oxyresveratrol (**7**).

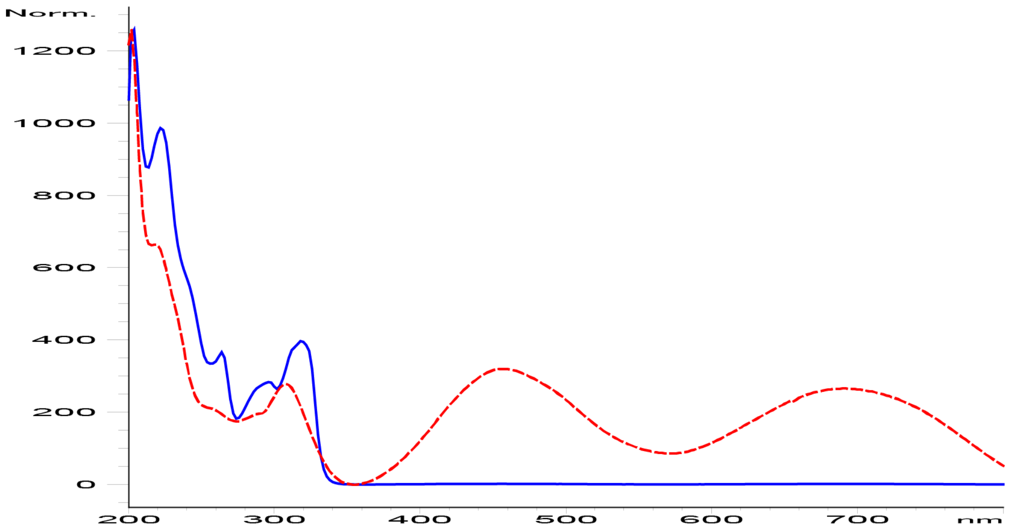

Figure S45. UV spectra of gambircatechol (**4**; blue) and the *ortho*-quinone (red).

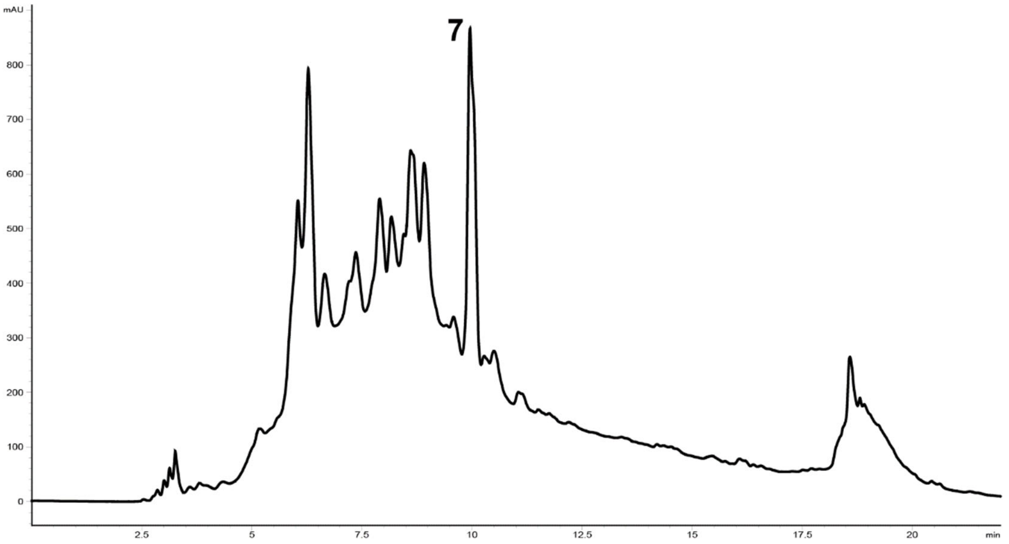

Figure S46. HPLC profile of the stem extract at 230 nm. **7** = oxyresveratrol.

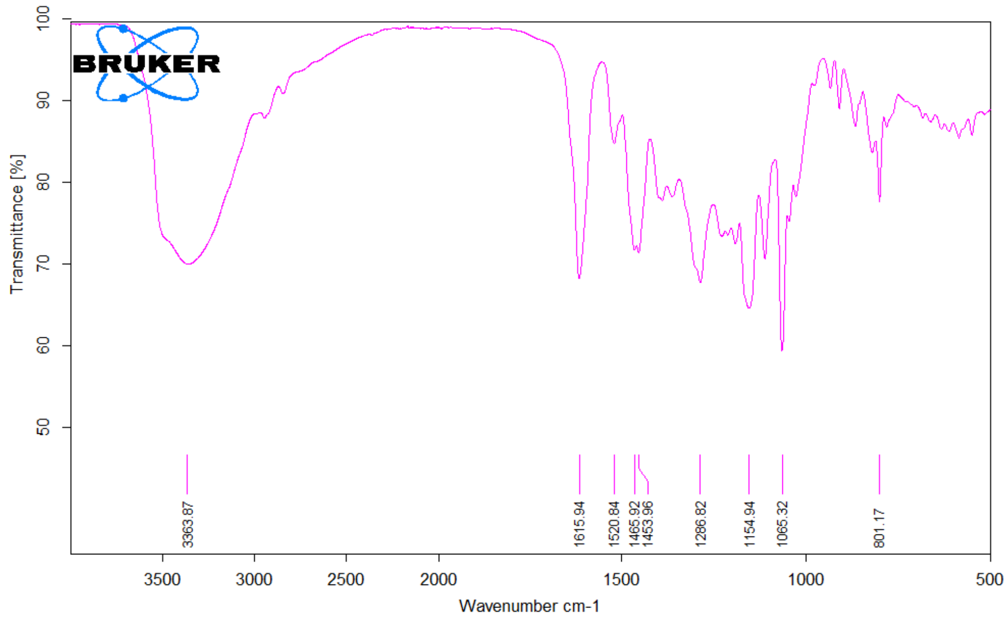

Figure S 47. IR spectrum of artocarpinol A (**1**).

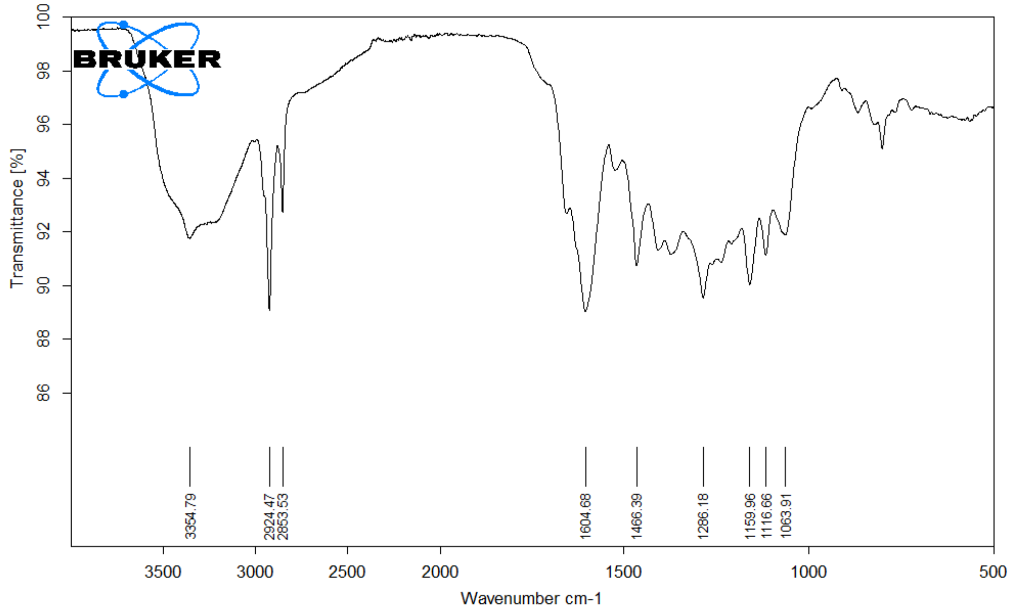

Figure S 48 IR spectrum of 3-*epi*-artocarpinol A (**2**).

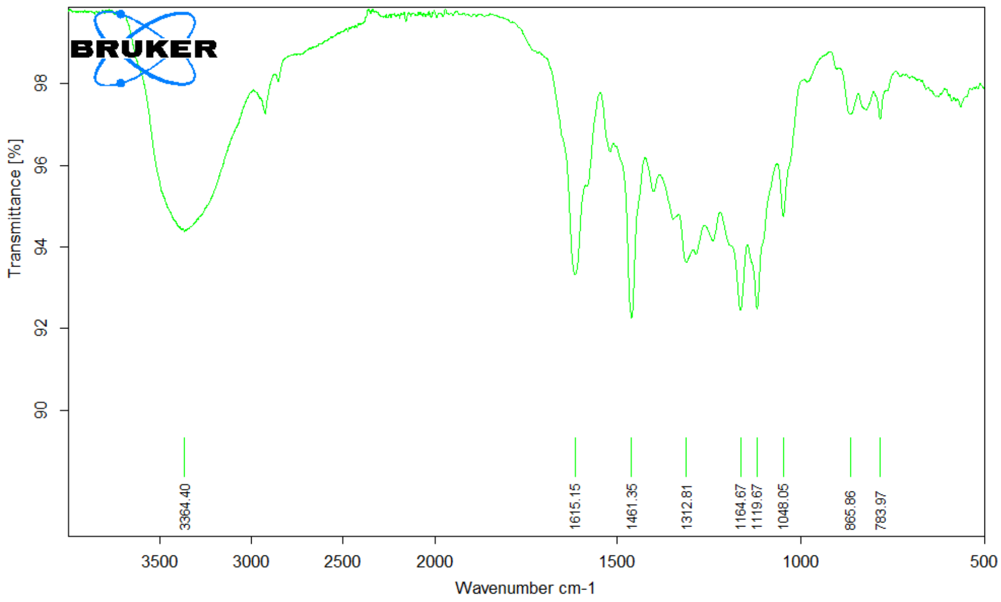

Figure S 49 IR spectrum of artocarpinol B (**3**).

Table S1. NMR spectroscopic data [ppm] of gambircatechol (**4**), (+)-catechin (**5**) and (+)-afzelechin (**6**) recorded in CD_3OD. The relative integral, the multiplicity and the coupling constants [Hz] are provided. In addition, the ^{13}C NMR chemical shifts and multiplicities are given.

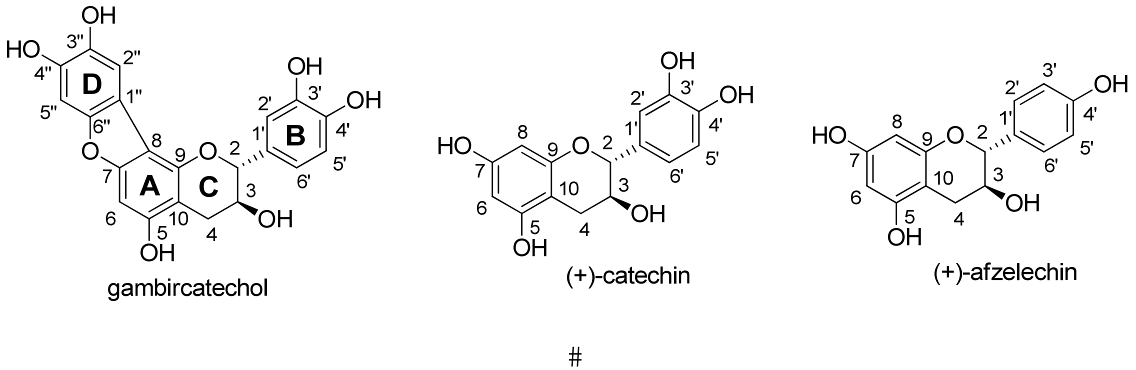

gambircatechol (+)-catechin (+)-afzelechin

#

position	gambircatechol (**4**)		(+)-catechin (**5**)		(+)-afzelechin (**6**)	
	δ_H (*J* in Hz)	δ_C	δ_H (*J* in Hz)	δ_C	δ_H (*J* in Hz)	δ_C
	Catechin moiety					
2	4.80 (1H, d, 7.8)	83.2, d	4.56 (1H, d, 7.5)‡	82.8, d	4.87 (1H, s)	79.9, d
3	4.13 (1H, td, 8.1, 5.3)	68.8, d	3.97 (1H, td, 7.9, 5.4)	68.8, d	4.18 (1H, ddd, 4.7, 2.9, 1.4)	67.4, d
4	3.04 (1H, dd, 16.1, 5.4)<br>2.68 (1H, dd, 16.1, 8.4)	29.4, t	2.85 (1H, dd, 16.1, 5.4)<br>2.50 (1H, dd, 16.1, 8.2)	28.5, t	2.87 (1H, dd, 16.7, 4.6)<br>2.74 (1H, dd, 16.8, 2.9)	29.3, t
5	-	155.8, s	-	157.5, s	-	157.7, s
6	6.53 (1H, s)	91.5, d	5.93 (1H, d, 2.2)	96.2, d	5.92 (1H, d, 2.3)	95.9, d‡
7	-	157.9, s	-	157.8, s	-	157.9, s
8	-	106.9, s	5.85 (1H, d, 2.3)	95.4, d	5.94 (1H, d, 2.3)	96.4, d
9	-	150.6, s	-	156.9, s	-	157.4, s
10	-	103.9, s	-	100.8, s	-	100.0, s
1'	-	132.2, s	-	132.2, s	-	131.6, s
2'	6.97 (1H, d, 2.0)	115.3, d	6.84 (1H, d, 2.0)‡	115.2, d	7.31 (1H, d, 8.5)	129.1, d
3'	-	146.4, s	-	146.2, s	6.78 (1H, d, 8.6)	115.7, d
4'	-	146.5, s	-	146.2, s	-	158.0, s
5'	6.81 (1H, d, 8.1)	116.2, d	6.76 (1H, d, 8.1)	116.0, d	6.78 (1H, d, 8.6)	115.7, d
6'	6.85 (1H, dd, 8.2, 2.1)	120.3, d	6.72 (1H, dd, 8.2, 2.)	120.0, d	7.31 (1H, d, 8.5)	129.1, d
	D-ring					
1"	-	116.7, s	-	-	-	-
2"	7.22 (1H, s)	107.9, s	-	-	-	-
3"	-	142.7, s	-	-	-	-
4"	-	144.9, s	-	-	-	-
5"	6.87 (1H, s)	98.9, d	-	-	-	-
6"	-	151.0, s	-	-	-	-
